# Supplementary material for: Divergent vertebral formulae shape the evolution of axial complexity in mammals
Source: Nat Ecol Evol. 2023 Mar 6;7(3):367–81. doi: 10.1038/s41559-023-01982-5 (PMC9998275; doi:10.1038/s41559-023-01982-5)
Supplement: Supplementary file 1 — Supplementary Results, Discussion and Figs. 1–24. [file 41559_2023_1982_MOESM1_ESM.pdf]

nature ecology & evolution

Article https://doi.org/10.1038/s41559-023-01982-5

Divergent vertebral formulae shape the evolution of axial complexity in mammals

In the format provided by the authors and unedited

Supplementary Information

Divergent vertebral formulae shape the evolution of anatomical complexity in mammals

Yimeng Li[1], Andrew Brinkworth[1], Emily Green[2], Jack Oyston[1], Matthew Wills[1] and Marcello Ruta[2*]

[1]Milner Centre for Evolution, Department of Biology and Biochemistry, University of Bath, Claverton Down, Bath, BA2 7AY, UK.

[2]Joseph Banks Laboratories, Department of Life Sciences, University of Lincoln, Green Lane, Lincoln, LN6 7DL, UK.

Table of Contents

Supplementary Results

Supplementary Discussion

Supplementary Figures

Supplementary Results

Vertebral counts differ significantly between groups

Histograms of percentage distributions of TL, T, and L counts capture divergent patterns, particularly in the variable numbers of distinct modes across different groups (Supplementary Data 1). For both T and L, Chiroptera, Ferae, and Euarchonta (as well as Perissodactyla in the case of L only) reveal three dominant modes, whereas Eulipotyphla and Glires show two. Single-mode distributions for all count categories occur in Marsupialia and, to a lesser degree, in Cetartiodactyla. In Afrotheria, Xenarthra, and Cetartiodactyla, the distribution of T counts is characterized by multiple non-adjacent modes. A similar pattern features in the distribution of L counts in Afrotheria and Cetartiodactyla, except that in both groups, no discontinuities intervene between modes (Supplementary Data 1).

Changes in complexity are concentrated in younger branches

The TL Brillouin and evenness indices tend to increase from basal to terminal branches in most major groups (Fig. 4b, c). This pattern is exemplified by some lineages within Xenarthra (e.g., giant and long-nosed armadillos), Afrotheria (e.g., hyraxes; elephant shrews), most Eulipotyphla (solenodons represent one of the few exceptions), numerous Chiroptera (mostly evening bats), Perissodactyla (several African equids), Ferae (e.g., felids; civets and genets; mongooses; canids; some ursids; various lineages within skunks, weasels, martens, badgers, and otters), most 'Artiodactyla' and Cetacea, as well as the majority of Primates and Glires. Interspersed with increases are also instances of decreases, both along terminal branches as well as near the roots of various subclades within various major groups. Decreases characterize Monotremata, Marsupialia (common and hairy-nosed wombats; marsupial moles), Xenarthra (hairy, fairy, and

banded armadillos; two- and three-toed sloths), Afrotheria (sirenians; proboscideans; golden moles), Cetacea (South American river dolphins; pygmy right whale), ‘Artiodactyla’ (giraffids; some wild cattle; small antelopes; duikers), Primates (hominoids; howler, spider, and woolly monkeys), and Glires (pikas; Old World porcupines; beavers). The distribution of increases and decreases for the unstandardized T:L (Fig. 4d) is approximately the mirror opposite of TL evenness because for increasingly similar T and L counts, both indices approach 1.00.

TL counts correlate negatively with T:L ratios

In addition to the significant (albeit weak) negative correlation between TL counts and T:L ratios across the whole taxon sample (Fig. 5a; Supplementary Fig. 1; residual SE = 0.2616; adjusted R^2 = 0.0276; F-statistic = 9.188; df = 287; $P \sim 0.0026$), we disentangle the contributions of each of the T and L counts to the relative size of the thoracic and lumbar domains (T:L ratios). Thus, independent contrasts analyses show T:L to be positively correlated with T (residual SE = 0.2751; adjusted R^2 = 0.1487; F-statistic = 47.81; df = 267; P = 3.461e-11), with a loess curve marked by a steep positive slope for values of T up to 11-12 and a plateau throughout the range of the largest T counts (Fig. 5b). The T:L ratios become increasingly more dispersed for T ≥ 15, highlighting diverging patterns of axial elongation in groups such as Afrotheria and Xenarthra. Furthermore, T:L is negatively correlated with L (residual SE = 0.2344; adjusted R^2 = 0.2971; F-statistic = 123.1; df = 288; $P < 2.2e\text{-}16$), with a loess curve resembling a power function with negative scaling exponent (Fig. 5c). For L ≥ 9, the dispersion of T:L values decreases noticeably, once again as a result of the highly divergent patterns of trunk elongation within Cetacea.

TL counts correlate positively with complexity

Whereas the standardized contrasts of TL counts correlate positively with both TL Brillouin and TL evenness (Supplementary Figs 2, 3), only in the case of TL Brillouin is the correlation significant (residual SE = 0.01444; adjusted R^2 = 0.03852; F-statistic = 12.54; df = 287; *P* = 0.0004652). Conversely, the correlation is negative for the standardized contrasts of the presacral counts (Supplementary Figs 4, 5), and significant only for CTL evenness (residual SE = 0.01158; adjusted R^2 = 0.05302; F-statistic = 17.13; df = 287; *P* = 0.0004603).

Thoracolumbar complexity and domains

As shown in Supplementary Figs 6, 7, at the lower end of the range of T:L ratios, we encounter mammals that exhibit the most complex thoracolumbar regions, both in terms of the relative numerical richness of thoracic and lumbar elements and in the degree to which such elements are distributed equitably. Most of these are Cetacea, but some species in other clades also occur within this range (e.g., Laotian rock rat and naked mole-rat among Glires; hero shrew among Eulipotyphla; indri, Eastern woolly lemur, and weasel sportive lemur among Primates; koala among Marsupialia). In contrast, the upper end of the of the range of T:L ratios is dominated almost exclusively by some members of Afrotheria, Xenarthra, and some Perissodactyla.

Changes in complexity are both directional and sustained

We present results of subclade tests applied to several of the more speciose groups to demonstrate the impact of group size on the distribution of skewness across our study sample (Supplementary Table 5). Due to space limitations, we examine two

representative indices only, namely TL Brillouin and evenness, in all tested groups. Our first case-study concerns Euarchonta (Extended Data Fig. 3a, b), partitioned into a paraphyletic array of early diverging lineages (treeshrews; colugos; lemurs; bushbabies; tarsiers) and two monophyletic groups, namely New World monkeys and Old World monkeys plus apes. Application of the subclade test shows that, for both TL Brillouin and evenness, SCW forms a substantial proportion of the total skewness (67.9%), followed by SCH (30% and 29%, respectively, for TL Brillouin and evenness), and a negligible amount of SCB (2.1% and 3.1%). In the case of two groups, Carnivora (Extended Data Fig. 3c, d) and Cetartiodactyla (Extended Data Fig. 3e, f), we encounter an unusual, but not unexpected outcome in the distribution of percentages allocated to the three main components of total skewness. In some circumstances (e.g., uneven sampling), the subclade test may return negative percentage values as well as percentages that exceed 100%. To the best of our knowledge, there is no immediate solution to this problem and indeed, interpreting these percentages becomes arduous. Following reference[45], we consider the sum of the absolute values of the SCB, SCH, and SCW percentages and express the contribution of each as a proportion of that sum. In the case of Carnivora, partitioned into Caniformia and Feliformia (Extended Data Fig. 3c, d), the SCB, SCH, and SCW percentages are, respectively, -6.8%, 21.8%, and 85% for TL Brillouin, and -7.2%, 22.5%, and 84.7% for TL evenness. After conversion, the proportions of skewness attributed to SCW become 74.8% and 74%, respectively. In Cetartiodactyla, partitioned into Cetacea and 'Artiodactyla' (Extended Data Fig. 3e, f), skewness percentages are as follows: SCB = -12.3%, SCH = -66.1%, and SCW = 178.4 % for TL Brillouin; SCB = -6.3%, SCH = -43.8%, and SCW = 150.1 % for TL evenness. Using the same correction procedure, we find that SCW contributes 69.4% and 74.9% to total skewness for the two indices. A subclade test applied to Afrotheria, partitioned into

Paenungulata and Afroinsectiphilia (Extended Data Fig. 3g, h), returns the following percentages: SCB = 5.8%, SCH = 25.5%, and SCW = 68.7 % for TL Brillouin; SCB = 7.4%, SCH = 17.6%, and SCW = 75 % for TL evenness. As a final case-study, we use Chiroptera, partitioned into Yinpterochiroptera (flying foxes; fruit bats; some families of small-sized bats) and Yangochiroptera (remaining lineages of small-sized bats), the latter divided into two clades labelled as ‘A’ and ‘B’ for convenience (Extended Data Fig. 3i, j). In the case of both indices, SCW (50.8% for TL Brillouin; 48.2% for TL evenness) differs only marginally from SCH (47.2% and 49.5%), and both SCB values are negligible (2% and 2.3%).

Supplementary Discussion

Hypothesis $H_0$1: vertebral count variation

Our findings support recent efforts to disprove the notion that presacral counts are largely conserved in extant mammals[46,47,48,49,52,53,62]. We demonstrate that such counts differ significantly between groups and reveal greater variation than acknowledged hitherto. The evolutionary, ecological, and functional correlates of this variation[47,52,57,62,63,64,65] remain a key target for future research. Afrotheria, Xenarthra, Chiroptera, Perissodactyla, Cetartiodactyla, and Glires are among the groups with the greatest variation in T and L (Supplementary Data 1) and feature in a large proportion of all significant pair-wise comparisons between group-specific counts (Supplementary Table 1). Significantly, they also differ in terms of species richness, with Chiroptera and Glires as the most diverse clades and Perissodactyla as the least diverse. In this context, however, it is noteworthy that other speciose clades, such as Eulipotyphla and Euarchonta, appear in many fewer pair-wise comparisons – alongside Xenarthra and Cetartiodactyla (L counts) and Perissodactyla (T counts) in the case of Eulipotyphla, and alongside Cetartiodactyla (CTL, TL, and L counts), Afrotheria and Perissodactyla (TL and T counts), and Xenarthra (L counts) in the case of Euarchonta (Supplementary Table 1). These results suggest that taxonomic diversity may not adequately predict variation in thoracolumbar counts and a formal test of this proposition is part of our work in progress. The smallest ranges in T counts occur in Eulipotyphla (3) and Perissodactyla (2), the largest in Xenarthra (15), Afrotheria (10), Glires (9), and Cetartiodactyla (8). Xenarthra, Ferae, and Perissodactyla exhibit the smallest ranges in L counts (3 each), whereas Cetartiodactyla (30), Eulipotyphla and Glires (7 each), and Afrotheria and Euarchonta (6 each) show the largest (Supplementary Data 1). Most strikingly, a TL count of 19, generally considered to be widespread in extant mammals[48,49], features in only ~57% of species in our data,

mostly among Marsupialia, ‘Artiodactyla’, Euarchonta, Eulipotyphla, and Glires. TL counts ≥ 25 are common in Cetacea but also occur in some Afrotheria (e.g., hyraxes) and Xenarthra (e.g., two-toed sloths). TL counts ≤ 16 are documented in some Xenarthra (chiefly armadillos) and in a broad cross-section of Chiroptera (especially evening bats, as well as some slit-faced, New World leaf-nosed, false vampire, and fruit bats).

The distribution of TL increases and decreases (Fig. 4a) across the phylogeny invites a consideration of ecological and functional drivers of axial regionalization[52,62]. In some highly speciose groups, especially Glires, decreases are widespread whereas increases are generally confined to individual species or clades. One remarkable example of such clades is Nesomyinae, or Malagasy rodents (Malagasy short-tailed, tufted-tailed, and white-tailed rats), a small but morphologically diverse subfamily of arboreal, cursorial, and fossorial species with a wide range of body proportions and foraging habits, and including spectacular examples of convergence with phylogenetically distant groups, such as rabbits, voles, and mice. Another instance of thoracolumbar increase occurs in the tribe Akodontini (South America grass mice; crimson-nosed rats; brucies; shrew-mice; burrowing mice; giant rats; swamp rats), a speciose clade of New World rodents adapted to diverse environments, from tropical forests and altiplanos to salt marshes, grasslands, and deserts. In other groups with similarly infrequent TL increases, these are also usually associated with specialised locomotory modes (e.g., gliding; suspensory; tree-climbing) and lifestyles (e.g., fossoriality)[46,47,52,62]. Notable examples include early diverging clades of Euarchonta (e.g., treeshrews; colugos; several true lemurs), some New World primates (e.g., howler monkeys; tamarins), Marsupialia (mostly opossums), and Eulipotyphla (e.g., various lineages of Asiatic moles; hero shrews). In striking contrast to most other groups, Carnivora display ubiquitous (albeit negligible) TL increases but comparatively fewer decreases. Such decreases occur mostly among

raccoons, weasels, and otters, but the significance of this distribution is unclear. Lastly, Cetacea stand out relative to other clades in revealing intricate patterns of internested increases and decreases within toothed and baleen whales. This pattern is particularly evident among oceanic dolphins, many of which feature examples of niche partitioning and foraging specializations in recently diverged sister taxa.

Hypothesis $H_0 2$: complexity trends

Despite a general tendency for TL Brillouin and evenness to increase over time, the temporal trends exhibited by individual groups reveal conflicting patterns. For example, in Afrotheria, Xenarthra, Ferae, and Perissodactyla, the regression slopes from analyses of ancestral node estimates vs. node ages are non-significant (Supplementary Table 4). In those groups, the temporal distribution of ancestral complexity estimates is markedly heteroscedastic, thereby potentially obfuscating or weakening any underlying pattern. In contrast, in groups characterized by smaller variances in complexity values (e.g., Glires), trends emerge more distinctly (Fig. 6a, c, e, g; Extended Data Fig. 2a, c; Supplementary Figs 8–13; Supplementary Table 4). Lastly, results from the subclade tests highlight the influence of group size on the balance between passive and driven processes of complexity change. Whereas analyses of the entire species sample (Supplementary Figs 20–24; Supplementary Table 5) suggest the prevalence of a diffusive model of complexity change, tests carried out on some of the most speciose clades point to driven trends (Extended Data Fig. 3). Taken together, our results support sustained and directional changes in the evolution of axial regionalization, thereby providing the only other tested case-study of a driven complexity trend in a major clade[25].

One major conclusion from our investigation is that estimates of complexity at the internal nodes of the phylogeny correlate positively and significantly with the magnitude

of changes along the branches subtended by those nodes (Fig. 6b, d, f, h; Extended Data Fig. 2b, d; Supplementary Figs 14–19; Supplementary Table 4), a pattern that is replicated in all major groups. In this respect, therefore, changes in complexity mirror those of other macroevolutionary traits, particularly body size, where higher initial values tend to be associated with further downstream increases in descendant lineages[94] and have been interpreted as evidence for adaptive evolution[94]. Whereas tests of the adaptive role of complexity[52,62] are outside the scope of this Article, our results appear *consistent* with such a role, at least in certain domains of the phylogeny. For example, increasing complexity characterizes branches that either subtend, or immediately precede, major ecological and environmental shifts, such as the transition from land to water in Cetacea and the conquest of the air in Chiroptera (Fig. 4b, c; Extended Data Fig.1a, b). Other increases are associated with structural and functional innovations, a classical example of which is provided by Perissodactyla. Among extant Perissodactyla, both equids and tapirids (but not rhinocerotids) feature increases in both thoracolumbar counts and complexity (Fig. 4a-c). In the case of equids, the evolutionary transition from small, forest-dwelling taxa to the mid- and large-sized species of today's savannahs and grasslands was accompanied by the emergence of multiple traits associated with cursoriality. These traits include a dorsostable column, elongate limbs, reduction in digit numbers, and digit elongation[95]. Like their extant counterparts, early equids were also built for speed, with several lineages independently acquiring various degrees of axial elongation. Following the radiation of equids into open habitats and the increasing demands for sustained speed, axial elongation became co-opted for enhanced biomechanical efficiency alongside a suite of novel morphofunctional characteristics (e.g., reduction and subsequent loss of sagittal flexibility at the thoracic-lumbar boundary; development of intervertebral ligaments and interlocking vertebral joints[95]).

Hypothesis $H_0 3$: evolutionary rates

For all complexity indices, we find unequivocal support for multiple-rate models of evolution[69,70]. These results are in broad agreement with those of recent analyses that have employed linear measurements and geometric morphometric data to examine axial regionalization. Using a representative sample of extant mammals and their immediate outgroups among non-mammalian synapsids, a recent study[54] has uncovered two major phases in the evolution of the vertebral column, viz. an increase in morphological differentiation *between* regions followed by augmented integration *within* regions[54]. The same study has challenged previous scenarios of gradual increases in column complexity over time[58], replacing them with a model of stepwise shifts between optima[54]. Although our approach to measuring axial complexity lacks the detail of morphological studies, we retrieve a similar pattern of stepwise shifts in rates of complexity change, typically in the form of pulse-like, inter-nested rate increases and decreases. Numerous increases with high posterior probabilities characterize younger branches of the phylogeny (Extended Data Figs 4, 5) and, at least in some groups (e.g., Cetacea), are consistent with episodes of rapid divergence in axial patterning between closely related species. In contrast, widespread and sustained decreases are consistent with evolutionary tendencies towards stable optima, such as may be represented by conserved vertebral constructions.

Supplementary Figures

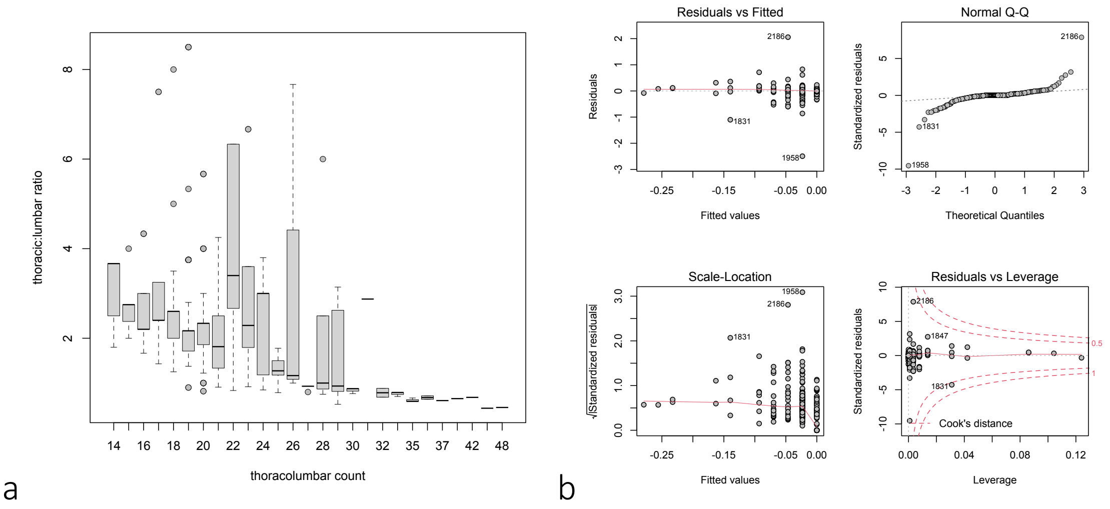

Supplementary Fig. 1 | Independent contrasts ('brunch' algorithm) analysis of the relationship between thoracic:lumbar ratios and thoracolumbar counts in 1,136 extant mammal species. a, Bivariate scatterplot of ratios vs. counts; for any given count, the distribution of ratios is presented in the form of a box and whisker plot, inclusive of median values, minimum and maximum values (excluding outliers), interquartile ranges, and outlying data points. **b**, Diagnostic plots associated with the independent contrasts analysis (see Supplementary Table 3).

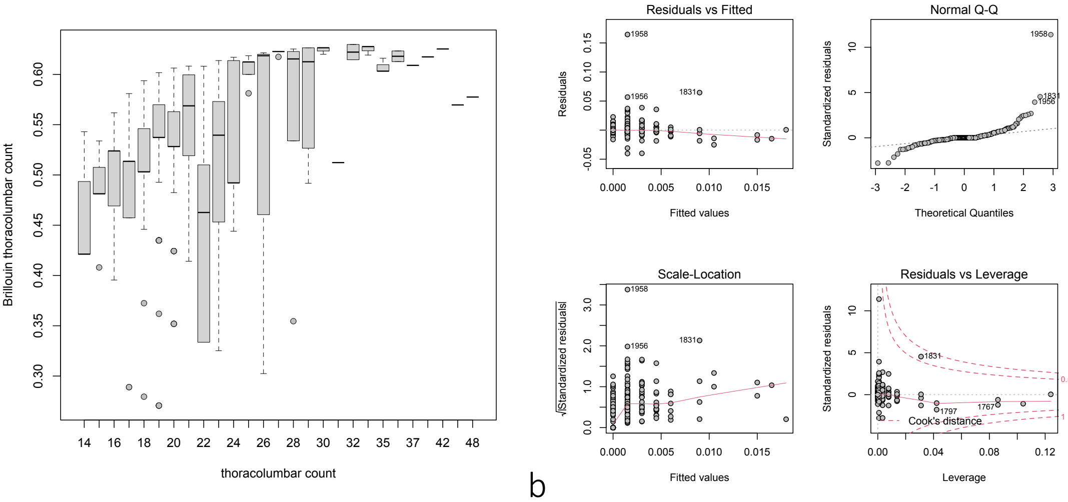

a b

Supplementary Fig. 2 | Independent contrasts ('brunch' algorithm) analysis of the relationship between the Brillouin index of the thoracolumbar region and the thoracolumbar counts in 1,136 extant mammal species. For explanations, see caption of Supplementary Fig. 1.

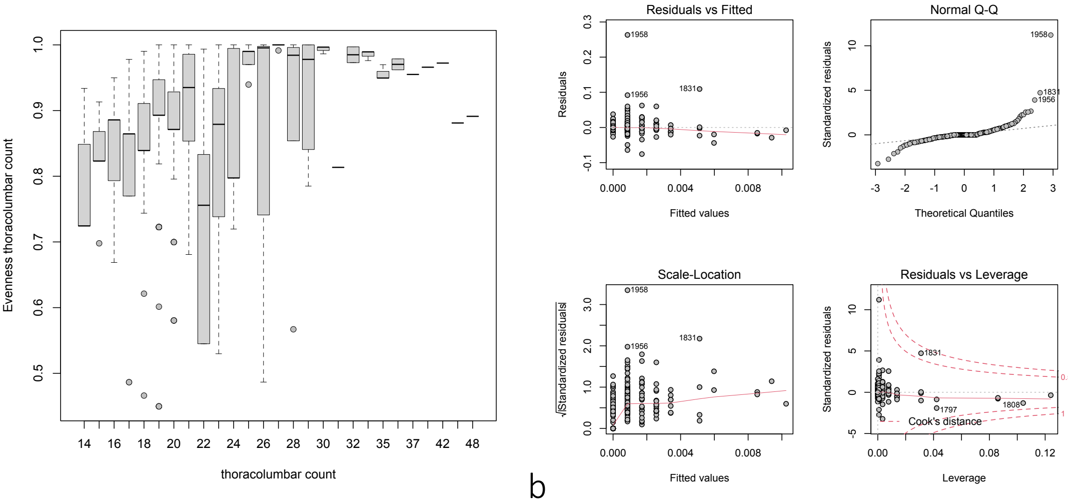

a b

Supplementary Fig. 3 | Independent contrasts ('brunch' algorithm) analysis of the relationship between the evenness index of the thoracolumbar region and the thoracolumbar counts in 1,136 extant mammal species. For explanations, see caption of Supplementary Fig. 1.

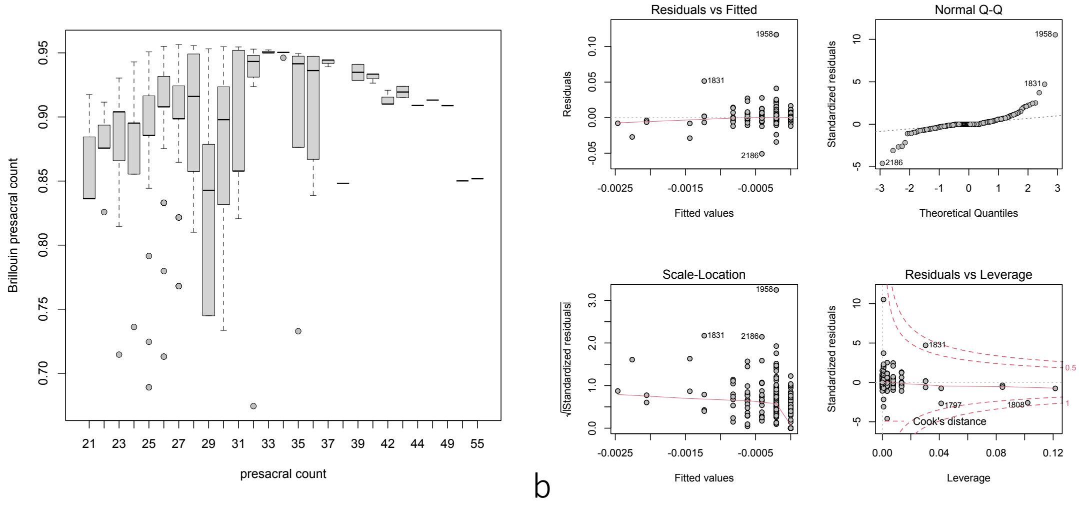

Supplementary Fig. 4 | Independent contrasts ('brunch' algorithm) analysis of the relationship between the Brillouin index of the presacral region and the presacral counts in 1,136 extant mammal species. For explanations, see caption of Supplementary Fig. 1.

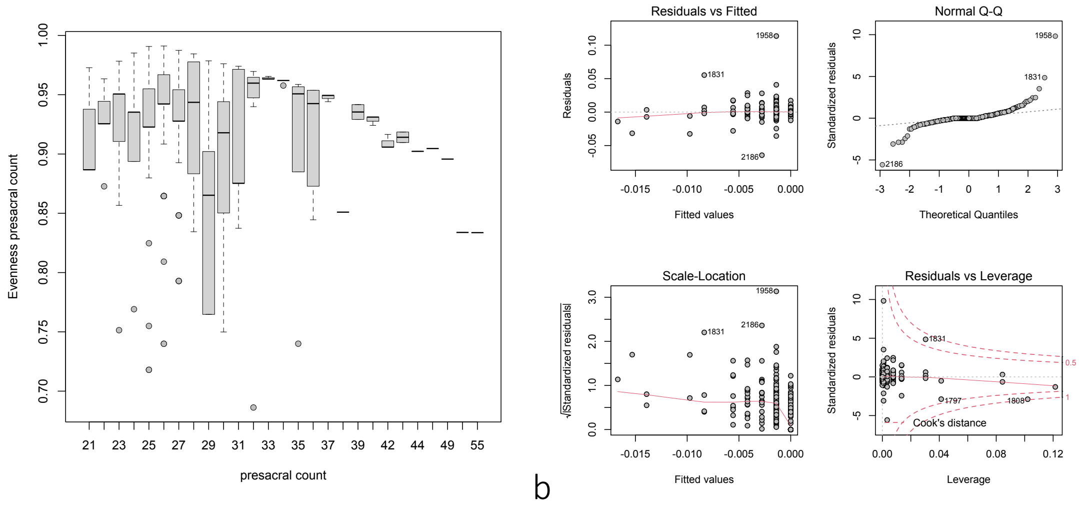

Supplementary Fig. 5 | Independent contrasts ('brunch' algorithm) analysis of the relationship between the evenness index of the presacral region and the presacral

counts in 1,136 extant mammal species. For explanations, see caption of Supplementary Fig. 1.

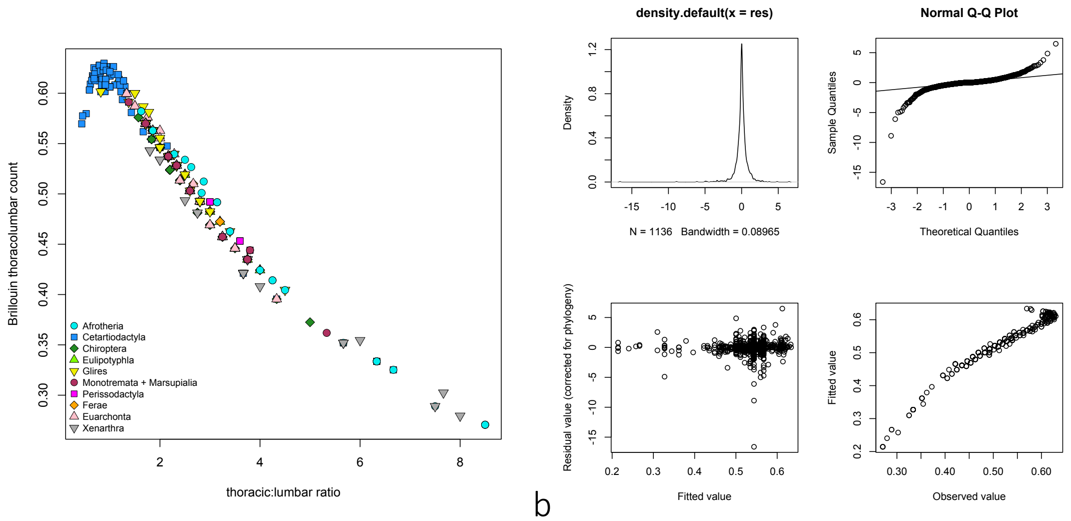

a b

Supplementary Fig. 6 | Phylogenetic generalised least squares (PGLS) analysis of the relationship between the Brillouin index of the thoracolumbar region and the thoracic:lumbar ratios in 1,136 extant mammal species. a, Bivariate scatterplot of index vs. ratios; the data points are shown in different colours and symbols associated with major mammal groups. **b**, Diagnostic plots associated with the PGLS analysis (see Supplementary Table 3).

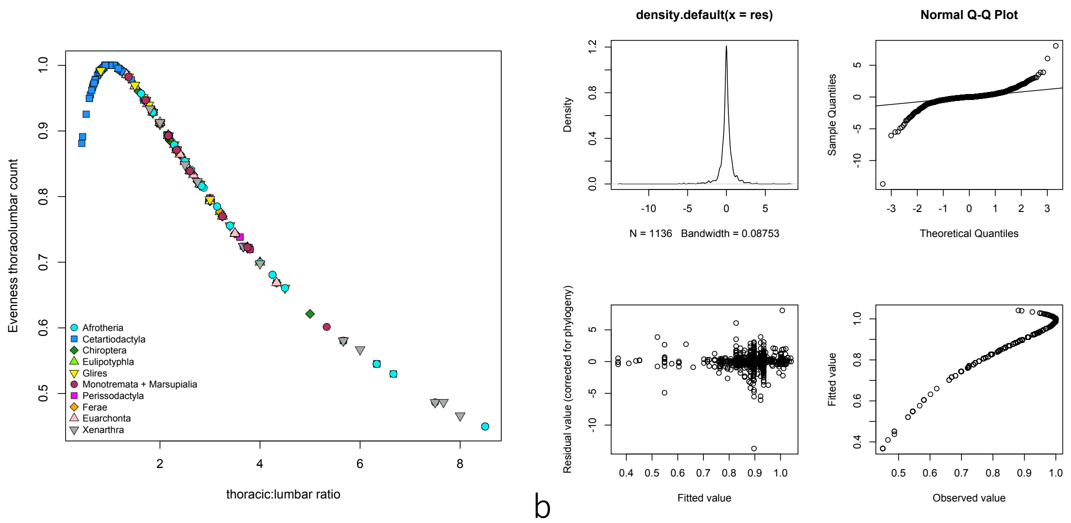

a b

Supplementary Fig. 7 | Phylogenetic generalised least squares (PGLS) analysis of the relationship between the evenness index of the thoracolumbar region and the thoracic:lumbar ratios in 1,136 extant mammal species. For explanations, see caption of Supplementary Fig. 6.

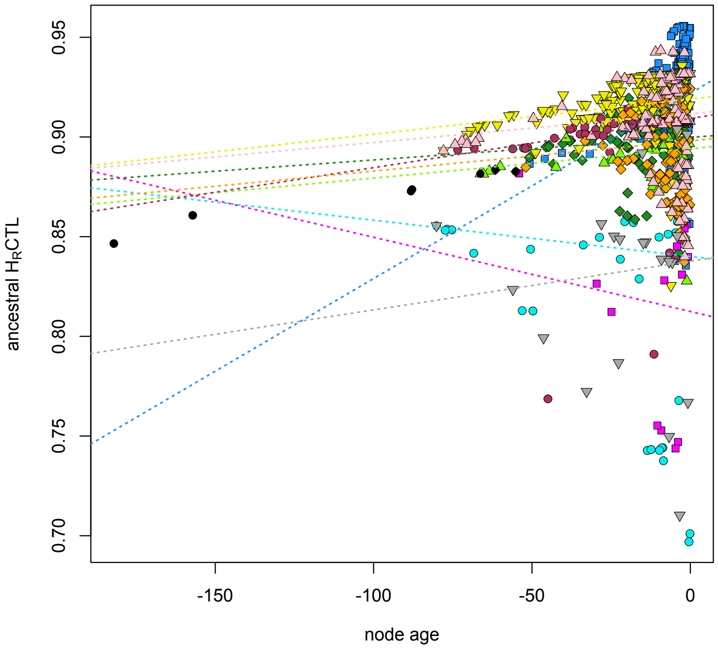

Supplementary Fig. 8 | Robust linear regression analysis of the relationship between maximum likelihood node estimates for the presacral Brillouin index and node ages. Bivariate scatterplot of index estimates at the internal nodes of the mammal phylogeny vs. node ages (in millions of years) with superimposed regression lines for major mammal groups (see Supplementary Table 4).

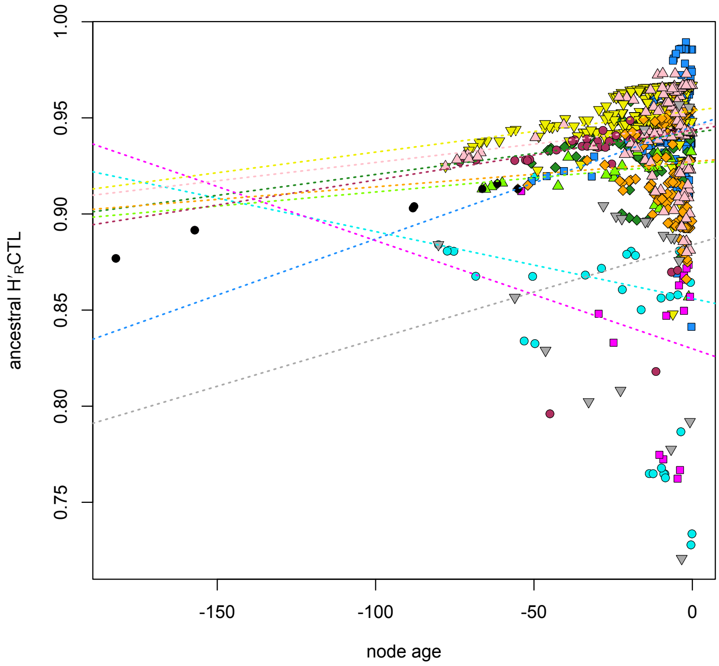

Supplementary Fig. 9 | Robust linear regression analysis of the relationship between maximum likelihood node estimates for the presacral evenness index and node ages. For explanations, see caption of Supplementary Fig. 8.

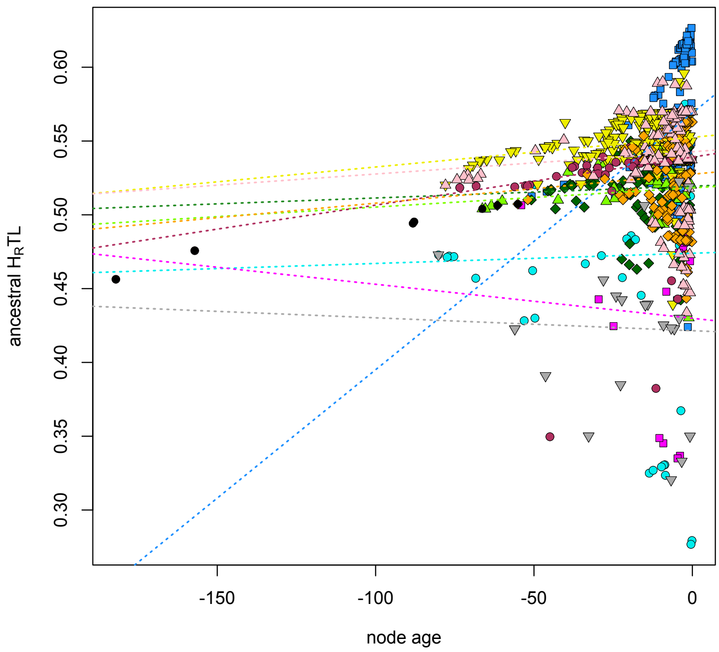

Supplementary Fig. 10 | Robust linear regression analysis of the relationship between maximum likelihood node estimates for the thoracolumbar Brillouin index and node ages. For explanations, see caption of Supplementary Fig. 8.

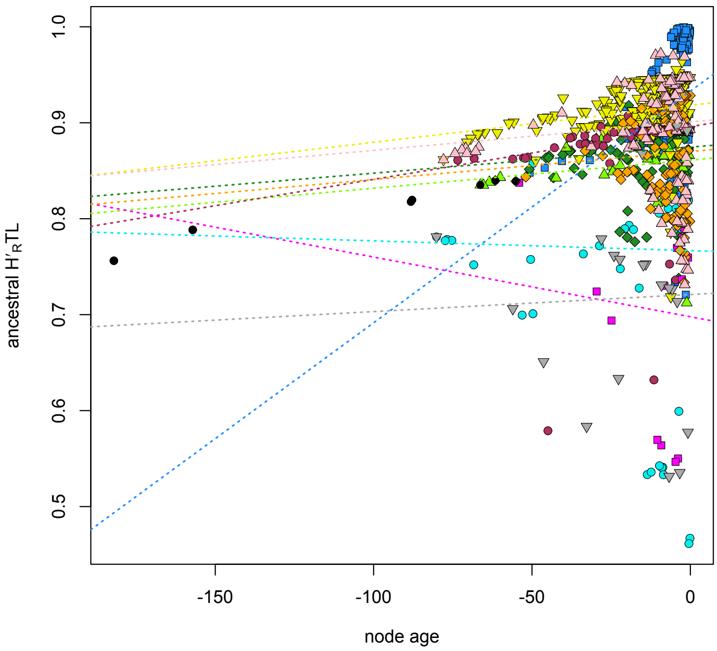

Supplementary Fig. 11 | Robust linear regression analysis of the relationship between maximum likelihood node estimates for the thoracolumbar evenness index and node ages. For explanations, see caption of Supplementary Fig. 8.

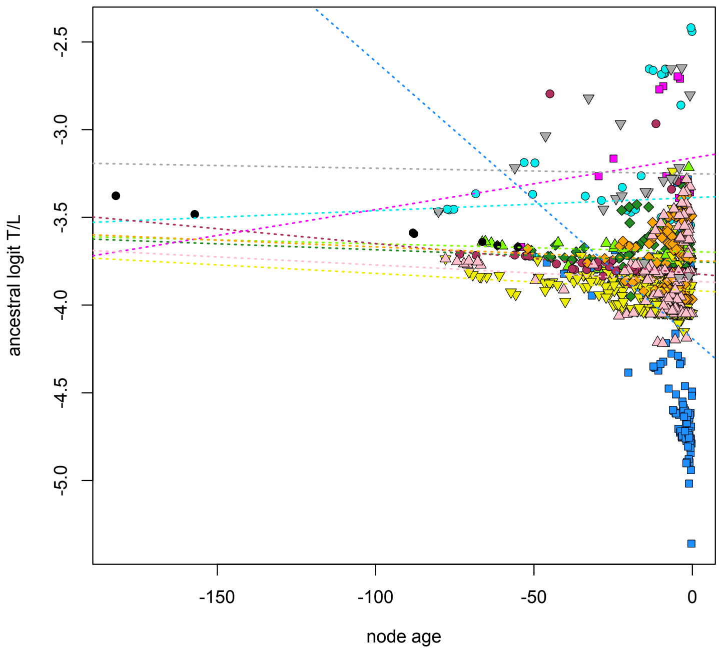

Supplementary Fig. 12 | Robust linear regression analysis of the relationship between maximum likelihood node estimates for the logit-transformed thoracic:lumbar ratios and node ages. For explanations, see caption of Supplementary Fig. 8.

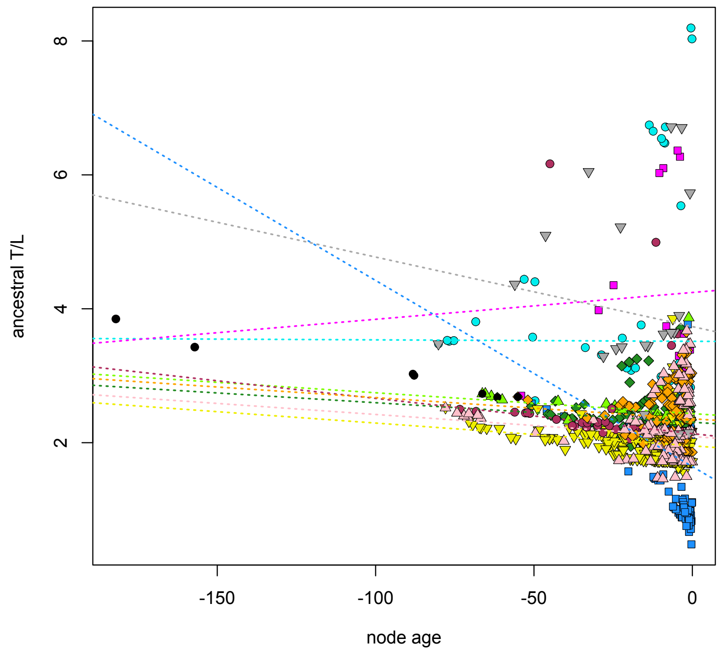

Supplementary Fig. 13 | Robust linear regression analysis of the relationship between maximum likelihood node estimates for the unstandardized thoracic:lumbar ratios and node ages. For explanations, see caption of Supplementary Fig. 8.

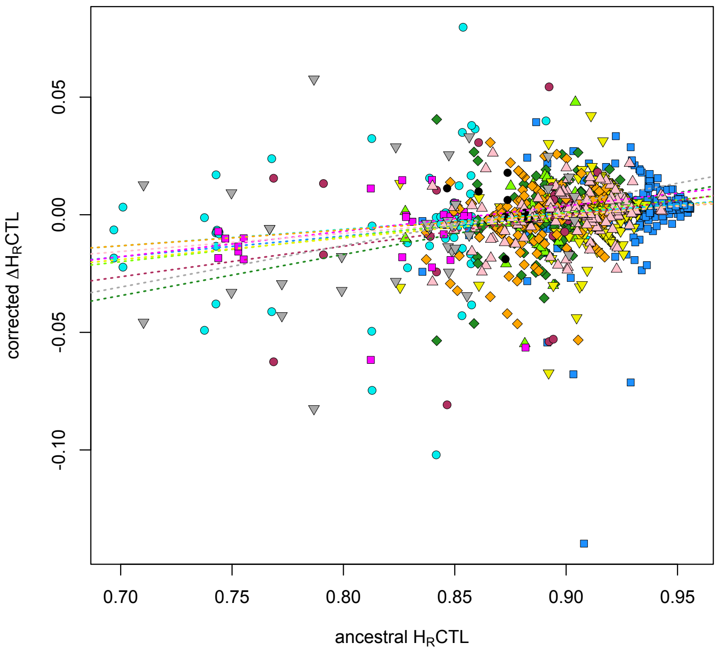

Supplementary Fig. 14 | Robust linear regression analysis of the relationship between descendant-ancestor differences (corrected for the regression to the mean) for the presacral Brillouin index and maximum likelihood node estimates. Bivariate scatterplot of descendant-ancestor index differences along the tree branches (descendant node value minus ancestor node value) vs. index estimates at the internal nodes of the mammal phylogeny (in millions of years) with superimposed regression lines for major mammal groups (see Supplementary Table 4).

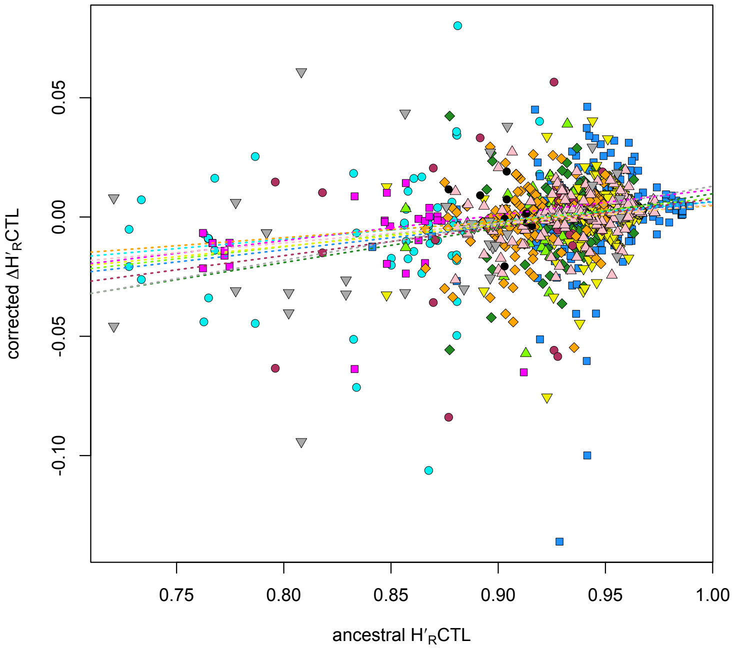

Supplementary Fig. 15 | Robust linear regression analysis of the relationship between descendant-ancestor differences (corrected for the regression to the mean) for the presacral evenness index and maximum likelihood node estimates. For explanations, see caption of Supplementary Fig. 14.

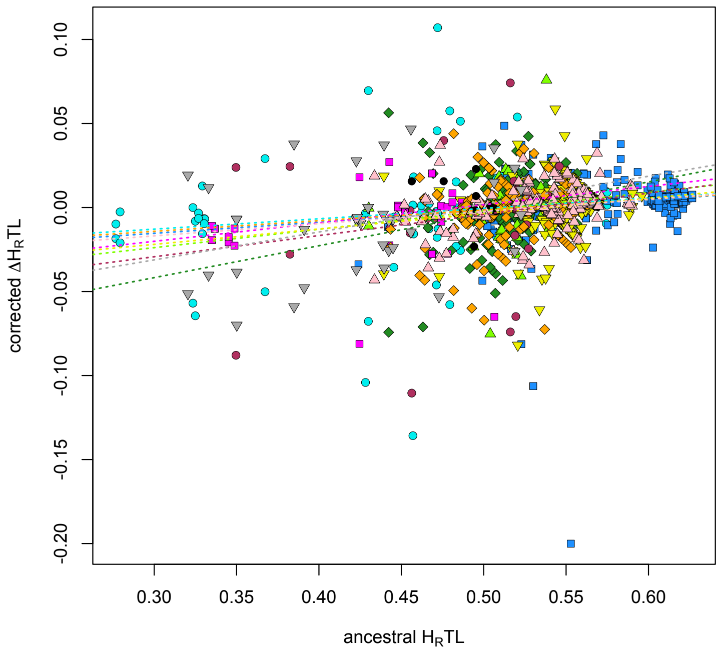

Supplementary Fig. 16 | Robust linear regression analysis of the relationship between descendant-ancestor differences (corrected for the regression to the mean) for the thoracolumbar Brillouin index and maximum likelihood node estimates. For explanations, see caption of Supplementary Fig. 14.

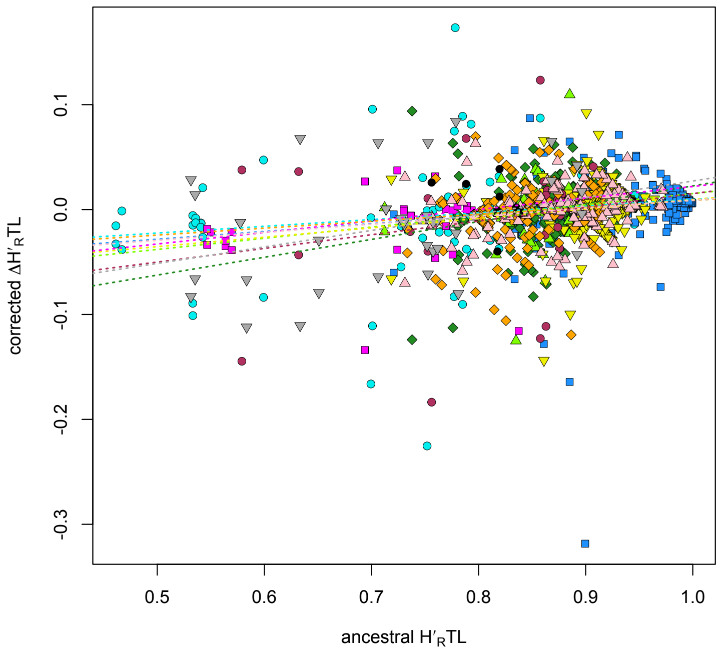

Supplementary Fig. 17 | Robust linear regression analysis of the relationship between descendant-ancestor differences (corrected for the regression to the mean) for the thoracolumbar evenness index and maximum likelihood node estimates. For explanations, see caption of Supplementary Fig. 14.

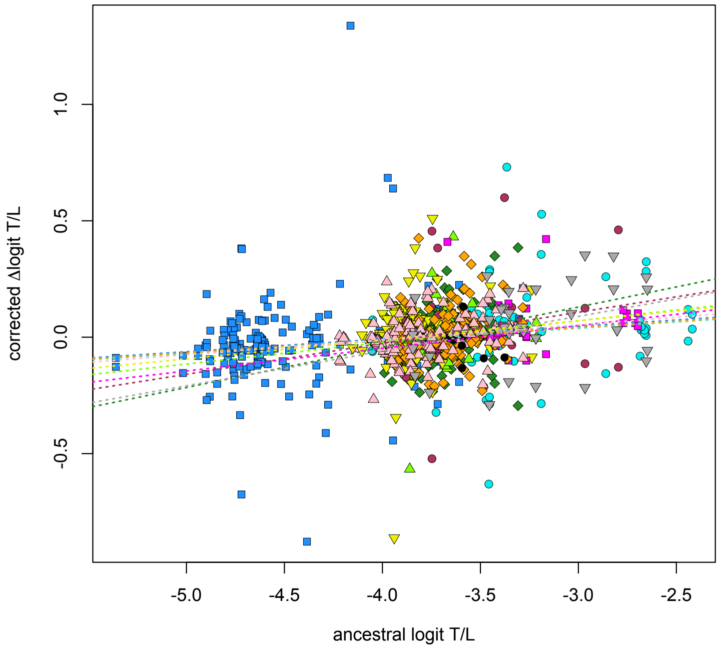

Supplementary Fig. 18 | Robust linear regression analysis of the relationship between descendant-ancestor differences (corrected for the regression to the mean) for the logit-transformed thoracic:lumbar ratios and maximum likelihood node estimates. For explanations, see caption of Supplementary Fig. 14.

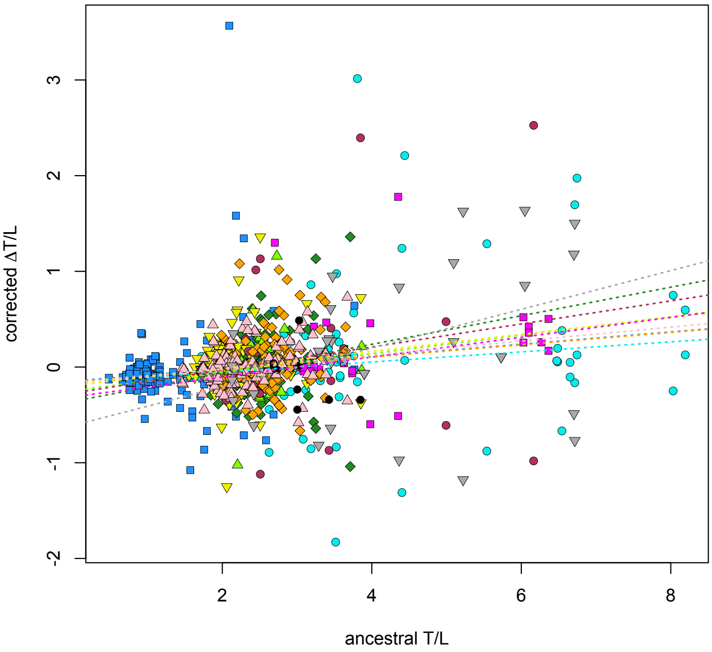

Supplementary Fig. 19 | Robust linear regression analysis of the relationship between descendant-ancestor differences (corrected for the regression to the mean) for the unstandardized thoracic:lumbar ratios and maximum likelihood node estimates. For explanations, see caption of Supplementary Fig. 14.

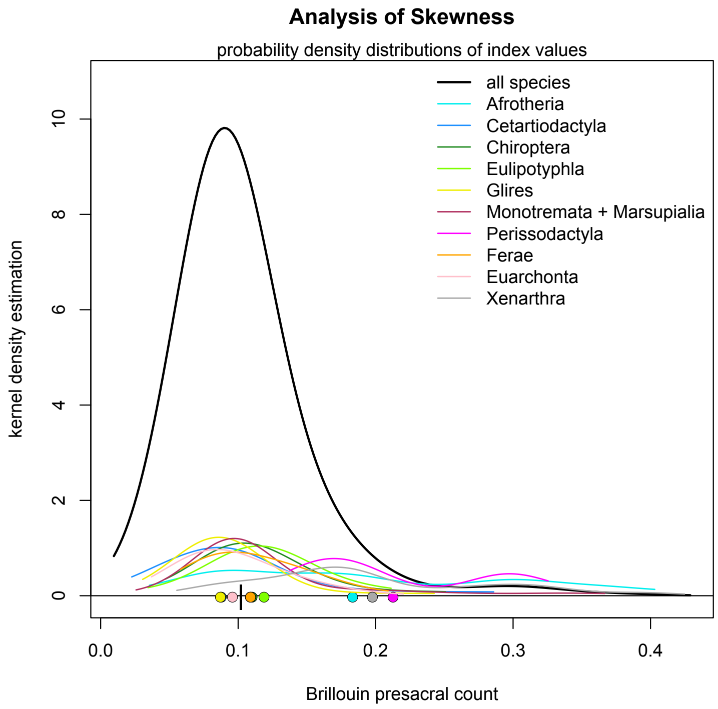

Supplementary Fig. 20 | Results of skewness partitioning test applied to the presacral Brillouin index for the entire mammal species sample. In the plot, the colour-coded thin lines represent the probability density distributions of the index values in each major mammal group, whereas the thick black line shows the probability density distribution for the entire sample. The mean values of the individual groups are represented by colour-coded circles, whereas the mean value of the entire distribution is marked by a black vertical bar (see Supplementary Table 5).

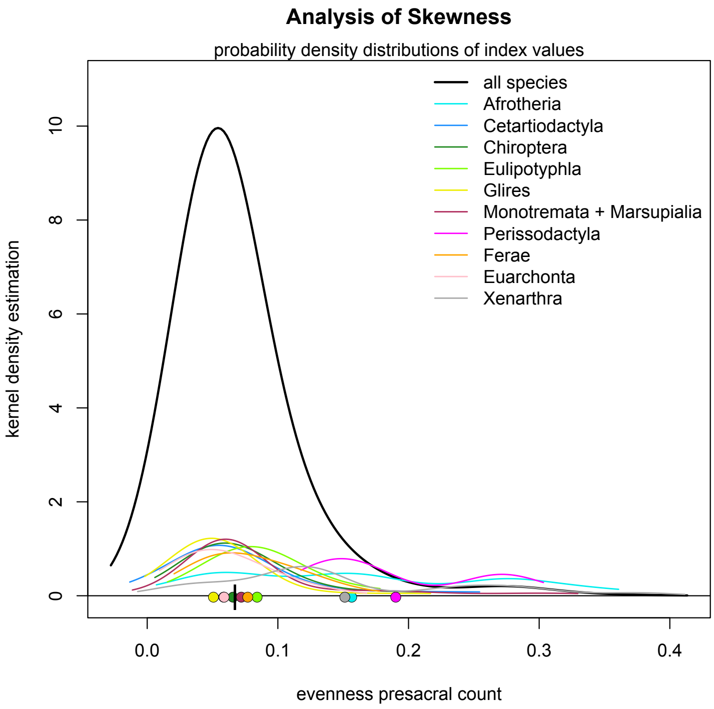

Supplementary Fig. 21 | Results of skewness partitioning test applied to the presacral evenness index for the entire mammal species sample. For explanations, see caption of Supplementary Fig. 20.

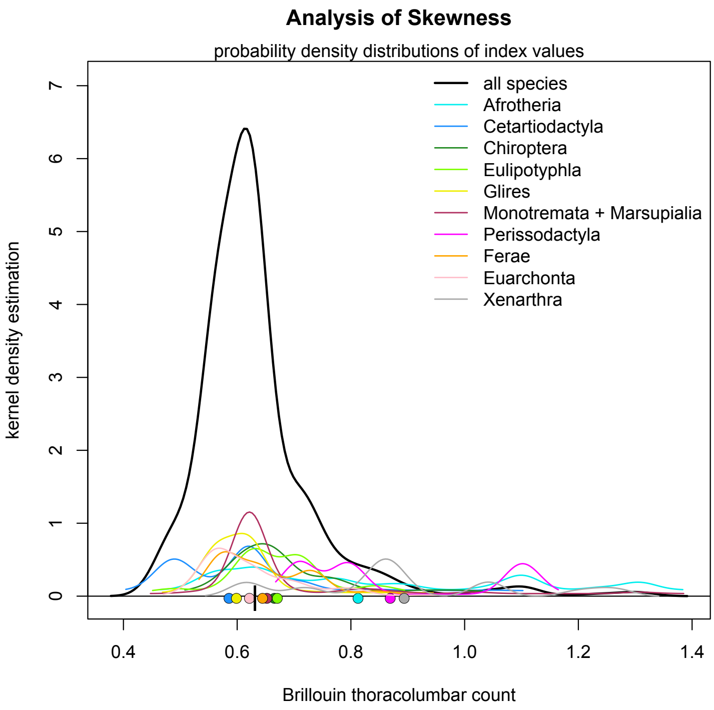

Supplementary Fig. 22 | Results of skewness partitioning test applied to the thoracolumbar Brillouin index for the entire mammal species sample. For explanations, see caption of Supplementary Fig. 20.

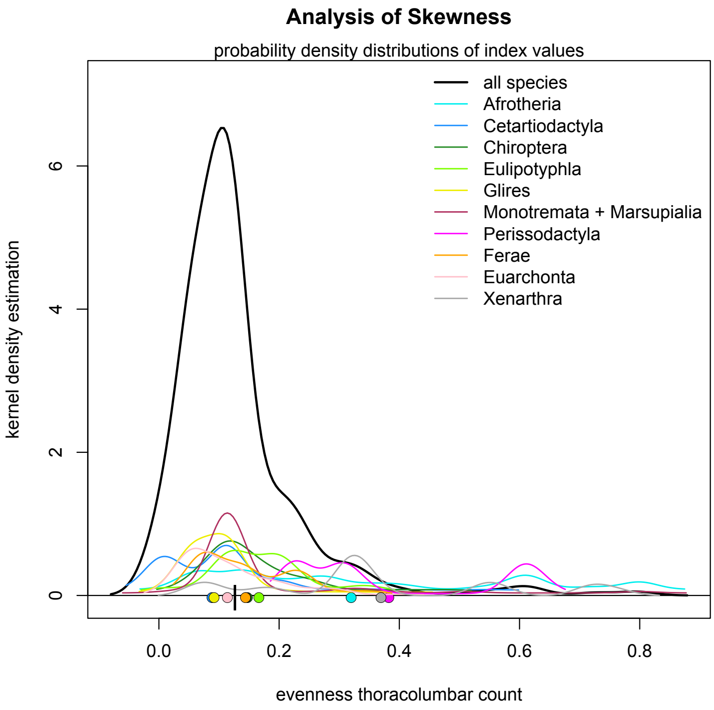

Supplementary Fig. 23 | Results of skewness partitioning test applied to the thoracolumbar evenness index for the entire mammal species sample. For explanations, see caption of Supplementary Fig. 20.

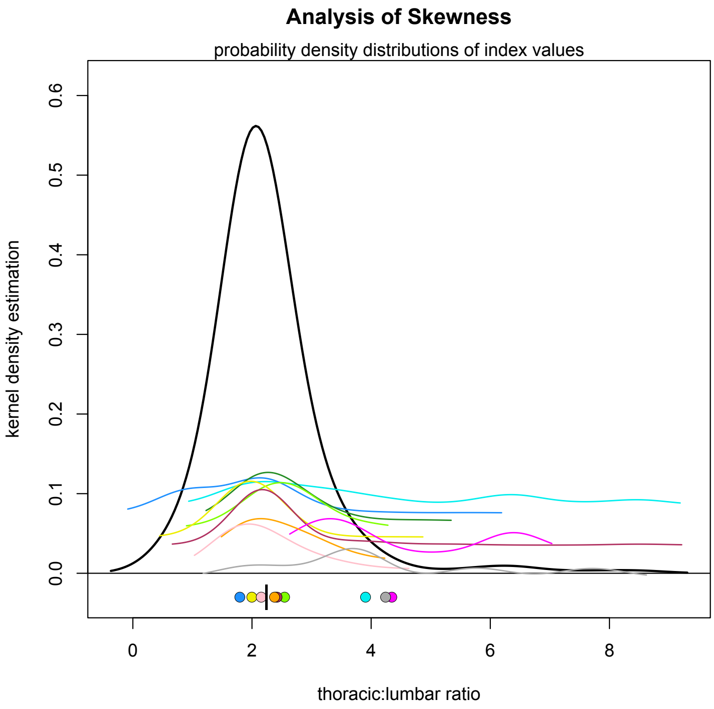

Supplementary Fig. 24 | Results of skewness partitioning test applied to the unstandardized thoracic:lumbar ratio for the entire mammal species sample. For explanations, see caption of Supplementary Fig. 20.
