# Supplementary material for: Divergent vertebral formulae shape the evolution of axial complexity in mammals
Source: Nat Ecol Evol. 2023 Mar 6;7(3):367–81. doi: 10.1038/s41559-023-01982-5 (PMC9998275; doi:10.1038/s41559-023-01982-5)
Supplement: Supplementary file 2 — Reporting Summary [file 41559_2023_1982_MOESM2_ESM.pdf]

nature portfolio

Corresponding author(s): Marcello Ruta

Last updated by author(s): Nov 27, 2022

Reporting Summary

Nature Portfolio wishes to improve the reproducibility of the work that we publish. This form provides structure for consistency and transparency in reporting. For further information on Nature Portfolio policies, see our Editorial Policies and the Editorial Policy Checklist.

Statistics

For all statistical analyses, confirm that the following items are present in the figure legend, table legend, main text, or Methods section.

n/a	Confirmed	
☐	☒	The exact sample size (n) for each experimental group/condition, given as a discrete number and unit of measurement
☐	☒	A statement on whether measurements were taken from distinct samples or whether the same sample was measured repeatedly
☐	☒	The statistical test(s) used AND whether they are one- or two-sided<br>*Only common tests should be described solely by name; describe more complex techniques in the Methods section.*
☐	☒	A description of all covariates tested
☐	☒	A description of any assumptions or corrections, such as tests of normality and adjustment for multiple comparisons
☐	☒	A full description of the statistical parameters including central tendency (e.g. means) or other basic estimates (e.g. regression coefficient) AND variation (e.g. standard deviation) or associated estimates of uncertainty (e.g. confidence intervals)
☐	☒	For null hypothesis testing, the test statistic (e.g. F, t, r) with confidence intervals, effect sizes, degrees of freedom and P value noted<br>*Give P values as exact values whenever suitable.*
☐	☒	For Bayesian analysis, information on the choice of priors and Markov chain Monte Carlo settings
☒	☐	For hierarchical and complex designs, identification of the appropriate level for tests and full reporting of outcomes
☒	☐	Estimates of effect sizes (e.g. Cohen's d, Pearson's r), indicating how they were calculated

Our web collection on statistics for biologists contains articles on many of the points above.

Software and code

Policy information about availability of computer code

Data collection	No specialist or commercial software was used
Data analysis	R code for running analyses is available as part of supplementary information (Supplementary Data 4)

For manuscripts utilizing custom algorithms or software that are central to the research but not yet described in published literature, software must be made available to editors and reviewers. We strongly encourage code deposition in a community repository (e.g. GitHub). See the Nature Portfolio guidelines for submitting code & software for further information.

Data

Policy information about availability of data

All manuscripts must include a data availability statement. This statement should provide the following information, where applicable:

- Accession codes, unique identifiers, or web links for publicly available datasets
- A description of any restrictions on data availability
- For clinical datasets or third party data, please ensure that the statement adheres to our policy

The data that support the findings of this study are available in Figshare and accessible at: https://doi.org/10.6084/m9.figshare.21622284
Supplementary Tables 1–5 include outputs from the following analyses: Poisson regressions of counts vs. groups; phylogenetic analyses of variance for the complexity indices; phylogenetically corrected correlations between various categories of vertebral counts and complexity indices; robust linear regressions between ancestral complexity values and descendant-ancestor differences, as well as between node ages and ancestral values; subclade tests. Supplementary Data

1 lists taxa, their presacral counts, and their complexity indices. It also reports univariate statistics and histogram distributions for the thoracic and lumbar counts alongside probability density distributions for various complexity indices. The literature sources on vertebral formulae are listed in Supplementary Data 2. The time-scaled phylogeny is available in Supplementary Data 3 as an object of class 'phylo'. R code is reproduced in Supplementary Data 4 and is accompanied by templates for running analyses on individual data files extracted from Supplementary Data 1. Such data files are combined as separate tabs within individual spreadsheetsand are available as Source Data for main Figures 3–6 and for Extended Data Figures 1–5.

Human research participants

Policy information about studies involving human research participants and Sex and Gender in Research.

Reporting on sex and gender	*Use the terms sex (biological attribute) and gender (shaped by social and cultural circumstances) carefully in order to avoid confusing both terms. Indicate if findings apply to only one sex or gender; describe whether sex and gender were considered in study design whether sex and/or gender was determined based on self-reporting or assigned and methods used. Provide in the source data disaggregated sex and gender data where this information has been collected, and consent has been obtained for sharing of individual-level data; provide overall numbers in this Reporting Summary. Please state if this information has not been collected. Report sex- and gender-based analyses where performed, justify reasons for lack of sex- and gender-based analysis.*
Population characteristics	*Describe the covariate-relevant population characteristics of the human research participants (e.g. age, genotypic information, past and current diagnosis and treatment categories). If you filled out the behavioural & social sciences study design questions and have nothing to add here, write "See above."*
Recruitment	*Describe how participants were recruited. Outline any potential self-selection bias or other biases that may be present and how these are likely to impact results.*
Ethics oversight	*Identify the organization(s) that approved the study protocol.*

Note that full information on the approval of the study protocol must also be provided in the manuscript.

Field-specific reporting

Please select the one below that is the best fit for your research. If you are not sure, read the appropriate sections before making your selection.

☐ Life sciences ☐ Behavioural & social sciences ☒ Ecological, evolutionary & environmental sciences

For a reference copy of the document with all sections, see nature.com/documents/nr-reporting-summary-flat.pdf

Ecological, evolutionary & environmental sciences study design

All studies must disclose on these points even when the disclosure is negative.

Study description	We analyze the evolution of complexity in serial structures, using the mammal vertebral column as a case-study. We derive indices of numerical abundance and proportional distributions of vertebral types and subject them to comparative phylogenetic methods, testing for occurrences of rate heterogeneities and evolutionary trends in complexity
Research sample	Vertebral data on 1136 mammal species
Sampling strategy	Taxa covered in this study belong to all major groups of extant mammals
Data collection	Online data collection of vertebral formulae from a representative sample of extant mammal species
Timing and spatial scale	Online data collection started approximately two years ago and was completed near the end of 2021
Data exclusions	None of the collected data were excluded from the analyses
Reproducibility	All results can be reproduced with codes made available as part of supplementary information (Supplementary Data 4)
Randomization	The nature of our study does not involve random assignments of samples to groups. Species are assigned to major mammal groups following current taxonomy
Blinding	All members of the team re-ran analyses to confirm results

Did the study involve field work? ☐ Yes ☒ No

Reporting for specific materials, systems and methods

We require information from authors about some types of materials, experimental systems and methods used in many studies. Here, indicate whether each material, system or method listed is relevant to your study. If you are not sure if a list item applies to your research, read the appropriate section before selecting a response.

Materials & experimental systems

n/a	Involved in the study
☒	☐ Antibodies
☒	☐ Eukaryotic cell lines
☒	☐ Palaeontology and archaeology
☒	☐ Animals and other organisms
☒	☐ Clinical data
☒	☐ Dual use research of concern

Methods

n/a	Involved in the study
☒	☐ ChIP-seq
☒	☐ Flow cytometry
☒	☐ MRI-based neuroimaging
