# Supplementary material for: Divergent vertebral formulae shape the evolution of axial complexity in mammals
Source: Nat Ecol Evol. 2023 Mar 6;7(3):367–81. doi: 10.1038/s41559-023-01982-5 (PMC9998275; doi:10.1038/s41559-023-01982-5)
Supplement: Supplementary file 6 — Time-calibrated phylogeny of 1,136 extant mammal species. The file is formatted as an object of class ‘phylo’ readable in R. It can be opened in WORDS and saved as a text file, after replacing the ‘.R’ suffix with ‘.nex’. [file 41559_2023_1982_MOESM6_ESM.pdf]

```
#NEXUS
[R-package APE, Wed Jul 14 13:20:46 2021]

BEGIN TAXA;
	DIMENSIONS NTAX = 1136;
	TAXLABELS
		Zaglossus_bruijnii
		Tachyglossus_aculeatus
		Ornithorhynchus_anatinus
		Solenodon_paradoxus
		Solenodon_cubanus
		Galemys_pyrenaicus
		Scapanus_latimanus
		Scalopus_aquaticus
		Talpa_europaea
		Euroscaptor_mizura
		Euroscaptor_micrura
		Mogera_tokudae
		Mogera_imaizumii
		Mogera_wogura
		Euroscaptor_subanura
		Condylura_cristata
		Hylomys_suillus
		Echinosorex_gymnura
		Erinaceus_europaeus
		Paraechinus_micropus
		Hemiechinus_auritus
		Atelerix_algirus
		Scutisorex_somereni
		Suncus_madagascariensis
		Crocidura_crenata
		Crocidura_canariensis
		Sorex_araneus
		Sorex_minutus
		Cryptotis_parva
		Blarina_hylophaga
		Notiosorex_crawfordi
		Smutsia_gigantea
		Phataginus_tricuspis
		Manis_pentadactyla
		Urocyon_cinereoargenteus
		Vulpes_lagopus
		Vulpes_zerda
		Vulpes_vulpes
		Otocyon_megalotis
		Nyctereutes_procyonoides
		Lycaon_pictus
		Chrysocyon_brachyurus
		Canis_mesomelas
		Canis_simensis
		Canis_latrans
		Canis_aureus
		Canis_lupus
		Cuon_alpinus
		Canis_adustus
		Pseudalopex_gymnocercus
		Speothos_venaticus
		Cerdocyon_thous
```

Neomonachus_tropicalis
Monachus_monachus
Mirounga_leonina
Mirounga_angustirostris
Lobodon_carcinophaga
Leptonychotes_weddellii
Ommatophoca_rossii
Hydrurga_leptonyx
Erignathus_barbatus
Pagophilus_groenlandicus
Phoca_vitulina
Pusa_hispida
Halichoerus_grypus
Cystophora_cristata
Odobenus_rosmarus
Callorhinus_ursinus
Zalophus_californianus
Zalophus_japonicus
Eumetopias_jubatus
Arctocephalus_pusillus
Neophoca_cinerea
Otaria_bryonia
Arctocephalus_tropicalis
Arctocephalus_forsteri
Arctocephalus_australis
Mydaus_javanensis
Spilogale_gracilis
Mephitis_mephitis
Conepatus_humboldtii
Conepatus_chinga
Potos_flavus
Procyon_lotor
Procyon_cancrivorus
Bassariscus_astutus
Nasua_nasua
Nasua_narica
Bassaricyon_alleni
Taxidea_taxus
Meles_meles
Meles_anakuma
Arctonyx_collaris
Mellivora_capensis
Gulo_gulo
Martes_flavigula
Martes_foina
Martes_zibellina
Martes_martes
Martes_americana
Martes_pennanti
Eira_barbara
Melogale_orientalis
Neovison_vison
Mustela_frenata
Mustela_felipei
Mustela_erminea
Mustela_nivalis
Mustela_sibirica
Mustela_nigripes

Mustela_lutreolina
Mustela_putorius
Ictonyx_libyca
Galictis_vittata
Lyncodon_patagonicus
Galictis_cuja
Pteronura_brasiliensis
Lontra_longicaudis
Lontra_provocax
Lontra_canadensis
Enhydra_lutris
Lutra_maculicollis
Lutra_lutra
Aonyx_cinerea
Lutrogale_perspicillata
Aonyx_capensis
Ailurus_fulgens
Ursus_maritimus
Ursus_arctos
Melursus_ursinus
Ursus_thibetanus
Ursus_americanus
Helarctos_malayanus
Ailuropoda_melanoleuca
Nandinia_binotata
Hyaena_hyaena
Hyaena_brunnea
Proteles_cristata
Crocuta_crocuta
Eupleres_goudotii
Salanoia_concolor
Galidictis_fasciata
Galidia_elegans
Cryptoprocta_ferox
Herpestes_vitticollis
Herpestes_javanicus
Herpestes_edwardsii
Herpestes_ichneumon
Ichneumia_albicauda
Cynictis_penicillata
Bdeogale_crassicauda
Crossarchus_obscurus
Helogale_parvula
Liberiictis_kuhni
Mungos_mungo
Suricata_suricatta
Genetta_maculata
Genetta_tigrina
Genetta_genetta
Genetta_servalina
Viverricula_indica
Viverra_tangalunga
Viverra_zibetha
Civettictis_civetta
Macrogalidia_musschenbroekii
Cynogale_bennettii
Hemigalus_derbyanus
Paradoxurus_hermaphroditus

Arctictis_binturong
Prionodon_pardicolor
Prionodon_linsang
Neofelis_nebulosa
Panthera_tigris
Panthera_onca
Panthera_uncia
Panthera_pardus
Panthera_leo
Lynx_rufus
Lynx_lynx
Lynx_canadensis
Acinonyx_jubatus
Puma_concolor
Felis_catus
Felis_silvestris
Leopardus_pardalis
Leopardus_wiedii
Leopardus_tigrinus
Prionailurus_bengalensis
Leptailurus_serval
Caracal_caracal
Equus_africanus
Equus_grevyi
Equus_quagga
Equus_zebra
Equus_hemionus
Equus_caballus
Equus_ferus
Tapirus_indicus
Tapirus_terrestris
Tapirus_bairdii
Rhinoceros_unicornis
Rhinoceros_sondaicus
Dicerorhinus_sumatrensis
Diceros_bicornis
Ceratotherium_simum
Vicugna_pacos
Vicugna_vicugna
Lama_glama
Camelus_dromedarius
Camelus_ferus
Tayassu_pecari
Pecari_tajacu
Sus_scrofa
Sus_barbatus
Potamochoerus_porcus
Phacochoerus_africanus
Phacochoerus_aethiopicus
Hylochoerus_meinertzhageni
Babyrousa_babyrussa
Hippopotamus_amphibius
Choeropsis_liberiensis
Indopacetus_pacificus
Kogia_sima
Kogia_breviceps
Physeter_macrocephalus
Platanista_gangetica

Pontoporia_blainvillei
Inia_geoffrensis
Inia_araguaiaensis
Lipotes_vexillifer
Delphinapterus_leucas
Mesoplodon_bowdoini
Monodon_monoceros
Neophocaena_phocaenoides
Phocoenoides_dalli
Phocoena_phocoena
Phocoena_spinipinnis
Phocoena_dioptrica
Lagenorhynchus_acutus
Lagenorhynchus_albirostris
Orcinus_orca
Orcaella_brevirostris
Grampus_griseus
Pseudorca_crassidens
Peponocephala_electra
Globicephala_melas
Globicephala_macrorhynchus
Feresa_attenuata
Steno_bredanensis
Sotalia_guianensis
Sotalia_fluviatilis
Tursiops_truncatus
Stenella_attenuata
Lagenodelphis_hosei
Stenella_longirostris
Sousa_chinensis
Tursiops_aduncus
Stenella_frontalis
Stenella_coeruleoalba
Stenella_clymene
Delphinus_delphis
Delphinus_capensis
Lagenorhynchus_obscurus
Lagenorhynchus_obliquidens
Lagenorhynchus_cruciger
Lagenorhynchus_australis
Cephalorhynchus_hectori
Cephalorhynchus_heavisidii
Cephalorhynchus_eutropia
Cephalorhynchus_commersonii
Berardius_bairdii
Berardius_arnuxii
Mesoplodon_bidens
Ziphius_cavirostris
Mesoplodon_stejnegeri
Hyperoodon_planifrons
Hyperoodon_ampullatus
Mesoplodon_hectori
Mesoplodon_carlhubbsi
Mesoplodon_layardii
Mesoplodon_mirus
Mesoplodon_europaeus
Mesoplodon_densirostris
Mesoplodon_grayi

Tasmacetus_shepherdi
Eubalaena_glacialis
Eubalaena_japonica
Balaenoptera_physalus
Balaenoptera_musculus
Balaenoptera_omurai
Balaenoptera_borealis
Balaenoptera_edeni
Mesoplodon_perrini
Balaenoptera_bonaerensis
Mesoplodon_peruvianus
Lissodelphis_peronii
Lissodelphis_borealis
Balaena_mysticetus
Caperea_marginata
Balaenoptera_acutorostrata
Mesoplodon_ginkgodens
Megaptera_novaeangliae
Eschrichtius_robustus
Moschiola_meminna
Moschiola_indica
Tragulus_javanicus
Tragulus_kanchil
Tragulus_napu
Okapia_johnstoni
Giraffa_camelopardalis
Antilocapra_americana
Muntiacus_muntjak
Dama_dama
Elaphurus_davidianus
Rusa_unicolor
Cervus_elaphus
Axis_calamianensis
Axis_axis
Rangifer_tarandus
Odocoileus_virginianus
Odocoileus_hemionus
Mazama_gouazoubira
Blastocerus_dichotomus
Pudu_puda
Hydropotes_inermis
Capreolus_capreolus
Alces_alces
Moschus_moschiferus
Moschus_berezovskii
Boselaphus_tragocamelus
Tetracerus_quadricornis
Tragelaphus_imberbis
Tragelaphus_angasii
Tragelaphus_scriptus
Tragelaphus_eurycerus
Tragelaphus_spekii
Tragelaphus_buxtoni
Tragelaphus_strepsiceros
Syncerus_caffer
Bubalus_mindorensis
Bubalus_depressicornis
Bubalus_arnee

Bos_gaurus
Bos_taurus
Bos_indicus
Bison_bonasus
Bos_mutus
Bos_grunniens
Bison_bison
Nesotragus_moschatus
Aepyceros_melampus
Pelea_capreolus
Redunca_fulvorufula
Redunca_redunca
Redunca_arundinum
Kobus_megaceros
Kobus_leche
Kobus_vardonii
Kobus_kob
Kobus_ellipsiprymnus
Ourebia_ourebi
Raphicerus_melanotis
Raphicerus_sharpei
Raphicerus_campestris
Madoqua_kirkii
Madoqua_guentheri
Saiga_tatarica
Nanger_granti
Eudorcas_rufifrons
Gazella_dorcas
Gazella_gazella
Gazella_spekei
Antilope_cervicapra
Litocranius_walleri
Antidorcas_marsupialis
Neotragus_pygmaeus
Oreotragus_oreotragus
Philantomba_monticola
Sylvicapra_grimmia
Cephalophus_silvicultor
Cephalophus_dorsalis
Cephalophus_leucogaster
Cephalophus_nigrifrons
Cephalophus_natalensis
Cephalophus_weynsi
Ovibos_moschatus
Capricornis_crispus
Ovis_canadensis
Ovis_orientalis
Ovis_aries
Ovis_ammon
Capra_ibex
Capra_hircus
Pseudois_nayaur
Myotragus_balearicus
Oreamnos_americanus
Rupicapra_rupicapra
Ammotragus_lervia
Connochaetes_taurinus
Connochaetes_gnou

Damaliscus_pygargus
Damaliscus_lunatus
Beatragus_hunteri
Alcelaphus_buselaphus
Hippotragus_niger
Hippotragus_equinus
Oryx_dammah
Oryx_leucoryx
Oryx_gazella
Oryx_beisa
Rhinopoma_hardwickii
Rhinopoma_microphyllum
Lavia_frons
Megaderma_spasma
Rhinolophus_hipposideros
Asellia_tridens
Hipposideros_caffer
Hipposideros_diadema
Cynopterus_titthaecheilus
Cynopterus_brachyotis
Epomops_franqueti
Hypsignathus_monstrosus
Rousettus_aegyptiacus
Eidolon_helvum
Pteropus_giganteus
Pteropus_rufus
Pteropus_vampyrus
Macroglossus_minimus
Myzopoda_aurita
Mystacina_tuberculata
Thyroptera_discifera
Noctilio_leporinus
Pteronotus_parnellii
Mormoops_megalophylla
Micronycteris_microtis
Anoura_caudifer
Glossophaga_soricina
Glossophaga_morenoi
Phylloderma_stenops
Phyllostomus_discolor
Lonchorhina_inusitata
Chrotopterus_auritus
Vampyrum_spectrum
Lonchophylla_inexpectata
Carollia_perspicillata
Artibeus_jamaicensis
Artibeus_planirostris
Pygoderma_bilabiatum
Vampyressa_pusilla
Sturnira_erythromos
Sturnira_aratathomasi
Rhinophylla_pumilio
Lonchorhina_orinocensis
Lonchorhina_fernandezi
Lonchorhina_marinkellei
Lonchorhina_aurita
Diphylla_ecaudata
Desmodus_rotundus

Diaemus_youngi
Macrotus_waterhousii
Macrotus_californicus
Nyctinomops_macrotis
Molossus_molossus
Promops_nasutus
Molossops_temminckii
Cynomops_planirostris
Eumops_perotis
Tadarida_brasiliensis
Molossus_currentium
Myotis_daubentonii
Myotis_keaysi
Myotis_velifer
Myotis_chiloensis
Myotis_lucifugus
Myotis_evotis
Eptesicus_nilssonii
Eptesicus_serotinus
Histiotus_montanus
Histiotus_magellanicus
Eptesicus_diminutus
Lasionycteris_noctivagans
Pipistrellus_pipistrellus
Nyctalus_noctula
Vespertilio_murinus
Chalinolobus_tuberculatus
Neoromicia_nana
Lasiurus_cinereus
Lasiurus_ega
Antrozous_pallidus
Otonycteris_hemprichii
Plecotus_auritus
Euderma_maculatum
Barbastella_barbastellus
Miniopterus_schreibersii
Nycteris_javanica
Nycteris_thebaica
Saccopteryx_bilineata
Peropteryx_leucoptera
Balantiopteryx_plicata
Galeopterus_variegatus
Cynocephalus_volans
Nycticebus_coucang
Loris_tardigradus
Otolemur_garnettii
Otolemur_crassicaudatus
Galago_senegalensis
Euoticus_elegantulus
Perodicticus_potto
Arctocebus_calabarensis
Daubentonia_madagascariensis
Indri_indri
Propithecus_diadema
Propithecus_verreauxi
Propithecus_coquereli
Avahi_laniger
Varecia_variegata

Lemur_catta
Hapalemur_griseus
Eulemur_macaco
Eulemur_rubriventer
Eulemur_mongoz
Eulemur_collaris
Eulemur_fulvus
Eulemur_sanfordi
Eulemur_albifrons
Lepilemur_mustelinus
Cheirogaleus_medius
Microcebus_murinus
Microcebus_rufus
Tarsius_syrichta
Pithecia_irrorata
Pithecia_monachus
Pithecia_pithecia
Cacajao_melanocephalus
Callicebus_moloch
Leontocebus_nigricollis
Saguinus_oedipus
Leontopithecus_rosalia
Mico_humeralifer
Callithrix_jacchus
Cebuella_pygmaea
Saimiri_sciureus
Cebus_olivaceus
Cebus_albifrons
Cebus_capucinus
Aotus_azarae
Aotus_nancymaae
Alouatta_seniculus
Alouatta_caraya
Alouatta_palliata
Alouatta_pigra
Lagothrix_lagotricha
Brachyteles_hypoxanthus
Brachyteles_arachnoides
Ateles_geoffroyi
Ateles_fusciceps
Ateles_paniscus
Ateles_chamek
Ateles_belzebuth
Symphalangus_syndactylus
Hylobates_pileatus
Hylobates_lar
Hylobates_muelleri
Hylobates_moloch
Hylobates_klossii
Hylobates_agilis
Nomascus_concolor
Nomascus_gabriellae
Nomascus_leucogenys
Hoolock_hoolock
Pongo_pygmaeus
Pongo_abelii
Pan_troglodytes
Pan_paniscus

Homo_sapiens
Gorilla_gorilla
Gorilla_beringei
Semnopithecus_schistaceus
Trachypithecus_cristatus
Trachypithecus_phayrei
Semnopithecus_entellus
Presbytis_rubicunda
Presbytis_melalophos
Rhinopithecus_bieti
Rhinopithecus_roxellana
Nasalis_larvatus
Colobus_guereza
Colobus_vellerosus
Macaca_leonina
Macaca_sinica
Macaca_silenus
Macaca_nemestrina
Macaca_maura
Macaca_radiata
Macaca_fascicularis
Macaca_arctoides
Macaca_nigra
Macaca_fuscata
Macaca_mulatta
Macaca_sylvanus
Theropithecus_gelada
Papio_cynocephalus
Papio_papio
Papio_hamadryas
Papio_anubis
Lophocebus_aterrimus
Lophocebus_albigena
Cercocebus_atys
Mandrillus_sphinx
Mandrillus_leucophaeus
Cercopithecus_mitis
Cercopithecus_ascanius
Erythrocebus_patas
Chlorocebus_pygerythrus
Chlorocebus_aethiops
Chlorocebus_cynosuros
Chlorocebus_sabaeus
Miopithecus_talapoin
Ptilocercus_lowii
Tupaia_glis
Tupaia_nicobarica
Urogale_everetti
Tupaia_tana
Dendrogale_melanura
Anathana_ellioti
Sylvilagus_audubonii
Oryctolagus_cuniculus
Pentalagus_furnessi
Lepus_californicus
Lepus_alleni
Lepus_townsendii
Lepus_timidus

Lepus_europaeus
Lepus_americanus
Nesolagus_timminsi
Nesolagus_netscheri
Ochotona_hyperborea
Ochotona_princeps
Muscardinus_avellanarius
Aplodontia_rufa
Ratufa_indica
Callosciurus_erythraeus
Tamias_striatus
Tamias_minimus
Spermophilus_dauricus
Ictidomys_tridecemlineatus
Cynomys_ludovicianus
Marmota_monax
Marmota_caligata
Marmota_marmota
Marmota_sibirica
Sciurus_anomalus
Sciurus_niger
Sciurus_carolinensis
Sciurus_griseus
Microsciurus_mimulus
Microsciurus_alfari
Microsciurus_flaviventer
Sciurus_vulgaris
Tamiasciurus_hudsonicus
Glaucomys_volans
Petaurista_leucogenys
Petaurista_philippensis
Petaurista_alborufus
Petaurista_grandis
Laonastes_aenigmamus
Atherurus_macrourus
Hystrix_javanica
Hystrix_cristata
Thryonomys_swinderianus
Heterocephalus_glaber
Heliophobius_argenteocinereus
Cryptomys_hottentotus
Georychus_capensis
Bathyergus_suillus
Cuniculus_paca
Myoprocta_acouchy
Dasyprocta_leporina
Dasyprocta_azarae
Hydrochoerus_hydrochaeris
Dolichotis_patagonum
Cavia_porcellus
Cavia_aperea
Erethizon_dorsatum
Coendou_prehensilis
Lagostomus_maximus
Chinchilla_lanigera
Chinchilla_chinchilla
Phyllomys_sulinus
Myocastor_coypus

Capromys_pilorides
Ctenomys_boliviensis
Ctenomys_talarum
Octodon_degus
Spalacopus_cyanus
Dipus_sagitta
Jaculus_orientalis
Jaculus_jaculus
Stylodipus_telum
Allactaga_euphratica
Allactaga_major
Zapus_princeps
Sicista_concolor
Spalax_leucodon
Rhizomys_pruinosus
Dicrostonyx_groenlandicus
Ondatra_zibethicus
Lemmus_sibiricus
Synaptomys_cooperi
Ellobius_talpinus
Microtus_arvalis
Microtus_pennsylvanicus
Microtus_ochrogaster
Microtus_chrotorrhinus
Microtus_oeconomus
Myodes_gapperi
Mesocricetus_auratus
Tscherskia_triton
Cricetus_cricetus
Cricetulus_migratorius
Cricetulus_longicaudatus
Cricetulus_barabensis
Phodopus_sungorus
Neotoma_fuscipes
Neotoma_lepida
Neotoma_floridana
Neotoma_albigula
Neotoma_mexicana
Neotoma_cinerea
Hodomys_alleni
Nelsonia_neotomodon
Xenomys_nelsoni
Ochrotomys_nuttalli
Reithrodontomys_creper
Reithrodontomys_mexicanus
Reithrodontomys_fulvescens
Reithrodontomys_sumichrasti
Reithrodontomys_megalotis
Reithrodontomys_humulis
Reithrodontomys_montanus
Isthmomys_pirrensis
Isthmomys_flavidus
Onychomys_torridus
Onychomys_leucogaster
Peromyscus_maniculatus
Peromyscus_leucopus
Megadontomys_thomasi
Peromyscus_yucatanicus

Peromyscus_guatemalensis
Peromyscus_mexicanus
Peromyscus_megalops
Peromyscus_melanophrys
Habromys_lepturus
Habromys_lophurus
Peromyscus_crinitus
Peromyscus_ochraventer
Peromyscus_truei
Peromyscus_difficilis
Peromyscus_attwateri
Peromyscus_furvus
Peromyscus_spicilegus
Peromyscus_boylii
Podomys_floridanus
Neotomodon_alstoni
Peromyscus_californicus
Peromyscus_eremicus
Osgoodomys_banderanus
Scotinomys_xerampelinus
Scotinomys_teguina
Baiomys_taylori
Baiomys_musculus
Tylomys_panamensis
Tylomys_mirae
Tylomys_watsoni
Tylomys_fulviventer
Tylomys_nudicaudus
Ototylomys_phyllotis
Nyctomys_sumichrasti
Chibchanomys_trichotis
Reithrodon_auritus
Rheomys_underwoodi
Rheomys_thomasi
Rheomys_mexicanus
Rheomys_raptor
Ichthyomys_pittieri
Ichthyomys_hydrobates
Ichthyomys_tweedii
Neusticomys_venezuelae
Neusticomys_ferreirai
Neusticomys_monticolus
Anotomys_leander
Chinchillula_sahamae
Sigmodon_leucotis
Sigmodon_fulviventer
Sigmodon_alleni
Sigmodon_ochrognathus
Sigmodon_hispidus
Rhipidomys_macconnelli
Rhipidomys_nitela
Rhipidomys_mastacalis
Rhipidomys_fulviventer
Rhipidomys_venustus
Rhipidomys_couesi
Rhipidomys_venezuelae
Rhipidomys_latimanus
Rhipidomys_austrinus

Aepeomys_lugens
Thomasomys_gracilis
Thomasomys_cinereus
Thomasomys_rhoadsi
Thomasomys_baeops
Thomasomys_aureus
Thomasomys_pyrrhonotus
Thomasomys_daphne
Thomasomys_oreas
Thomasomys_hylophilus
Thomasomys_paramorum
Chilomys_instans
Akodon_bogotensis
Blarinomys_breviceps
Scapteromys_tumidus
Scapteromys_meridionalis
Scapteromys_aquaticus
Kunsia_tomentosus
Thalpomys_lasiotis
Necromys_amoenus
Necromys_urichi
Necromys_lasiurus
Necromys_temchuki
Necromys_obscurus
Necromys_lactens
Akodon_mimus
Akodon_azarae
Akodon_boliviensis
Akodon_subfuscus
Akodon_lutescens
Akodon_cursor
Akodon_simulator
Akodon_albiventer
Akodon_mollis
Akodon_torques
Akodon_aerosus
Deltamys_kempi
Thaptomys_nigrita
Oxymycterus_delator
Oxymycterus_paramensis
Oxymycterus_rufus
Oxymycterus_inca
Zygodontomys_brevicauda
Oecomys_concolor
Oecomys_roberti
Oecomys_bicolor
Oecomys_superans
Oecomys_trinitatis
Oecomys_mamorae
Oecomys_paricola
Transandinomys_talamancae
Transandinomys_bolivaris
Euryoryzomys_nitidus
Euryoryzomys_russatus
Euryoryzomys_legatus
Nephelomys_albigularis
Nephelomys_keaysi
Hylaeamys_megacephalus

Handleyomys_alfaroi
Handleyomys_melanotis
Handleyomys_chapmani
Neacomys_guianae
Neacomys_spinosus
Neacomys_tenuipes
Microryzomys_minutus
Microryzomys_altissimus
Oligoryzomys_microtis
Oligoryzomys_nigripes
Oligoryzomys_destructor
Oligoryzomys_fulvescens
Oligoryzomys_eliurus
Oligoryzomys_delticola
Oligoryzomys_chacoensis
Oligoryzomys_andinus
Oligoryzomys_magellanicus
Oligoryzomys_longicaudatus
Oligoryzomys_flavescens
Eremoryzomys_polius
Cerradomys_subflavus
Sooretamys_angouya
Holochilus_brasiliensis
Holochilus_chacarius
Pseudoryzomys_simplex
Lundomys_molitor
Nectomys_squamipes
Oryzomys_couesi
Oryzomys_palustris
Sigmodontomys_alfari
Melanomys_caliginosus
Nesoryzomys_indefessus
Irenomys_tarsalis
Euneomys_petersoni
Euneomys_chinchilloides
Andinomys_edax
Punomys_kofordi
Delomys_sublineatus
Delomys_dorsalis
Calomys_lepidus
Calomys_sorellus
Calomys_musculinus
Calomys_laucha
Calomys_tener
Calomys_boliviae
Calomys_callosus
Calomys_venustus
Calomys_hummelincki
Eligmodontia_morgani
Eligmodontia_puerulus
Eligmodontia_moreni
Eligmodontia_hirtipes
Eligmodontia_bolsonensis
Graomys_griseoflavus
Graomys_domorum
Andalgalomys_pearsoni
Andalgalomys_olrogi
Salinomys_delicatus

Phyllotis_wolffsohni
Tapecomys_primus
Auliscomys_sublimis
Auliscomys_pictus
Auliscomys_boliviensis
Galenomys_garleppi
Loxodontomys_micropus
Phyllotis_osilae
Phyllotis_alisosiensis
Phyllotis_anitae
Phyllotis_caprinus
Phyllotis_xanthopygus
Phyllotis_darwini
Phyllotis_amicus
Paralomys_gerbillus
Phyllotis_haggardi
Phyllotis_andium
Calassomys_apicalis
Akodon_neocenus
Wiedomys_pyrrhorhinos
Juliomys_pictipes
Abrothrix_olivaceus
Abrothrix_andinus
Abrothrix_jelskii
Abrothrix_illuteus
Abrothrix_longipilis
Abrothrix_sanborni
Notiomys_edwardsii
Geoxus_valdivianus
Chelemys_macronyx
Mystromys_albicaudatus
Saccostomus_campestris
Beamys_hindei
Cricetomys_gambianus
Brachytarsomys_albicauda
Gymnuromys_roberti
Eliurus_myoxinus
Brachyuromys_ramirohitra
Nesomys_rufus
Nesomys_audeberti
Macrotarsomys_bastardi
Calomyscus_baluchi
Lophiomys_imhausi
Pachyuromys_duprasi
Psammomys_obesus
Meriones_shawi
Meriones_unguiculatus
Sekeetamys_calurus
Ammodillus_imbellis
Microdillus_peeli
Gerbillus_cheesmani
Taterillus_arenarius
Gerbilliscus_nigricaudus
Gerbillurus_setzeri
Desmodillus_auricularis
Tatera_indica
Notomys_amplus
Conilurus_albipes

Hydromys_chrysogaster
Mastomys_coucha
Mus_musculus
Rhabdomys_pumilio
Micromys_minutus
Maxomys_inflatus
Rattus_rattus
Rattus_fuscipes
Rattus_norvegicus
Pedetes_surdaster
Pedetes_capensis
Anomalurus_pelii
Anomalurus_derbianus
Anomalurus_beecrofti
Anomalurus_pusillus
Microdipodops_pallidus
Dipodomys_spectabilis
Dipodomys_ordii
Dipodomys_heermanni
Dipodomys_merriami
Thomomys_bulbivorus
Thomomys_umbrinus
Thomomys_bottae
Geomys_pinetis
Geomys_bursarius
Geomys_arenarius
Cratogeomys_castanops
Liomys_salvini
Heteromys_anomalus
Perognathus_fasciatus
Castor_fiber
Castor_canadensis
Hydrodamalis_gigas
Trichechus_manatus
Trichechus_senegalensis
Trichechus_inunguis
Dugong_dugon
Loxodonta_africana
Elephas_maximus
Procavia_capensis
Heterohyrax_brucei
Dendrohyrax_validus
Dendrohyrax_dorsalis
Dendrohyrax_arboreus
Orycteropus_afer
Rhynchocyon_cirnei
Rhynchocyon_chrysopygus
Rhynchocyon_petersi
Elephantulus_myurus
Elephantulus_edwardii
Macroscelides_proboscideus
Petrodromus_tetradactylus
Elephantulus_rufescens
Elephantulus_brachyrhynchus
Elephantulus_intufi
Elephantulus_rupestris
Potamogale_velox
Limnogale_mergulus

Microgale_talazaci
Microgale_longicaudata
Microgale_cowani
Oryzorictes_tetradactylus
Tenrec_ecaudatus
Hemicentetes_semispinosus
Setifer_setosus
Echinops_telfairi
Eremitalpa_granti
Amblysomus_hottentotus
Calcochloris_obtusirostris
Chrysospalax_trevelyani
Chrysochloris_asiatica
Dasypus_hybridus
Dasypus_novemcinctus
Dasypus_kappleri
Dasypus_septemcinctus
Zaedyus_pichiy
Euphractus_sexcinctus
Chaetophractus_vellerosus
Chaetophractus_villosus
Chlamyphorus_truncatus
Tolypeutes_matacus
Priodontes_maximus
Cabassous_unicinctus
Tamandua_tetradactyla
Tamandua_mexicana
Myrmecophaga_tridactyla
Cyclopes_didactylus
Choloepus_hoffmanni
Choloepus_didactylus
Bradypus_tridactylus
Bradypus_variegatus
Rhyncholestes_raphanurus
Caenolestes_fuliginosus
Glironia_venusta
Monodelphis_domestica
Gracilinanus_microtarsus
Metachirus_nudicaudatus
Didelphis_virginiana
Didelphis_marsupialis
Didelphis_albiventris
Chironectes_minimus
Caluromys_philander
Dromiciops_gliroides
Notoryctes_typhlops
Notoryctes_caurinus
Macrotis_lagotis
Isoodon_macrourus
Isoodon_obesulus
Perameles_gunnii
Perameles_nasuta
Sarcophilus_harrisii
Dasyurus_maculatus
Dasyurus_viverrinus
Dasyuroides_byrnei
Dasycercus_cristicauda
Antechinus_flavipes

```
            Thylacinus_cynocephalus
            Myrmecobius_fasciatus
            Phascolarctos_cinereus
            Vombatus_ursinus
            Lasiorhinus_latifrons
            Cercartetus_concinnus
            Trichosurus_vulpecula
            Spilocuscus_maculatus
            Phalanger_sericeus
            Phalanger_orientalis
            Hypsiprymnodon_moschatus
            Thylogale_billardierii
            Petrogale_lateralis
            Petrogale_penicillata
            Dendrolagus_goodfellowi
            Dendrolagus_matschiei
            Dendrolagus_bennettianus
            Dendrolagus_lumholtzi
            Macropus_fuliginosus
            Macropus_giganteus
            Macropus_antilopinus
            Macropus_robustus
            Macropus_rufus
            Macropus_rufogriseus
            Macropus_eugenii
            Macropus_agilis
            Macropus_parryi
            Macropus_parma
            Macropus_irma
            Dendrolagus_ursinus
            Potorous_longipes
            Potorous_tridactylus
            Bettongia_penicillata
            Bettongia_gaimardi
            Aepyprymnus_rufescens
            Pseudocheirus_peregrinus
            Petauroides_volans
            Petaurus_australis
            Petaurus_norfolcensis
            Petaurus_breviceps
      ;
END;
BEGIN TREES;
      TRANSLATE
            1     Zaglossus_bruijnii,
            2     Tachyglossus_aculeatus,
            3     Ornithorhynchus_anatinus,
            4     Solenodon_paradoxus,
            5     Solenodon_cubanus,
            6     Galemys_pyrenaicus,
            7     Scapanus_latimanus,
            8     Scalopus_aquaticus,
            9     Talpa_europaea,
            10    Euroscaptor_mizura,
            11    Euroscaptor_micrura,
            12    Mogera_tokudae,
            13    Mogera_imaizumii,
            14    Mogera_wogura,
```

15 Euroscaptor_subanura,
16 Condylura_cristata,
17 Hylomys_suillus,
18 Echinosorex_gymnura,
19 Erinaceus_europaeus,
20 Paraechinus_micropus,
21 Hemiechinus_auritus,
22 Atelerix_algirus,
23 Scutisorex_somereni,
24 Suncus_madagascariensis,
25 Crocidura_crenata,
26 Crocidura_canariensis,
27 Sorex_araneus,
28 Sorex_minutus,
29 Cryptotis_parva,
30 Blarina_hylophaga,
31 Notiosorex_crawfordi,
32 Smutsia_gigantea,
33 Phataginus_tricuspis,
34 Manis_pentadactyla,
35 Urocyon_cinereoargenteus,
36 Vulpes_lagopus,
37 Vulpes_zerda,
38 Vulpes_vulpes,
39 Otocyon_megalotis,
40 Nyctereutes_procyonoides,
41 Lycaon_pictus,
42 Chrysocyon_brachyurus,
43 Canis_mesomelas,
44 Canis_simensis,
45 Canis_latrans,
46 Canis_aureus,
47 Canis_lupus,
48 Cuon_alpinus,
49 Canis_adustus,
50 Pseudalopex_gymnocercus,
51 Speothos_venaticus,
52 Cerdocyon_thous,
53 Neomonachus_tropicalis,
54 Monachus_monachus,
55 Mirounga_leonina,
56 Mirounga_angustirostris,
57 Lobodon_carcinophaga,
58 Leptonychotes_weddellii,
59 Ommatophoca_rossii,
60 Hydrurga_leptonyx,
61 Erignathus_barbatus,
62 Pagophilus_groenlandicus,
63 Phoca_vitulina,
64 Pusa_hispida,
65 Halichoerus_grypus,
66 Cystophora_cristata,
67 Odobenus_rosmarus,
68 Callorhinus_ursinus,
69 Zalophus_californianus,
70 Zalophus_japonicus,
71 Eumetopias_jubatus,
72 Arctocephalus_pusillus,

73 Neophoca_cinerea,
74 Otaria_bryonia,
75 Arctocephalus_tropicalis,
76 Arctocephalus_forsteri,
77 Arctocephalus_australis,
78 Mydaus_javanensis,
79 Spilogale_gracilis,
80 Mephitis_mephitis,
81 Conepatus_humboldtii,
82 Conepatus_chinga,
83 Potos_flavus,
84 Procyon_lotor,
85 Procyon_cancrivorus,
86 Bassariscus_astutus,
87 Nasua_nasua,
88 Nasua_narica,
89 Bassaricyon_alleni,
90 Taxidea_taxus,
91 Meles_meles,
92 Meles_anakuma,
93 Arctonyx_collaris,
94 Mellivora_capensis,
95 Gulo_gulo,
96 Martes_flavigula,
97 Martes_foina,
98 Martes_zibellina,
99 Martes_martes,
100 Martes_americana,
101 Martes_pennanti,
102 Eira_barbara,
103 Melogale_orientalis,
104 Neovison_vison,
105 Mustela_frenata,
106 Mustela_felipei,
107 Mustela_erminea,
108 Mustela_nivalis,
109 Mustela_sibirica,
110 Mustela_nigripes,
111 Mustela_lutreolina,
112 Mustela_putorius,
113 Ictonyx_libyca,
114 Galictis_vittata,
115 Lyncodon_patagonicus,
116 Galictis_cuja,
117 Pteronura_brasiliensis,
118 Lontra_longicaudis,
119 Lontra_provocax,
120 Lontra_canadensis,
121 Enhydra_lutris,
122 Lutra_maculicollis,
123 Lutra_lutra,
124 Aonyx_cinerea,
125 Lutrogale_perspicillata,
126 Aonyx_capensis,
127 Ailurus_fulgens,
128 Ursus_maritimus,
129 Ursus_arctos,
130 Melursus_ursinus,

131 Ursus_thibetanus,
132 Ursus_americanus,
133 Helarctos_malayanus,
134 Ailuropoda_melanoleuca,
135 Nandinia_binotata,
136 Hyaena_hyaena,
137 Hyaena_brunnea,
138 Proteles_cristata,
139 Crocuta_crocuta,
140 Eupleres_goudotii,
141 Salanoia_concolor,
142 Galidictis_fasciata,
143 Galidia_elegans,
144 Cryptoprocta_ferox,
145 Herpestes_vitticollis,
146 Herpestes_javanicus,
147 Herpestes_edwardsii,
148 Herpestes_ichneumon,
149 Ichneumia_albicauda,
150 Cynictis_penicillata,
151 Bdeogale_crassicauda,
152 Crossarchus_obscurus,
153 Helogale_parvula,
154 Liberiictis_kuhni,
155 Mungos_mungo,
156 Suricata_suricatta,
157 Genetta_maculata,
158 Genetta_tigrina,
159 Genetta_genetta,
160 Genetta_servalina,
161 Viverricula_indica,
162 Viverra_tangalunga,
163 Viverra_zibetha,
164 Civettictis_civetta,
165 Macrogalidia_musschenbroekii,
166 Cynogale_bennettii,
167 Hemigalus_derbyanus,
168 Paradoxurus_hermaphroditus,
169 Arctictis_binturong,
170 Prionodon_pardicolor,
171 Prionodon_linsang,
172 Neofelis_nebulosa,
173 Panthera_tigris,
174 Panthera_onca,
175 Panthera_uncia,
176 Panthera_pardus,
177 Panthera_leo,
178 Lynx_rufus,
179 Lynx_lynx,
180 Lynx_canadensis,
181 Acinonyx_jubatus,
182 Puma_concolor,
183 Felis_catus,
184 Felis_silvestris,
185 Leopardus_pardalis,
186 Leopardus_wiedii,
187 Leopardus_tigrinus,
188 Prionailurus_bengalensis,

189 Leptailurus_serval,
190 Caracal_caracal,
191 Equus_africanus,
192 Equus_grevyi,
193 Equus_quagga,
194 Equus_zebra,
195 Equus_hemionus,
196 Equus_caballus,
197 Equus_ferus,
198 Tapirus_indicus,
199 Tapirus_terrestris,
200 Tapirus_bairdii,
201 Rhinoceros_unicornis,
202 Rhinoceros_sondaicus,
203 Dicerorhinus_sumatrensis,
204 Diceros_bicornis,
205 Ceratotherium_simum,
206 Vicugna_pacos,
207 Vicugna_vicugna,
208 Lama_glama,
209 Camelus_dromedarius,
210 Camelus_ferus,
211 Tayassu_pecari,
212 Pecari_tajacu,
213 Sus_scrofa,
214 Sus_barbatus,
215 Potamochoerus_porcus,
216 Phacochoerus_africanus,
217 Phacochoerus_aethiopicus,
218 Hylochoerus_meinertzhageni,
219 Babyrousa_babyrussa,
220 Hippopotamus_amphibius,
221 Choeropsis_liberiensis,
222 Indopacetus_pacificus,
223 Kogia_sima,
224 Kogia_breviceps,
225 Physeter_macrocephalus,
226 Platanista_gangetica,
227 Pontoporia_blainvillei,
228 Inia_geoffrensis,
229 Inia_araguaiaensis,
230 Lipotes_vexillifer,
231 Delphinapterus_leucas,
232 Mesoplodon_bowdoini,
233 Monodon_monoceros,
234 Neophocaena_phocaenoides,
235 Phocoenoides_dalli,
236 Phocoena_phocoena,
237 Phocoena_spinipinnis,
238 Phocoena_dioptrica,
239 Lagenorhynchus_acutus,
240 Lagenorhynchus_albirostris,
241 Orcinus_orca,
242 Orcaella_brevirostris,
243 Grampus_griseus,
244 Pseudorca_crassidens,
245 Peponocephala_electra,
246 Globicephala_melas,

```
Globicephala_macrorhynchus,
Feresa_attenuata,
Steno_bredanensis,
Sotalia_guianensis,
Sotalia_fluviatilis,
Tursiops_truncatus,
Stenella_attenuata,
Lagenodelphis_hosei,
Stenella_longirostris,
Sousa_chinensis,
Tursiops_aduncus,
Stenella_frontalis,
Stenella_coeruleoalba,
Stenella_clymene,
Delphinus_delphis,
Delphinus_capensis,
Lagenorhynchus_obscurus,
Lagenorhynchus_obliquidens,
Lagenorhynchus_cruciger,
Lagenorhynchus_australis,
Cephalorhynchus_hectori,
Cephalorhynchus_heavisidii,
Cephalorhynchus_eutropia,
Cephalorhynchus_commersonii,
Berardius_bairdii,
Berardius_arnuxii,
Mesoplodon_bidens,
Ziphius_cavirostris,
Mesoplodon_stejnegeri,
Hyperoodon_planifrons,
Hyperoodon_ampullatus,
Mesoplodon_hectori,
Mesoplodon_carlhubbsi,
Mesoplodon_layardii,
Mesoplodon_mirus,
Mesoplodon_europaeus,
Mesoplodon_densirostris,
Mesoplodon_grayi,
Tasmacetus_shepherdi,
Eubalaena_glacialis,
Eubalaena_japonica,
Balaenoptera_physalus,
Balaenoptera_musculus,
Balaenoptera_omurai,
Balaenoptera_borealis,
Balaenoptera_edeni,
Mesoplodon_perrini,
Balaenoptera_bonaerensis,
Mesoplodon_peruvianus,
Lissodelphis_peronii,
Lissodelphis_borealis,
Balaena_mysticetus,
Caperea_marginata,
Balaenoptera_acutorostrata,
Mesoplodon_ginkgodens,
Megaptera_novaeangliae,
Eschrichtius_robustus,
Moschiola_meminna,
```

```
305  Moschiola_indica,
306  Tragulus_javanicus,
307  Tragulus_kanchil,
308  Tragulus_napu,
309  Okapia_johnstoni,
310  Giraffa_camelopardalis,
311  Antilocapra_americana,
312  Muntiacus_muntjak,
313  Dama_dama,
314  Elaphurus_davidianus,
315  Rusa_unicolor,
316  Cervus_elaphus,
317  Axis_calamianensis,
318  Axis_axis,
319  Rangifer_tarandus,
320  Odocoileus_virginianus,
321  Odocoileus_hemionus,
322  Mazama_gouazoubira,
323  Blastocerus_dichotomus,
324  Pudu_puda,
325  Hydropotes_inermis,
326  Capreolus_capreolus,
327  Alces_alces,
328  Moschus_moschiferus,
329  Moschus_berezovskii,
330  Boselaphus_tragocamelus,
331  Tetracerus_quadricornis,
332  Tragelaphus_imberbis,
333  Tragelaphus_angasii,
334  Tragelaphus_scriptus,
335  Tragelaphus_eurycerus,
336  Tragelaphus_spekii,
337  Tragelaphus_buxtoni,
338  Tragelaphus_strepsiceros,
339  Syncerus_caffer,
340  Bubalus_mindorensis,
341  Bubalus_depressicornis,
342  Bubalus_arnee,
343  Bos_gaurus,
344  Bos_taurus,
345  Bos_indicus,
346  Bison_bonasus,
347  Bos_mutus,
348  Bos_grunniens,
349  Bison_bison,
350  Nesotragus_moschatus,
351  Aepyceros_melampus,
352  Pelea_capreolus,
353  Redunca_fulvorufula,
354  Redunca_redunca,
355  Redunca_arundinum,
356  Kobus_megaceros,
357  Kobus_leche,
358  Kobus_vardonii,
359  Kobus_kob,
360  Kobus_ellipsiprymnus,
361  Ourebia_ourebi,
362  Raphicerus_melanotis,
```

```
Raphicerus_sharpei,
Raphicerus_campestris,
Madoqua_kirkii,
Madoqua_guentheri,
Saiga_tatarica,
Nanger_granti,
Eudorcas_rufifrons,
Gazella_dorcas,
Gazella_gazella,
Gazella_spekei,
Antilope_cervicapra,
Litocranius_walleri,
Antidorcas_marsupialis,
Neotragus_pygmaeus,
Oreotragus_oreotragus,
Philantomba_monticola,
Sylvicapra_grimmia,
Cephalophus_silvicultor,
Cephalophus_dorsalis,
Cephalophus_leucogaster,
Cephalophus_nigrifrons,
Cephalophus_natalensis,
Cephalophus_weynsi,
Ovibos_moschatus,
Capricornis_crispus,
Ovis_canadensis,
Ovis_orientalis,
Ovis_aries,
Ovis_ammon,
Capra_ibex,
Capra_hircus,
Pseudois_nayaur,
Myotragus_balearicus,
Oreamnos_americanus,
Rupicapra_rupicapra,
Ammotragus_lervia,
Connochaetes_taurinus,
Connochaetes_gnou,
Damaliscus_pygargus,
Damaliscus_lunatus,
Beatragus_hunteri,
Alcelaphus_buselaphus,
Hippotragus_niger,
Hippotragus_equinus,
Oryx_dammah,
Oryx_leucoryx,
Oryx_gazella,
Oryx_beisa,
Rhinopoma_hardwickii,
Rhinopoma_microphyllum,
Lavia_frons,
Megaderma_spasma,
Rhinolophus_hipposideros,
Asellia_tridens,
Hipposideros_caffer,
Hipposideros_diadema,
Cynopterus_titthaecheilus,
Cynopterus_brachyotis,
```

421 Epomops_franqueti,
422 Hypsignathus_monstrosus,
423 Rousettus_aegyptiacus,
424 Eidolon_helvum,
425 Pteropus_giganteus,
426 Pteropus_rufus,
427 Pteropus_vampyrus,
428 Macroglossus_minimus,
429 Myzopoda_aurita,
430 Mystacina_tuberculata,
431 Thyroptera_discifera,
432 Noctilio_leporinus,
433 Pteronotus_parnellii,
434 Mormoops_megalophylla,
435 Micronycteris_microtis,
436 Anoura_caudifer,
437 Glossophaga_soricina,
438 Glossophaga_morenoi,
439 Phylloderma_stenops,
440 Phyllostomus_discolor,
441 Lonchorhina_inusitata,
442 Chrotopterus_auritus,
443 Vampyrum_spectrum,
444 Lonchophylla_inexpectata,
445 Carollia_perspicillata,
446 Artibeus_jamaicensis,
447 Artibeus_planirostris,
448 Pygoderma_bilabiatum,
449 Vampyressa_pusilla,
450 Sturnira_erythromos,
451 Sturnira_aratathomasi,
452 Rhinophylla_pumilio,
453 Lonchorhina_orinocensis,
454 Lonchorhina_fernandezi,
455 Lonchorhina_marinkellei,
456 Lonchorhina_aurita,
457 Diphylla_ecaudata,
458 Desmodus_rotundus,
459 Diaemus_youngi,
460 Macrotus_waterhousii,
461 Macrotus_californicus,
462 Nyctinomops_macrotis,
463 Molossus_molossus,
464 Promops_nasutus,
465 Molossops_temminckii,
466 Cynomops_planirostris,
467 Eumops_perotis,
468 Tadarida_brasiliensis,
469 Molossus_currentium,
470 Myotis_daubentonii,
471 Myotis_keaysi,
472 Myotis_velifer,
473 Myotis_chiloensis,
474 Myotis_lucifugus,
475 Myotis_evotis,
476 Eptesicus_nilssonii,
477 Eptesicus_serotinus,
478 Histiotus_montanus,

479 Histiotus_magellanicus,
480 Eptesicus_diminutus,
481 Lasionycteris_noctivagans,
482 Pipistrellus_pipistrellus,
483 Nyctalus_noctula,
484 Vespertilio_murinus,
485 Chalinolobus_tuberculatus,
486 Neoromicia_nana,
487 Lasiurus_cinereus,
488 Lasiurus_ega,
489 Antrozous_pallidus,
490 Otonycteris_hemprichii,
491 Plecotus_auritus,
492 Euderma_maculatum,
493 Barbastella_barbastellus,
494 Miniopterus_schreibersii,
495 Nycteris_javanica,
496 Nycteris_thebaica,
497 Saccopteryx_bilineata,
498 Peropteryx_leucoptera,
499 Balantiopteryx_plicata,
500 Galeopterus_variegatus,
501 Cynocephalus_volans,
502 Nycticebus_coucang,
503 Loris_tardigradus,
504 Otolemur_garnettii,
505 Otolemur_crassicaudatus,
506 Galago_senegalensis,
507 Euoticus_elegantulus,
508 Perodicticus_potto,
509 Arctocebus_calabarensis,
510 Daubentonia_madagascariensis,
511 Indri_indri,
512 Propithecus_diadema,
513 Propithecus_verreauxi,
514 Propithecus_coquereli,
515 Avahi_laniger,
516 Varecia_variegata,
517 Lemur_catta,
518 Hapalemur_griseus,
519 Eulemur_macaco,
520 Eulemur_rubriventer,
521 Eulemur_mongoz,
522 Eulemur_collaris,
523 Eulemur_fulvus,
524 Eulemur_sanfordi,
525 Eulemur_albifrons,
526 Lepilemur_mustelinus,
527 Cheirogaleus_medius,
528 Microcebus_murinus,
529 Microcebus_rufus,
530 Tarsius_syrichta,
531 Pithecia_irrorata,
532 Pithecia_monachus,
533 Pithecia_pithecia,
534 Cacajao_melanocephalus,
535 Callicebus_moloch,
536 Leontocebus_nigricollis,

```
Saguinus_oedipus,
Leontopithecus_rosalia,
Mico_humeralifer,
Callithrix_jacchus,
Cebuella_pygmaea,
Saimiri_sciureus,
Cebus_olivaceus,
Cebus_albifrons,
Cebus_capucinus,
Aotus_azarae,
Aotus_nancymaae,
Alouatta_seniculus,
Alouatta_caraya,
Alouatta_palliata,
Alouatta_pigra,
Lagothrix_lagotricha,
Brachyteles_hypoxanthus,
Brachyteles_arachnoides,
Ateles_geoffroyi,
Ateles_fusciceps,
Ateles_paniscus,
Ateles_chamek,
Ateles_belzebuth,
Symphalangus_syndactylus,
Hylobates_pileatus,
Hylobates_lar,
Hylobates_muelleri,
Hylobates_moloch,
Hylobates_klossii,
Hylobates_agilis,
Nomascus_concolor,
Nomascus_gabriellae,
Nomascus_leucogenys,
Hoolock_hoolock,
Pongo_pygmaeus,
Pongo_abelii,
Pan_troglodytes,
Pan_paniscus,
Homo_sapiens,
Gorilla_gorilla,
Gorilla_beringei,
Semnopithecus_schistaceus,
Trachypithecus_cristatus,
Trachypithecus_phayrei,
Semnopithecus_entellus,
Presbytis_rubicunda,
Presbytis_melalophos,
Rhinopithecus_bieti,
Rhinopithecus_roxellana,
Nasalis_larvatus,
Colobus_guereza,
Colobus_vellerosus,
Macaca_leonina,
Macaca_sinica,
Macaca_silenus,
Macaca_nemestrina,
Macaca_maura,
Macaca_radiata,
```

Macaca_fascicularis,
Macaca_arctoides,
Macaca_nigra,
Macaca_fuscata,
Macaca_mulatta,
Macaca_sylvanus,
Theropithecus_gelada,
Papio_cynocephalus,
Papio_papio,
Papio_hamadryas,
Papio_anubis,
Lophocebus_aterrimus,
Lophocebus_albigena,
Cercocebus_atys,
Mandrillus_sphinx,
Mandrillus_leucophaeus,
Cercopithecus_mitis,
Cercopithecus_ascanius,
Erythrocebus_patas,
Chlorocebus_pygerythrus,
Chlorocebus_aethiops,
Chlorocebus_cynosuros,
Chlorocebus_sabaeus,
Miopithecus_talapoin,
Ptilocercus_lowii,
Tupaia_glis,
Tupaia_nicobarica,
Urogale_everetti,
Tupaia_tana,
Dendrogale_melanura,
Anathana_ellioti,
Sylvilagus_audubonii,
Oryctolagus_cuniculus,
Pentalagus_furnessi,
Lepus_californicus,
Lepus_alleni,
Lepus_townsendii,
Lepus_timidus,
Lepus_europaeus,
Lepus_americanus,
Nesolagus_timminsi,
Nesolagus_netscheri,
Ochotona_hyperborea,
Ochotona_princeps,
Muscardinus_avellanarius,
Aplodontia_rufa,
Ratufa_indica,
Callosciurus_erythraeus,
Tamias_striatus,
Tamias_minimus,
Spermophilus_dauricus,
Ictidomys_tridecemlineatus,
Cynomys_ludovicianus,
Marmota_monax,
Marmota_caligata,
Marmota_marmota,
Marmota_sibirica,
Sciurus_anomalus,

```
Sciurus_niger,
Sciurus_carolinensis,
Sciurus_griseus,
Microsciurus_mimulus,
Microsciurus_alfari,
Microsciurus_flaviventer,
Sciurus_vulgaris,
Tamiasciurus_hudsonicus,
Glaucomys_volans,
Petaurista_leucogenys,
Petaurista_philippensis,
Petaurista_alborufus,
Petaurista_grandis,
Laonastes_aenigmamus,
Atherurus_macrourus,
Hystrix_javanica,
Hystrix_cristata,
Thryonomys_swinderianus,
Heterocephalus_glaber,
Heliophobius_argenteocinereus,
Cryptomys_hottentotus,
Georychus_capensis,
Bathyergus_suillus,
Cuniculus_paca,
Myoprocta_acouchy,
Dasyprocta_leporina,
Dasyprocta_azarae,
Hydrochoerus_hydrochaeris,
Dolichotis_patagonum,
Cavia_porcellus,
Cavia_aperea,
Erethizon_dorsatum,
Coendou_prehensilis,
Lagostomus_maximus,
Chinchilla_lanigera,
Chinchilla_chinchilla,
Phyllomys_sulinus,
Myocastor_coypus,
Capromys_pilorides,
Ctenomys_boliviensis,
Ctenomys_talarum,
Octodon_degus,
Spalacopus_cyanus,
Dipus_sagitta,
Jaculus_orientalis,
Jaculus_jaculus,
Stylodipus_telum,
Allactaga_euphratica,
Allactaga_major,
Zapus_princeps,
Sicista_concolor,
Spalax_leucodon,
Rhizomys_pruinosus,
Dicrostonyx_groenlandicus,
Ondatra_zibethicus,
Lemmus_sibiricus,
Synaptomys_cooperi,
Ellobius_talpinus,
```

```
Microtus_arvalis,
Microtus_pennsylvanicus,
Microtus_ochrogaster,
Microtus_chrotorrhinus,
Microtus_oeconomus,
Myodes_gapperi,
Mesocricetus_auratus,
Tscherskia_triton,
Cricetus_cricetus,
Cricetulus_migratorius,
Cricetulus_longicaudatus,
Cricetulus_barabensis,
Phodopus_sungorus,
Neotoma_fuscipes,
Neotoma_lepida,
Neotoma_floridana,
Neotoma_albigula,
Neotoma_mexicana,
Neotoma_cinerea,
Hodomys_alleni,
Nelsonia_neotomodon,
Xenomys_nelsoni,
Ochrotomys_nuttalli,
Reithrodontomys_creper,
Reithrodontomys_mexicanus,
Reithrodontomys_fulvescens,
Reithrodontomys_sumichrasti,
Reithrodontomys_megalotis,
Reithrodontomys_humulis,
Reithrodontomys_montanus,
Isthmomys_pirrensis,
Isthmomys_flavidus,
Onychomys_torridus,
Onychomys_leucogaster,
Peromyscus_maniculatus,
Peromyscus_leucopus,
Megadontomys_thomasi,
Peromyscus_yucatanicus,
Peromyscus_guatemalensis,
Peromyscus_mexicanus,
Peromyscus_megalops,
Peromyscus_melanophrys,
Habromys_lepturus,
Habromys_lophurus,
Peromyscus_crinitus,
Peromyscus_ochraventer,
Peromyscus_truei,
Peromyscus_difficilis,
Peromyscus_attwateri,
Peromyscus_furvus,
Peromyscus_spicilegus,
Peromyscus_boylii,
Podomys_floridanus,
Neotomodon_alstoni,
Peromyscus_californicus,
Peromyscus_eremicus,
Osgoodomys_banderanus,
Scotinomys_xerampelinus,
```

769 Scotinomys_teguina,
770 Baiomys_taylori,
771 Baiomys_musculus,
772 Tylomys_panamensis,
773 Tylomys_mirae,
774 Tylomys_watsoni,
775 Tylomys_fulviventer,
776 Tylomys_nudicaudus,
777 Ototylomys_phyllotis,
778 Nyctomys_sumichrasti,
779 Chibchanomys_trichotis,
780 Reithrodon_auritus,
781 Rheomys_underwoodi,
782 Rheomys_thomasi,
783 Rheomys_mexicanus,
784 Rheomys_raptor,
785 Ichthyomys_pittieri,
786 Ichthyomys_hydrobates,
787 Ichthyomys_tweedii,
788 Neusticomys_venezuelae,
789 Neusticomys_ferreirai,
790 Neusticomys_monticolus,
791 Anotomys_leander,
792 Chinchillula_sahamae,
793 Sigmodon_leucotis,
794 Sigmodon_fulviventer,
795 Sigmodon_alleni,
796 Sigmodon_ochrognathus,
797 Sigmodon_hispidus,
798 Rhipidomys_macconnelli,
799 Rhipidomys_nitela,
800 Rhipidomys_mastacalis,
801 Rhipidomys_fulviventer,
802 Rhipidomys_venustus,
803 Rhipidomys_couesi,
804 Rhipidomys_venezuelae,
805 Rhipidomys_latimanus,
806 Rhipidomys_austrinus,
807 Aepeomys_lugens,
808 Thomasomys_gracilis,
809 Thomasomys_cinereus,
810 Thomasomys_rhoadsi,
811 Thomasomys_baeops,
812 Thomasomys_aureus,
813 Thomasomys_pyrrhonotus,
814 Thomasomys_daphne,
815 Thomasomys_oreas,
816 Thomasomys_hylophilus,
817 Thomasomys_paramorum,
818 Chilomys_instans,
819 Akodon_bogotensis,
820 Blarinomys_breviceps,
821 Scapteromys_tumidus,
822 Scapteromys_meridionalis,
823 Scapteromys_aquaticus,
824 Kunsia_tomentosus,
825 Thalpomys_lasiotis,
826 Necromys_amoenus,

Necromys_urichi,
Necromys_lasiurus,
Necromys_temchuki,
Necromys_obscurus,
Necromys_lactens,
Akodon_mimus,
Akodon_azarae,
Akodon_boliviensis,
Akodon_subfuscus,
Akodon_lutescens,
Akodon_cursor,
Akodon_simulator,
Akodon_albiventer,
Akodon_mollis,
Akodon_torques,
Akodon_aerosus,
Deltamys_kempi,
Thaptomys_nigrita,
Oxymycterus_delator,
Oxymycterus_paramensis,
Oxymycterus_rufus,
Oxymycterus_inca,
Zygodontomys_brevicauda,
Oecomys_concolor,
Oecomys_roberti,
Oecomys_bicolor,
Oecomys_superans,
Oecomys_trinitatis,
Oecomys_mamorae,
Oecomys_paricola,
Transandinomys_talamancae,
Transandinomys_bolivaris,
Euryoryzomys_nitidus,
Euryoryzomys_russatus,
Euryoryzomys_legatus,
Nephelomys_albigularis,
Nephelomys_keaysi,
Hylaeamys_megacephalus,
Handleyomys_alfaroi,
Handleyomys_melanotis,
Handleyomys_chapmani,
Neacomys_guianae,
Neacomys_spinosus,
Neacomys_tenuipes,
Microryzomys_minutus,
Microryzomys_altissimus,
Oligoryzomys_microtis,
Oligoryzomys_nigripes,
Oligoryzomys_destructor,
Oligoryzomys_fulvescens,
Oligoryzomys_eliurus,
Oligoryzomys_delticola,
Oligoryzomys_chacoensis,
Oligoryzomys_andinus,
Oligoryzomys_magellanicus,
Oligoryzomys_longicaudatus,
Oligoryzomys_flavescens,
Eremoryzomys_polius,

885 Cerradomys_subflavus,
886 Sooretamys_angouya,
887 Holochilus_brasiliensis,
888 Holochilus_chacarius,
889 Pseudoryzomys_simplex,
890 Lundomys_molitor,
891 Nectomys_squamipes,
892 Oryzomys_couesi,
893 Oryzomys_palustris,
894 Sigmodontomys_alfari,
895 Melanomys_caliginosus,
896 Nesoryzomys_indefessus,
897 Irenomys_tarsalis,
898 Euneomys_petersoni,
899 Euneomys_chinchilloides,
900 Andinomys_edax,
901 Punomys_kofordi,
902 Delomys_sublineatus,
903 Delomys_dorsalis,
904 Calomys_lepidus,
905 Calomys_sorellus,
906 Calomys_musculinus,
907 Calomys_laucha,
908 Calomys_tener,
909 Calomys_boliviae,
910 Calomys_callosus,
911 Calomys_venustus,
912 Calomys_hummelincki,
913 Eligmodontia_morgani,
914 Eligmodontia_puerulus,
915 Eligmodontia_moreni,
916 Eligmodontia_hirtipes,
917 Eligmodontia_bolsonensis,
918 Graomys_griseoflavus,
919 Graomys_domorum,
920 Andalgalomys_pearsoni,
921 Andalgalomys_olrogi,
922 Salinomys_delicatus,
923 Phyllotis_wolffsohni,
924 Tapecomys_primus,
925 Auliscomys_sublimis,
926 Auliscomys_pictus,
927 Auliscomys_boliviensis,
928 Galenomys_garleppi,
929 Loxodontomys_micropus,
930 Phyllotis_osilae,
931 Phyllotis_alisosiensis,
932 Phyllotis_anitae,
933 Phyllotis_caprinus,
934 Phyllotis_xanthopygus,
935 Phyllotis_darwini,
936 Phyllotis_amicus,
937 Paralomys_gerbillus,
938 Phyllotis_haggardi,
939 Phyllotis_andium,
940 Calassomys_apicalis,
941 Akodon_neocenus,
942 Wiedomys_pyrrhorhinos,

943 Juliomys_pictipes,
944 Abrothrix_olivaceus,
945 Abrothrix_andinus,
946 Abrothrix_jelskii,
947 Abrothrix_illuteus,
948 Abrothrix_longipilis,
949 Abrothrix_sanborni,
950 Notiomys_edwardsii,
951 Geoxus_valdivianus,
952 Chelemys_macronyx,
953 Mystromys_albicaudatus,
954 Saccostomus_campestris,
955 Beamys_hindei,
956 Cricetomys_gambianus,
957 Brachytarsomys_albicauda,
958 Gymnuromys_roberti,
959 Eliurus_myoxinus,
960 Brachyuromys_ramirohitra,
961 Nesomys_rufus,
962 Nesomys_audeberti,
963 Macrotarsomys_bastardi,
964 Calomyscus_baluchi,
965 Lophiomys_imhausi,
966 Pachyuromys_duprasi,
967 Psammomys_obesus,
968 Meriones_shawi,
969 Meriones_unguiculatus,
970 Sekeetamys_calurus,
971 Ammodillus_imbellis,
972 Microdillus_peeli,
973 Gerbillus_cheesmani,
974 Taterillus_arenarius,
975 Gerbilliscus_nigricaudus,
976 Gerbillurus_setzeri,
977 Desmodillus_auricularis,
978 Tatera_indica,
979 Notomys_amplus,
980 Conilurus_albipes,
981 Hydromys_chrysogaster,
982 Mastomys_coucha,
983 Mus_musculus,
984 Rhabdomys_pumilio,
985 Micromys_minutus,
986 Maxomys_inflatus,
987 Rattus_rattus,
988 Rattus_fuscipes,
989 Rattus_norvegicus,
990 Pedetes_surdaster,
991 Pedetes_capensis,
992 Anomalurus_pelii,
993 Anomalurus_derbianus,
994 Anomalurus_beecrofti,
995 Anomalurus_pusillus,
996 Microdipodops_pallidus,
997 Dipodomys_spectabilis,
998 Dipodomys_ordii,
999 Dipodomys_heermanni,
1000 Dipodomys_merriami,

1001 Thomomys_bulbivorus,
1002 Thomomys_umbrinus,
1003 Thomomys_bottae,
1004 Geomys_pinetis,
1005 Geomys_bursarius,
1006 Geomys_arenarius,
1007 Cratogeomys_castanops,
1008 Liomys_salvini,
1009 Heteromys_anomalus,
1010 Perognathus_fasciatus,
1011 Castor_fiber,
1012 Castor_canadensis,
1013 Hydrodamalis_gigas,
1014 Trichechus_manatus,
1015 Trichechus_senegalensis,
1016 Trichechus_inunguis,
1017 Dugong_dugon,
1018 Loxodonta_africana,
1019 Elephas_maximus,
1020 Procavia_capensis,
1021 Heterohyrax_brucei,
1022 Dendrohyrax_validus,
1023 Dendrohyrax_dorsalis,
1024 Dendrohyrax_arboreus,
1025 Orycteropus_afer,
1026 Rhynchocyon_cirnei,
1027 Rhynchocyon_chrysopygus,
1028 Rhynchocyon_petersi,
1029 Elephantulus_myurus,
1030 Elephantulus_edwardii,
1031 Macroscelides_proboscideus,
1032 Petrodromus_tetradactylus,
1033 Elephantulus_rufescens,
1034 Elephantulus_brachyrhynchus,
1035 Elephantulus_intufi,
1036 Elephantulus_rupestris,
1037 Potamogale_velox,
1038 Limnogale_mergulus,
1039 Microgale_talazaci,
1040 Microgale_longicaudata,
1041 Microgale_cowani,
1042 Oryzorictes_tetradactylus,
1043 Tenrec_ecaudatus,
1044 Hemicentetes_semispinosus,
1045 Setifer_setosus,
1046 Echinops_telfairi,
1047 Eremitalpa_granti,
1048 Amblysomus_hottentotus,
1049 Calcochloris_obtusirostris,
1050 Chrysospalax_trevelyani,
1051 Chrysochloris_asiatica,
1052 Dasypus_hybridus,
1053 Dasypus_novemcinctus,
1054 Dasypus_kappleri,
1055 Dasypus_septemcinctus,
1056 Zaedyus_pichiy,
1057 Euphractus_sexcinctus,
1058 Chaetophractus_vellerosus,

1059 Chaetophractus_villosus,
1060 Chlamyphorus_truncatus,
1061 Tolypeutes_matacus,
1062 Priodontes_maximus,
1063 Cabassous_unicinctus,
1064 Tamandua_tetradactyla,
1065 Tamandua_mexicana,
1066 Myrmecophaga_tridactyla,
1067 Cyclopes_didactylus,
1068 Choloepus_hoffmanni,
1069 Choloepus_didactylus,
1070 Bradypus_tridactylus,
1071 Bradypus_variegatus,
1072 Rhyncholestes_raphanurus,
1073 Caenolestes_fuliginosus,
1074 Glironia_venusta,
1075 Monodelphis_domestica,
1076 Gracilinanus_microtarsus,
1077 Metachirus_nudicaudatus,
1078 Didelphis_virginiana,
1079 Didelphis_marsupialis,
1080 Didelphis_albiventris,
1081 Chironectes_minimus,
1082 Caluromys_philander,
1083 Dromiciops_gliroides,
1084 Notoryctes_typhlops,
1085 Notoryctes_caurinus,
1086 Macrotis_lagotis,
1087 Isoodon_macrourus,
1088 Isoodon_obesulus,
1089 Perameles_gunnii,
1090 Perameles_nasuta,
1091 Sarcophilus_harrisii,
1092 Dasyurus_maculatus,
1093 Dasyurus_viverrinus,
1094 Dasyuroides_byrnei,
1095 Dasycercus_cristicauda,
1096 Antechinus_flavipes,
1097 Thylacinus_cynocephalus,
1098 Myrmecobius_fasciatus,
1099 Phascolarctos_cinereus,
1100 Vombatus_ursinus,
1101 Lasiorhinus_latifrons,
1102 Cercartetus_concinnus,
1103 Trichosurus_vulpecula,
1104 Spilocuscus_maculatus,
1105 Phalanger_sericeus,
1106 Phalanger_orientalis,
1107 Hypsiprymnodon_moschatus,
1108 Thylogale_billardierii,
1109 Petrogale_lateralis,
1110 Petrogale_penicillata,
1111 Dendrolagus_goodfellowi,
1112 Dendrolagus_matschiei,
1113 Dendrolagus_bennettianus,
1114 Dendrolagus_lumholtzi,
1115 Macropus_fuliginosus,
1116 Macropus_giganteus,

```
		1117	Macropus_antilopinus,
		1118	Macropus_robustus,
		1119	Macropus_rufus,
		1120	Macropus_rufogriseus,
		1121	Macropus_eugenii,
		1122	Macropus_agilis,
		1123	Macropus_parryi,
		1124	Macropus_parma,
		1125	Macropus_irma,
		1126	Dendrolagus_ursinus,
		1127	Potorous_longipes,
		1128	Potorous_tridactylus,
		1129	Bettongia_penicillata,
		1130	Bettongia_gaimardi,
		1131	Aepyprymnus_rufescens,
		1132	Pseudocheirus_peregrinus,
		1133	Petauroides_volans,
		1134	Petaurus_australis,
		1135	Petaurus_norfolcensis,
		1136	Petaurus_breviceps
	;
	TREE * UNTITLED = [&R]
((((((((((((((((((((((((((((((936:2.044496555,937:2.044496555):2.542109
127,(938:4.407780758,939:4.407780758):0.1788249241):2.669728633,((933:1.9
65411554,934:1.965411554):2.495998815,935:4.461410369):2.794923945):0.967
8246599,((931:0.2849880866,932:0.2849880866):4.206942025,930:4.491930112)
:3.732228863):0.7863484566,((((926:1.42353437,927:1.42353437):3.457163474
,925:4.880697844):1.865807412,928:6.746505255):1.18730483,929:7.933810085
):1.076697346):0.3127279446,(923:3.864772096,924:3.864772096):5.45846328)
:1.874301945,((920:5.222449919,921:5.222449919):4.44027203,922:9.66272194
9):1.534815372):1.59082862,(((((914:2.052991599,915:2.052991599):0.868270
9337,916:2.921262533):0.3761576158,913:3.297420149):1.347687349,917:4.645
107498):6.151038559,(918:3.176890328,919:3.176890328):7.619255728):1.9922
19885):0.6841469014,(((((908:1.903711,909:1.903711):3.432641246,(910:0.84
4395199,911:0.844395199):4.491957047):1.022536943,907:6.358889188):2.3314
32317,912:8.690321505):0.480318389,((905:2.787565414,906:2.787565414):1.2
35051311,904:4.022616725):5.148023169):4.301872948):0.6003519411,(940:0.8
517824889,941:0.8517824889):13.22108229):0.7202339256,(902:3.995576832,90
3:3.995576832):10.79752188):1.512057421,(((((944:2.596912694,945:2.596912
694):3.24700791,(946:4.610368446,947:4.610368446):1.233552158):0.15824473
94,(948:4.570587346,949:4.570587346):1.431577997):2.003669651,((950:6.035
330471,951:6.035330471):0.403850996,952:6.439181467):1.566653528):7.03740
169,(942:13.38243421,943:13.38243421):1.66080247):1.261919445):0.26767152
29,(((898:7.505100739,899:7.505100739):2.933804401,897:10.43890514):5.423
204833,(900:5.239498263,901:5.239498263):10.62261171):0.7107176804):1.568
629936,(((((((((877:0.648975078,878:0.648975078):3.267753451,879:3.91672
8529):0.03211150298,876:3.948840031):0.643562285,(874:2.946370534,875:2.9
46370534):1.646031782):1.091159031,(((881:0.7271328301,882:0.7271328301):
2.096090683,880:2.823223513):2.307350762,883:5.130574275):0.552987072):2.
446309754,873:8.129871101):3.96028245,(((869:1.286308921,870:1.286308921)
:6.753505604,868:8.039814525):1.624681955,(871:0.6188418195,872:0.6188418
195):9.04565466):2.425657071):1.324435124,((((((((894:2.961819764,895:2.9
61819764):4.380680957,896:7.342500722):1.64928701,(892:4.333927821,893:4.
333927821):4.65785991):0.7143587586,891:9.70614649):0.3928553875,890:10.0
9900188):0.5728530329,((887:4.496992156,888:4.496992156):2.287007728,889:
6.783999885):3.887855026):0.9444070159,(885:8.822864936,886:8.822864936):
2.79339699):1.237320512,884:12.85358244):0.5610062366):0.5022183607,(((((
(((851:3.710167633,852:3.710167633):0.7513981992,853:4.461565832):0.44353
```

10567,(854:4.223778523,855:4.223778523):0.681318366):1.389044093,856:6.29
4140982):0.8178202881,850:7.111961271):2.553664379,(((860:0.2647619639,86
1:0.2647619639):3.239153471,859:3.503915435):2.739943859,(857:3.504929148
,858:3.504929148):2.738930146):3.421766356):1.4836669,((862:5.77074605,86
3:5.77074605):4.507011001,864:10.27775705):0.8715354985):0.1992646677,((8
65:6.332917719,866:6.332917719):0.195102256,867:6.528019975):4.820537243)
:2.568249818):1.143433662,849:15.0602407):3.081216891):0.07252158622,((((
(((((((835:0.3366875868,836:0.3366875868):1.367848082,834:1.704535669):0
.1539871489,833:1.858522818):1.128448248,837:2.986971066):2.285758552,(((
840:1.4251626,841:1.4251626):1.27701375,842:2.70217635):1.124836287,(838:
3.331815007,839:3.331815007):0.4951976302):1.445716981):0.3782641643,832:
5.650993782):1.027984547,843:6.678978329):1.088477205,844:7.767455534):0.
9622720908,(((((828:2.066341335,829:2.066341335):1.807240797,830:3.873582
132):1.591998954,831:5.465581086):1.438240961,(826:6.290672138,827:6.2906
72138):0.6131499088):1.06624133,825:7.970063377):0.759664248):3.950688302
,(((846:2.775341696,847:2.775341696):1.619275282,845:4.394616978):0.03653
390269,848:4.431150881):8.249265046):0.7591471858,((((822:1.364990599,823
:1.364990599):0.2451525997,821:1.610143199):7.521861031,824:9.13200423):2
.792170752,820:11.92417498):1.515388131):3.199422199,((((((((813:2.302642
337,814:2.302642337):4.245410397,(815:4.291408145,816:4.291408145):2.2566
44589):0.3224605147,817:6.870513248):1.278303216,((810:0.2603153066,811:0
.2603153066):7.005968333,812:7.266283639):0.8825328255):0.02991981897,(80
8:0.3580680555,809:0.3580680555):7.820668228):3.713692804,(818:0.83902715
89,819:0.8390271589):11.05340193):0.6708766981,807:12.56330579):1.3905429
13,(((((((799:1.617935292,800:1.617935292):1.935349311,801:3.553284604):1
.32569086,802:4.878975464):0.3412620211,803:5.220237485):1.493862018,(804
:3.086752374,805:3.086752374):3.627347129):1.806881173,798:8.520980676):0
.08334024214,806:8.604320918):5.349527781):2.685136614):1.574993863):6.90
4854173,((((((((((781:0.5228324584,782:0.5228324584):2.363290014,(783:0.26
97725673,784:0.2697725673):2.616349906):1.278165036,((785:0.2553028019,78
6:0.2553028019):3.439651321,787:3.694954123):0.4693333861):2.01784964,780
:6.182137149):1.57631427,((788:1.190909569,789:1.190909569):2.820478342,7
90:4.011387911):3.747063508):4.695864277,791:12.4543157):0.7267172539,792
:13.18103295):3.82409215,779:17.0051251):2.625812779,(((795:5.396283795,7
96:5.396283795):0.6932402609,797:6.089524056):3.103110339,(793:7.27576159
6,794:7.275761596):1.916872799):10.43830348):5.487895469):7.28105008,((((
((773:0.6440781655,774:0.6440781655):0.2925290059,775:0.9366071714):2.098
431987,776:3.035039158):0.3146664516,772:3.34970561):7.649525865,777:10.9
9923147):16.14541117,778:27.14464265):5.255240783):1.83058513,((((((((((
(((756:5.799384026,757:5.799384026):0.06632050512,(758:4.703045299,759:4.
703045299):1.162659232):0.3777264454,((761:4.130920921,762:4.130920921):1
.360026127,760:5.490947048):0.7524839286):0.1500304229,755:6.393461399):1
.185083506,(753:4.903524069,754:4.903524069):2.675020837):0.3797686747,(7
63:5.498532034,764:5.498532034):2.459781547):1.558096619,((((749:3.156344
481,750:3.156344481):1.267607632,748:4.423952113):1.263975551,(751:5.0802
28696,752:5.080228696):0.6076989674):2.873946977,747:8.561874641):0.95453
55589):1.387272888,(745:5.031023267,746:5.031023267):5.872659821):0.71515
78621,((765:7.876190834,766:7.876190834):0.8254457635,767:8.701636598):2.
917204352):3.880009234,(743:6.534206354,744:6.534206354):8.96464383):1.32
7773603,((((((737:3.419664069,738:3.419664069):3.563213746,739:6.98287781
5):0.907123553,740:7.890001368):0.7645484487,736:8.654549816):1.590370096
,(734:7.507651453,735:7.507651453):2.737268459):3.776843441,(741:2.767380
248,742:2.767380248):11.2543831):2.804860435):6.171067118,((768:2.5822883
62,769:2.582288362):13.05533336,(770:8.581278337,771:8.581278337):7.05634
3387):7.360069181):2.489341665,733:25.48703257):1.249811694,((((((726:5.3
15222225,727:5.315222225):0.1064173169,728:5.421639542):1.472195446,729:6
.893834988):0.5771042355,725:7.470939223):0.6549307172,724:8.125869941):5
.247333275,((730:0.8557181345,731:0.8557181345):10.8674984,732:11.7232165

4):1.649986679):13.36364105):7.493624294):0.1556174365,(((((((((713:6.99
7715623,714:6.997715623):0.1246175215,712:7.122333145):0.5868634845,711:7
.709196629):1.440607806,715:9.149804435):1.893595606,710:11.04340004):0.8
469103145,716:11.89031035):0.8648128718,(708:8.148839258,709:8.148839258)
:4.606283969):0.03157192374,707:12.78669515):0.7149048365,706:13.50159999
):19.84039991,(((((721:4.209291878,722:4.209291878):4.188150096,720:8.397
441974):1.427067931,(718:9.144890154,719:9.144890154):0.679619751):9.5998
65993,717:19.4243759):2.096720378,723:21.52109628):11.82090362):1.0440860
96):9.804778764,(((((961:5.130073357,962:5.130073357):8.1656864,960:13.29
575976):9.107556405,963:22.40331616):3.718762367,((958:16.69150438,959:16
.69150438):5.512534949,957:22.20403933):3.9180392):8.027943868,(((955:16.
48248209,956:16.48248209):9.737653645,954:26.22013574):6.741478987,953:32
.96161472):1.188407674):10.04084236):2.473701972,(((((((((970:0.08905218
162,971:0.08905218162):1.187586306,972:1.276638488):9.592963941,973:10.86
960243):3.739649071,974:14.6092515):0.8098591226,((968:2.818468591,969:2.
818468591):7.605619215,967:10.42408781):4.995022816):4.182077305,966:19.6
0118793):2.765204599,(((975:11.74367884,976:11.74367884):5.670993638,977:
17.41467248):1.422385242,978:18.83705773):3.5293348):20.26027632,965:42.6
2666884):2.448558108,((((979:10.99383779,980:10.99383779):0.9810682416,98
1:11.97490603):14.12407418,((982:22.03440973,983:22.03440973):2.588708003
,984:24.62311773):1.475862474):0.5369734673,((((988:6.025120696,989:6.025
120696):0.6305542469,987:6.655674943):10.3619164,986:17.01759134):7.68852
1198,985:24.70611254):1.929841134):18.43927328):5.127054297e-
05,964:45.07527822):1.589288508):14.28478616,(704:35.19079367,705:35.1907
9367):25.75855922):3.980138607,((((((697:3.618652316,698:3.618652316):3.1
74274506,699:6.792926822):0.9976357257,696:7.790562547):6.351333324,(700:
9.490868891,701:9.490868891):4.65102698):13.27154644,702:27.41344231):9.9
5234742,703:37.36578973):27.56370177):1.780117106,(((992:2.599023325,993:
2.599023325):1.826910313,(994:3.538772526,995:3.538772526):0.887161112):3
5.72229883,(990:3.996562947,991:3.996562947):36.15166952):26.56137613):0.
4831600095,((((((1005:2.46476153,1006:2.46476153):5.383010428,1004:7.847
771958):0.6803819231,1007:8.528153881):2.71293184,((1002:2.370004994,1003
:2.370004994):5.221383917,1001:7.591388911):3.64969681):11.99070056,((100
8:12.72705769,1009:12.72705769):9.357310657,1010:22.08436834):1.147417937
):1.608882816,((((998:5.487976882,999:5.487976882):1.377968391,1000:6.865
945273):2.192845598,997:9.058790871):7.191591324,996:16.25038219):8.59028
6901):23.68925458,(1011:5.585109854,1012:5.585109854):42.94481383):18.662
84493):2.120543194,(((((((((692:3.561002508,693:3.561002508):11.52930186
,(694:4.367015749,695:4.367015749):10.72328862):2.670739299,((689:12.6822
0086,690:12.68220086):2.718075678,691:15.40027653):2.360767135):10.927559
25,((687:1.951891596,688:1.951891596):11.10183836,686:13.05372996):15.634
87296):0.888445952,(684:6.361670764,685:6.361670764):23.2153781):0.784721
0641,((((680:13.23389126,681:13.23389126):1.772732702,(682:1.2373348,683:
1.2373348):13.76928916):6.196352618,((678:2.926558515,679:2.926558515):5.
123307367,677:8.049865882):13.1531107):0.6620909569,676:21.86506754):8.49
6702396):5.889833791,(((((674:7.129630773,675:7.129630773):3.589324356,67
3:10.71895513):3.409683635,672:14.12863876):12.38588154,671:26.5145203):5
.481691633,670:31.99621194):4.255391786):2.569542123,((668:3.199331723,66
9:3.199331723):3.007733339,667:6.207065062):32.61408078):16.93924452,666:
55.76039037):1.505207604,(((((((((((656:4.76035366,657:4.76035366):0.33
58334575,658:5.096187118):0.478678827,655:5.574865945):0.3155011653,654:5
.89036711):2.002655257,653:7.893022366):0.7220574158,652:8.615079782):0.6
993119337,659:9.314391716):2.830881417,660:12.14527313):7.122726985,((((6
63:1.718967794,664:1.718967794):5.085686262,665:6.804654056):2.569902236,
662:9.374556292):6.917800464,661:16.29235676):2.975643362):4.75778537,(((
(((649:3.02631311,650:3.02631311):0.4441013383,651:3.470414448):0.7203455
824,648:4.19076003):3.631316631,((646:5.36631681,647:5.36631681):1.203813
155,645:6.570129965):1.251946696):8.951200684,(643:6.174263156,644:6.1742

63156):10.59901419):6.53929561,642:23.31257295):0.7132125332):3.211909727
,641:27.23769521):2.229092984,640:29.4667882):17.00825475,639:46.47504294
):10.79055503):12.04771383):1.223635293,((((((631:2.416684946,632:2.4166
84946):1.509003822,633:3.925688768):1.24498625,(629:0.9596425718,630:0.95
96425718):4.211032446):1.92387726,634:7.094552278):6.208026518,((627:11.3
3742933,628:11.33742933):0.6579228637,626:11.99535219):1.307226603):8.471
953348,(635:0.1453406627,636:0.1453406627):21.62919148):27.09860666,(637:
6.160571787,638:6.160571787):42.71256702):21.66380829):7.401176797,((((((
(((((((((597:0.8956057783,598:0.8956057783):0.3084623234,599:1.204068102
):0.7118863166,596:1.915954418):0.3059562244,595:2.221910643):0.381379748
2,594:2.603290391):1.168741588,((((591:1.338754273,592:1.338754273):0.198
6986342,590:1.537452907):0.1817857894,589:1.719238696):1.617190986,593:3.
336429683):0.4356022961):1.23953795,600:5.011569929):2.368511927,((((((60
4:0.969004275,605:0.969004275):0.47726651,603:1.446270785):1.004409562,60
2:2.450680347):1.350308223,606:3.80098857):0.9144348077,601:4.715423377):
1.8544448,((607:3.624305935,608:3.624305935):1.175688542,(609:3.869423503
,610:3.869423503):0.9305709738):1.769873701):0.8102136785):0.8465569805,(
((((614:1.103823264,615:1.103823264):0.4665126334,616:1.570335897):1.868
716798,617:3.439052695):1.500577781,613:4.939630476):0.461391214,(611:4.5
11068584,612:4.511068584):0.8899531067):2.165977142,618:7.566998833):0.65
96400039):3.943806851,((((((578:1.808113142,579:1.808113142):0.8208035497
,580:2.628916691):3.242198918,581:5.871115609):1.684803091,(582:1.8667287
71,583:1.866728771):5.68918993):0.1693592406,((584:2.871824009,585:2.8718
24009):3.488826935,586:6.360650944):1.364626997):1.370447442,(587:1.35900
9654,588:1.359009654):7.73671573):3.074720304):7.28539306,((((((((564:0.6
065606634,565:0.6065606634):0.5861359086,563:1.192696572):0.04864384233,5
66:1.241340414):0.4894188673,562:1.730759282):0.9140660954,561:2.64482537
7):1.365279536,560:4.010104913):0.1865754759,(((568:1.07989432,569:1.0798
9432):0.3302778216,567:1.410172142):2.399410584,570:3.809582726):0.387097
663):8.482821895,((((573:1.961807787,574:1.961807787):2.247142378,575:4.2
08950165):1.612984779,(576:1.797214971,577:1.797214971):4.024719973):4.14
4758027,(571:2.708883283,572:2.708883283):7.257809688):2.712809313):6.776
336464):11.80137614,(((((((540:4.102344042,541:4.102344042):1.320287833,5
39:5.422631875):4.877562412,538:10.30019429):0.4801583184,(536:7.88440440
4,537:7.884404404):2.895948201):3.541969823,((((543:1.066304333,544:1.066
304333):0.2313748148,545:1.297679147):12.10961345,542:13.4072926):0.76034
21067,(546:3.46538376,547:3.46538376):10.70225094):0.1546877265):2.699730
701,(((((558:0.7076357644,559:0.7076357644):1.182461144,557:1.890096909):
0.9457157717,(555:1.30571922,556:1.30571922):1.53009346):6.199464122,((55
3:0.8307450723,554:0.8307450723):2.538614483,552:3.369359555):5.665917248
):2.662528622,(((550:1.201056851,551:1.201056851):1.251230827,549:2.45228
7678):1.958967423,548:4.411255101):7.286550324):5.324247705):0.9158236293
,((((531:0.1810552962,532:0.1810552962):4.190106231,533:4.371161528):4.33
3659972,534:8.7048215):5.278568971,535:13.98339047):3.954486289):13.31933
813):35.54553651,530:66.8027514):4.129952017,(((((((((((524:0.0695918702,
525:0.0695918702):0.5430825108,523:0.612674381):0.654698023,522:1.2673724
04):0.7283740941,(520:1.733771899,521:1.733771899):0.2619745991):1.037135
252,519:3.03288175):6.466832526,(517:5.703183058,518:5.703183058):3.79653
1218):3.668124049,516:13.16783832):7.566167816,(((528:5.274600251,529:5.2
74600251):9.422663946,527:14.6972642):3.871977325,526:18.56924152):2.1647
64619):2.455603672,((((513:1.859501843,514:1.859501843):3.585345775,512:5
.444847618):3.899221365,515:9.344068983):1.629411762,511:10.97348074):12.
21612907):17.38692415,510:40.57653397):8.9939339,(((((504:2.310165748,505
:2.310165748):4.292832632,506:6.60299838):4.097058576,507:10.70005696):9.
42444647,(508:13.10780959,509:13.10780959):7.016693831):1.239913488,(502:
14.89988624,503:14.89988624):6.46453068):28.20605095):21.36223555):1.1636
28807,(500:9.513215296,501:9.513215296):62.58311692):2.205612582,(((((620
:4.47654706,621:4.47654706):7.471256175,(622:10.26964101,623:10.26964101)

:1.678162224):3.08655143,624:15.03435466):52.75287637,625:67.78723103):2.
765928247,619:70.55315928):3.748785521):3.636179094):9.900059783,(((((((
(((((((((((((389:0.343825471,390:0.343825471):0.9730904141,391:1.316915
885):0.8998766056,388:2.216792491):2.564309889,((392:1.934802432,393:1.93
4802432):2.178450763,394:4.113253195):0.6678491852):0.3775475586,(((397:3
.833289923,398:3.833289923):0.4508800278,396:4.28416995):0.4447754168,395
:4.728945367):0.4297045711):0.6835079939,(386:4.768879677,387:4.768879677
):1.073278255):3.30576141,((((401:1.862258443,402:1.862258443):0.3030186
091,403:2.165277052):0.8615896729,404:3.026866725):2.351756372,(399:0.614
5538831,400:0.6145538831):4.764069214):3.481785345,(((409:0.7120963052,4
10:0.7120963052):0.6108373646,408:1.32293367):0.7125311761,407:2.03546484
6):4.495217587,(405:5.191906226,406:5.191906226):1.338776208):2.329726009
):0.2875109):1.006925202,((((((383:1.10692775,384:1.10692775):1.55370540
8,382:2.660633158):0.6112969272,385:3.271930085):1.674961217,((380:2.7517
10751,381:2.751710751):1.06564736,379:3.817358111):1.129533191):1.1680393
58,378:6.114930659):2.476144275,377:8.591074935):0.2684582382,376:8.85953
3173):1.295311372):0.2459982534,((((((((370:1.391941833,371:1.391941833)
:0.1361551015,372:1.528096935):1.619407243,373:3.147504177):0.3752360195,
369:3.522740197):0.6318954999,368:4.154635697):2.885146622,(374:5.5685776
11,375:5.568577611):1.471204707):0.2939627162,367:7.333745035):1.01804358
7,(((363:2.224456173,364:2.224456173):0.6619974069,362:2.88645358):4.3311
74624,(365:1.256128068,366:1.256128068):5.961500136):1.134160418):0.41781
98714,361:8.769608493):1.631234304):0.134693884,(((((356:2.480313185,357:
2.480313185):0.9320230733,(358:2.244034532,359:2.244034532):1.168301726):
0.2244798958,360:3.636816154):4.320529395,((354:1.995682149,355:1.9956821
49):1.468703418,353:3.464385567):4.492959982):0.4379220344,352:8.39526758
3):2.140269099):1.130291526,(350:9.96929251,351:9.96929251):1.696535698):
0.7438869419,((((((344:0.3740990061,345:0.3740990061):1.647044691,346:2.
021143698):0.6841425178,((347:0.995373848,348:0.995373848):0.2588640995,3
49:1.254237948):1.451048268):0.6675218273,343:3.372808043):4.449295346,((
(341:1.101563396,342:1.101563396):0.7014552817,340:1.803018678):3.0046619
82,339:4.80768066):3.014422729):2.169996776,((((((335:1.27898065,336:1.27
898065):1.191162177,334:2.470142827):0.7342149618,337:3.204357789):0.6432
052169,338:3.847563006):1.149351828,333:4.996914834):0.9805147049,332:5.9
77429539):4.014670626):0.4668102164,(330:5.000787138,331:5.000787138):5.4
58123244):1.950804768):0.5384184084,(328:2.447895485,329:2.447895485):10.
50023807):0.8810748843,((((((322:2.851784905,323:2.851784905):0.642808523
1,324:3.494593428):0.9910536873,(320:1.209147785,321:1.209147785):3.27649
933):0.5901272785,319:5.075774394):2.023854609,((325:2.412492263,326:2.41
2492263):4.041199464,327:6.453691727):0.6459372757):0.3856976688,(((((315
:1.113581437,316:1.113581437):1.913583596,314:3.027165033):1.186350802,31
3:4.213515835):0.166720273,(317:2.23392383,318:2.23392383):2.146312279):1
.387977641,312:5.768213749):1.717112922):6.343881771):1.877440651,((309:6
.557509454,310:6.557509454):6.600756917,311:13.15826637):2.548382722):15.
29823069,(((306:1.604939437,307:1.604939437):3.465908503,308:5.07084794):
14.74887558,(304:2.545930761,305:2.545930761):17.27379276):11.18515626):9
.535366401,((((((((((((((((((((259:0.2752692627,260:0.2752692627):0.308
5913757,(261:0.5296990403,262:0.5296990403):0.05416159813):0.06252696039,
258:0.6463875988):0.06345446603,257:0.7098420649):0.2431172206,256:0.9529
592854):0.1936751244,255:1.14663441):0.26979864,(253:1.10883491,254:1.108
83491):0.3075981397):0.4228105777,252:1.839243627):1.260889586,((250:0.58
04197745,251:0.5804197745):1.675785225,249:2.256205):0.843928214):0.22501
51572,((((267:0.4732294317,268:0.4732294317):0.6933579564,(269:0.60540118
27,270:0.6054011827):0.5611862054):0.527309839,(265:0.8827556068,266:0.88
27556068):0.8111416203):0.2110120687,(263:1.044549164,264:1.044549164):0.
8603601318):1.420239075):0.0466106286,((((((246:0.5423653198,247:0.542365
3198):0.4998617221,248:1.042227042):0.1014236399,245:1.143650682):0.19329
27445,244:1.336943426):0.7796299534,243:2.11657338):1.054825857,242:3.171

399237):0.2003597627):0.373697305,241:3.745456305):0.2143091607,240:3.959
765465):0.4502596708,239:4.410025136):3.069434135,((((235:0.9960859121,23
6:0.9960859121):0.7639727384,(237:0.9694863849,238:0.9694863849):0.790572
2656):0.9686896205,234:2.728748271):2.485485694,((231:0.06312635742,232:0
.06312635742):2.291921817,233:2.355048174):2.859185791):2.265225307):1.71
757535,(((228:1.448290033,229:1.448290033):4.91139975,227:6.359689782):1.
967747275,230:8.327437058):0.8695975638):1.555760376,(((((((((279:1.94061
5665,280:1.940615665):0.4118703361,278:2.352486001):1.102988559,281:3.455
474561):0.3722470061,((283:2.254963695,284:2.254963695):1.1706237,282:3.4
25587395):0.4021341717):0.3778918148,(276:2.034421747,277:2.034421747):2.
171191634):0.9704761505,(274:0.4126057709,275:0.4126057709):4.763483761):
0.1115062235,273:5.287595755):0.6075015758,285:5.895097331):0.2282876387,
(271:0.444778819,272:0.444778819):5.678606151):4.629410028):0.9057316166,
226:11.65852661):0.3744766241,((223:3.442065007,224:3.442065007):6.635709
293,225:10.0777743):1.955228939):0.2526039909,((((((((((291:0.7487242651
,292:0.7487242651):0.5923035821,290:1.341027847):0.4196063046,293:1.76063
4152):0.08469296414,289:1.845327116):0.3577326236,288:2.203059739):0.1780
337482,294:2.381093488):0.2740668179,((296:0.3054612818,297:0.3054612818)
:2.03275918,295:2.338220461):0.3169398441):0.1259222597,298:2.781082565):
0.1729866324,287:2.954069198):0.1822490093,286:3.136318207):3.437053327,(
(((301:0.1721104094,302:0.1721104094):3.73087884,303:3.90298925):0.648978
7831,300:4.551968033):0.7838944782,299:5.335862511):1.237509023):5.712235
695):7.931287439,222:20.21689467):11.55845331,(220:4.832074845,221:4.8320
74845):26.94327313):8.764898207):5.457014537,(((((217:2.267821081,218:2.2
67821081):1.131978486,219:3.399799567):1.983090952,216:5.382890519):0.403
1164188,((213:1.839338988,214:1.839338988):2.475727952,215:4.315066941):1
.470939997):18.66244164,(211:3.871128717,212:3.871128717):20.57731986):21
.54881214):4.748875494,(((207:0.5669609207,208:0.5669609207):0.2415669409
,206:0.8085278616):6.265394722,(209:1.612447616,210:1.612447616):5.461474
967):43.67221363):3.302556507,(((((201:3.893969257,202:3.893969257):5.290
690921,203:9.184660178):1.240842316,(204:4.659661852,205:4.659661852):5.7
65840642):14.44998501,((199:2.658010104,200:2.658010104):5.576660358,198:
8.234670462):16.64081704):4.710595457,(((((192:1.17722043,193:1.17722043)
:0.6695909841,194:1.846811414):0.7884868361,195:2.63529825):0.2630634533,
191:2.898361703):1.349129702,(196:0.7088259468,197:0.7088259468):3.538665
458):25.33859156):24.46260976):1.061043409,((((((((((((((((((((124:1.81
0224195,125:1.810224195):0.6981744799,126:2.508398675):0.3777015347,123:2
.88610021):1.361681964,122:4.247782173):0.91081529,121:5.158597463):1.282
798497,((118:0.9661037421,119:0.9661037421):1.236772649,120:2.202876391):
4.238519569):0.5664141509,117:7.007810111):1.176727519,(((115:1.502279383
,116:1.502279383):0.2510335191,114:1.753312902):5.941603883,113:7.6949167
85):0.4896208441):1.070778859,((((((111:0.5633379267,112:0.5633379267):0.
09224780225,110:0.6555857289):0.5377821851,109:1.193367914):1.446158705,1
08:2.639526619):1.116408127,107:3.755934746):2.159819064,((105:2.57005738
3,106:2.570057383):1.027141092,104:3.597198475):2.318555335):3.339562678)
:0.7854006137,103:10.0407171):0.3377186814,(((((((98:0.9216580227,99:0.92
16580227):1.109746374,100:2.031404397):0.729303191,97:2.760707588):2.2638
99018,96:5.024606606):1.125097198,95:6.149703804):0.9526181412,101:7.1023
21946):0.5846621107,102:7.686984056):2.691451727):0.3187955113,94:10.6972
3129):0.131546095,((91:1.872841208,92:1.872841208):0.8634375218,93:2.7362
78729):8.09249866):1.251543468,90:12.08032086):5.868018188,((((84:0.65048
26522,85:0.6504826522):5.61626203,86:6.266744683):4.942612833,((87:3.2611
61045,88:3.261161045):5.059896971,89:8.321058017):2.888299499):2.51541908
4,83:13.7247766):4.223562445):1.534103088,127:19.48244213):0.5146986004,(
((79:5.77871815,80:5.77871815):3.800476017,(81:4.048447681,82:4.048447681
):5.530746486):4.094701633,78:13.6738958):6.323244933):2.176349381,((((((
(59:2.257309162,60:2.257309162):0.7586950901,58:3.016004252):0.0937232931
8,57:3.109727545):0.8150408795,(55:0.9313242999,56:0.9313242999):2.993444

125):1.216261802,(53:5.009904904,54:5.009904904):0.1311253219):2.09642869
6,(((((64:0.7740060133,65:0.7740060133):0.5718513895,63:1.345857403):2.39
6286471,62:3.742143873):0.2991418422,66:4.041285715):1.901998615,61:5.943
28433):1.294174592):5.324456778,((((((((76:0.7667794035,77:0.7667794035):
0.8611194847,75:1.627898888):0.5116613449,74:2.139560233):0.3678010104,73
:2.507361244):0.2935062797,72:2.800867523):0.9336331591,((69:1.180051595,
70:1.180051595):0.9158228594,71:2.095874454):1.638626228):1.478958175,68:
5.213458857):2.498598087,67:7.712056945):4.849858756):9.611574414):0.7547
978189,(((((131:1.463545769,132:1.463545769):0.1848685465,133:1.648414315
):0.8968468386,130:2.545261154):0.5220761885,(128:0.1583251258,129:0.1583
251258):2.909012217):4.792754901,134:7.860092243):15.06819569):4.74872686
9,(((((((((46:0.9813024067,47:0.9813024067):0.2571640123,45:1.238466419)
:0.8396329745,44:2.078099394):0.4809593359,43:2.559058729):0.644367425,(4
8:2.491658219,49:2.491658219):0.7117679354):0.5417019699,42:3.745128124):
0.09048589334,41:3.835614018):0.476262244,((51:0.4503375641,52:0.45033756
41):1.258371836,50:1.7087094):2.603166862):2.28793183,(39:4.546353246,40:
4.546353246):2.053454845):1.087140219,(((37:2.365160717,38:2.365160717):1
.229918025,36:3.595078742):1.507510996,35:5.102589738):2.584358572):19.99
006649):5.549315718,(((((((((150:2.389909677,151:2.389909677):1.17280017
4,149:3.562709852):0.3327080661,148:3.895417918):0.8410132485,((146:0.893
3804393,147:0.8933804393):2.05086529,145:2.944245729):1.792185437):3.0310
73674,(((152:3.524680068,153:3.524680068):2.536608528,(154:5.218421643,15
5:5.218421643):0.8428669539):0.9182527745,156:6.979541371):0.7879634699):
5.206455542,((((141:1.658557818,142:1.658557818):2.725625596,143:4.384183
414):4.48394826,144:8.868131675):0.7367494561,140:9.604881131):3.36907925
1):2.502632731,((136:1.892401769,137:1.892401769):1.418541819,(138:2.4748
58823,139:2.474858823):0.8360847652):12.16564953):2.323846803,(((((157:1.
573462911,158:1.573462911):0.6611517489,159:2.23461466):0.1556295967,160:
2.390244257):7.224782333,(((162:1.863823109,163:1.863823109):2.405237256,
164:4.269060366):1.180401325,161:5.449461691):4.165564899):2.184740166,((
(166:7.229395067,167:7.229395067):1.045444251,165:8.274839318):1.37093540
8,(168:5.675010412,169:5.675010412):3.970764315):2.15399203):6.000673161)
:1.09706119,((((((((185:0.02966930808,186:0.02966930808):1.544865187,187:
1.574534495):1.408779237,188:2.983313732):0.4625153903,(183:0.447750377,1
84:0.447750377):2.998078745):0.6791992981,(((179:1.684691589,180:1.684691
589):0.4627419993,178:2.147433588):1.928987137,(181:3.341160399,182:3.341
160399):0.7352603264):0.0486076953):0.3377798312,(189:3.578644946,190:3.5
78644946):0.8841633054):0.457342589,(((((176:1.628097904,177:1.628097904)
:0.5838802906,175:2.211978195):0.4994727499,174:2.711450944):0.5926383619
,173:3.304089306):0.5946726136,172:3.89876192):1.02138892):10.47749543,(1
70:5.839481503,171:5.839481503):9.558164768):3.499854836):2.335808795,135
:21.2333099):11.99302062):18.72195696,((32:12.97705679,33:12.97705679):8.
935619372,34:21.91267616):30.03561133):3.161448644):6.465137503,((((((((
(((476:1.87770641,477:1.87770641):4.611517861,(478:1.520843643,479:1.5208
43643):4.968380628):2.985110415,480:9.474334685):5.145636434,481:14.61997
112):2.970785193,(((485:10.31764707,486:10.31764707):2.899090057,484:13.2
1673713):1.23547694,(482:10.28392078,483:10.28392078):4.168293282):3.1385
42245):0.5077677384,(((487:10.53623196,488:10.53623196):5.466877623,(489:
14.8721294,490:14.8721294):1.130980183):0.9762685023,((491:12.80687427,49
2:12.80687427):3.874949309,493:16.68182357):0.2975545103):1.119145966):3.
736752969,((((472:3.90138433,473:3.90138433):0.8521870857,471:4.753571416
):1.121304888,(474:0.6652500135,475:0.6652500135):5.209626291):4.30210311
2,470:10.17697942):11.6582976):8.971899707,494:30.80717673):1.604209177,(
(((((463:3.284417571,464:3.284417571):4.819147828,(465:5.905310299,466:5.
905310299):2.1982551):0.1814086027,462:8.284974002):0.6348878406,467:8.91
9861842):1.209987188,468:10.12984903):0.7447355208,469:10.87458455):21.53
680135):12.18052215,(((498:22.17912874,499:22.17912874):1.618131783,497:2
3.79726053):14.82793574,(495:12.13869155,496:12.13869155):26.48650473):5.

96671178):0.9998148011,((((((((((((((((((446:2.990826257,447:2.990826257)
:6.661528171,448:9.652354428):2.178635698,449:11.83099013):1.91730832,(45
0:5.156834386,451:5.156834386):8.591464059):1.226610471,452:14.97490892):
0.9765521918,445:15.95146111):1.751333713,444:17.70279482):1.859750278,((
(439:12.11518314,440:12.11518314):3.501905671,441:15.61708881):1.33257040
8,(442:10.20724281,443:10.20724281):6.742416404):2.612885885):0.457733551
9,((437:5.9511002,438:5.9511002):11.24379903,436:17.19489923):2.825379426
):0.1359845193,(((454:0.9971324389,455:0.9971324389):0.3661444912,453:1.3
6327693):11.54579113,456:12.90906807):7.247195105):2.416456676,435:22.572
71985):0.6192018679,((458:11.30522195,459:11.30522195):5.931546118,457:17
.23676807):5.955153646):1.956604648,(460:7.349476749,461:7.349476749):17.
79904961):6.111266913,434:31.25979327):1.241094071,433:32.50088735):5.542
273353,(431:37.74386163,432:37.74386163):0.2992990685):2.284003498,430:40
.3271642):4.460951485,429:44.78811568):0.8036071724):5.245940485,(((((((4
25:0.8438051024,426:0.8438051024):0.3851974615,427:1.229002564):16.296747
32,424:17.52574988):2.368975676,428:19.89472556):0.050735051,((421:3.6762
82575,422:3.676282575):10.12241914,423:13.79870172):6.146758894):2.350681
667,(419:3.837004256,420:3.837004256):18.45913802):20.95020537,(((411:8.3
88802334,412:8.388802334):25.74977303,(413:19.77797658,414:19.77797658):1
4.36059879):2.549917793,(((417:10.88961301,418:10.88961301):8.563190741,4
16:19.45280375):5.822557381,415:25.27536113):11.41313203):6.557854483):7.
591315697):10.73721029):4.738044794,(((((((27:6.821251439,28:6.821251439)
:20.95427398,(29:9.86629393,30:9.86629393):17.90923148):1.922730264,31:29
.69825568):9.485444436,(((25:11.0532105,26:11.0532105):2.372420853,24:13.
42563135):3.931274172,23:17.35690552):21.82679459):20.84635282,((((20:8.9
42317171,21:8.942317171):1.444429789,22:10.38674696):1.446689574,19:11.83
343653):30.56335483,(17:24.72535687,18:24.72535687):17.67143449):17.63326
157):3.432390846,((((((((12:2.279754479,13:2.279754479):1.987562407,14:4.
267316886):3.519604956,15:7.786921842):0.4720082513,(10:0.390519895,11:0.
390519895):7.868410198):2.685358305,9:10.9442884):11.21642916,16:22.16071
756):2.309703363,(7:12.21111458,8:12.21111458):12.25930634):2.712893608,6
:27.18331453):36.27912925):1.900357137,(4:1.148719074,5:1.148719074):64.2
1408184):0.9501175084):21.52526525):0.3934027931,((((((((((1038:19.332820
74,1039:19.33282074):1.391432048,(1040:17.96409218,1041:17.96409218):2.76
0160608):7.972296023,1042:28.69654882):5.103460271,((1043:16.16515544,104
4:16.16515544):5.952034875,(1045:4.120422914,1046:4.120422914):17.9967674
):11.68281878):16.62785534,1037:50.42786443):17.96585483,((((1049:8.69454
6421,1050:8.694546421):0.3314377733,1048:9.025984194):3.353243001,1051:12
.3792272):1.227623804,1047:13.606851):54.78686826):6.920563383,(((((1033:
5.660069618,1034:5.660069618):8.945788509,(1035:6.484933184,1036:6.484933
184):8.120924944):4.397321673,(1031:17.14052616,1032:17.14052616):1.86265
364):1.396396547,(1029:13.26819528,1030:13.26819528):7.131381067):29.2714
5075,((1026:2.07702116,1027:2.07702116):0.2845754757,1028:2.361596635):47
.30943046):25.64325554):1.473419738,1025:76.78770238):0.6061276625,(((((1
023:0.5918510516,1024:0.5918510516):6.428389881,1022:7.020240933):2.86421
2443,(1020:4.601491782,1021:4.601491782):5.282961594):39.82331222,(1018:3
.623614676,1019:3.623614676):46.08415092):3.391673078,((((1015:0.12371649
32,1016:0.1237164932):0.3211931813,1014:0.4449096745):8.066637909,1017:8.
511547583):1.242148442,1013:9.753696026):43.34574265):24.29439136):2.7717
51209,((((((1058:5.710420124,1059:5.710420124):0.9773501783,1057:6.687770
302):2.423073266,1056:9.110843568):14.98678342,(((1062:14.04505184,1063:1
4.04505184):0.7975041565,1061:14.842556):7.426744447,1060:22.26930045):1.
828326542):3.987520372,(((1054:1.033828248,1055:1.033828248):3.125383734,
1053:4.159211982):11.00999327,1052:15.16920526):12.91594211):27.9682,((((
1064:0.7638346988,1065:0.7638346988):5.95154894,1066:6.715383638):26.0349
6795,1067:32.75035159):13.60540042,((1068:3.365146347,1069:3.365146347):1
9.32115351,(1070:4.15686875,1071:4.15686875):18.5294311):23.66945216):9.6
97595354):24.11223388):8.066005221):68.88243058,(((((((((((((((((1121:0.0

6444800015,1122:0.06444800015):2.75679645,1120:2.821244451):0.3931723723,
(1123:2.486771556,1124:2.486771556):0.7276452669):1.174885921,1125:4.3893
02743):0.3357107591,((1117:0.8463765755,1118:0.8463765755):2.19150689,111
9:3.037883466):1.687130037):0.6752748356,(1115:1.95841204,1116:1.95841204
):3.441876298):3.708519882,((((1111:4.258107287,1112:4.258107287):2.15870
8726,(1113:1.01739183,1114:1.01739183):5.399424183):0.8538062334,(1109:2.
323740021,1110:2.323740021):4.946882225):0.7585950221,1108:8.029217269):1
.079590951):0.42015419,1126:9.52896241):7.23580266,(((1129:3.493002352,11
30:3.493002352):7.45572895,1131:10.9487313):2.604233532,(1127:6.893544497
,1128:6.893544497):6.659420337):3.211800237):7.39580051,1107:24.16056558)
:13.00381083,(((1134:1.071548687,1135:1.071548687):1.921293814,1136:2.992
842501):25.23645291,(1132:19.63595362,1133:19.63595362):8.593341789):8.93
5081005):0.7650825358,((((1105:4.446613602,1106:4.446613602):4.993169412,
1104:9.439783014):5.837336663,1103:15.27711968):18.06470069,1102:33.34182
036):4.587638586):4.983076202,((1100:4.628790836,1101:4.628790836):20.822
41661,1099:25.45120744):17.46132771):9.77474433,(((((((((1092:6.943547772,
1093:6.943547772):2.17653458,1091:9.120082352):3.945415008,(1094:8.633748
892,1095:8.633748892):4.431748467):5.196091636,1096:18.261589):1.88673900
3,1097:20.148328):9.757368874,1098:29.90569687):21.45187682,(((1087:3.252
943113,1088:3.252943113):3.648075454,(1089:3.040553429,1090:3.040553429):
3.860465138):19.32630253,1086:26.2273211):25.13025259):0.7332686948,(1084
:6.579845953,1085:6.579845953):45.51099643):0.5964370988):3.394260472,108
3:56.08153995):12.18908028,((((((((1079:2.348031726,1080:2.348031726):2.7
48160268,1078:5.096191994):5.920761796,1081:11.01695379):7.774662766,1077
:18.79161656):8.668955292,1076:27.46057185):2.35046066,1075:29.81103251):
3.364366655,1074:33.17539916):1.644463016,1082:34.81986218):33.45075805):
5.165559667,(1072:16.32065977,1073:16.32065977):57.11552013):83.67783715)
:24.89488856,((1:11.50274145,2:11.50274145):33.46755395,3:44.97029541):13
7.0386102);
END;
