# Supplementary material for: Divergent vertebral formulae shape the evolution of axial complexity in mammals
Source: Nat Ecol Evol. 2023 Mar 6;7(3):367–81. doi: 10.1038/s41559-023-01982-5 (PMC9998275; doi:10.1038/s41559-023-01982-5)
Supplement: Supplementary file 7 — R codes. [file 41559_2023_1982_MOESM7_ESM.pdf]

R codes

This document details the steps required to carry out the analyses and to reproduce the results and graphics presented in this work. The time-calibrated phylogeny, available as an object of class ‘phylo’ in Supplementary Data 3, can be opened in WORDS and saved as a text file with the suffix ‘.nex’. Below, we call such phylo object ‘phy_reordered.nex’. For convenience, the data used to build the figures are provided as separate EXCEL sheets in the Source Data files (see also Supplementary Data 1). For figures that include several panels, we select an example panel to illustrate the application of the code. In the following text, the explanatory notes preceding or following each line of code are marked by the # symbol.

##
Code for reproducing **Fig. 2 | Time-calibrated phylogeny of 1,136 extant mammal species used in this study.**
##

###Loading ape, ggtree, and tidytree packages

```
require(ape); require(ggtree); require(tidytree)
```

###Importing time-calibrated phylogeny

```
tree<-read.nexus("phy_reordered.nex")
```

###Distributing species into ten major groups, after identification of the latest common ancestral node subtending each group ('getMRCA' function in ape); the newly created tree file, with information on taxon allocation to each group, is stored as a separate object 'tree2' to avoid overwriting the previous tree file, if the latter is needed

```
tree2<-
groupClade(tree,c(1875,2033,1861,1657,1528,1142,2270,2206,2187,2
149,2121))
```

###To identify the node number corresponding to the latest common ancestor of each group (numbers within brackets in the line of code above), we select two species in that group that are sufficiently separate on the tree to bracket all other intervening species; for instance, to identify the node subtending all Cetartiodactyla (node '1657'), one could use the wild Bactrian camel (*Camelus ferus*) and the mouflon (*Ovis orientalis*) as bracketing taxa, as follows

```
getMRCA(tree,tip=c("Camelus_ferus","Ovis_orientalis"))
```

###Assigning colours manually to groups, converting tree into circular cladogram stored as separate object 'p' (to avoid overwriting previous tree files, if the latter are needed), and plotting cladogram with colour-coded branches and without taxon names

```
p<-
ggtree(tree2,layout='circular',size=0.2,aes(color=group))+theme(
legend.position='none')+scale_color_manual(values=c("black","ora
nge","forestgreen","magenta","dodgerblue","pink","yellow2","maro
on","maroon","darkgrey","cyan2","chartreuse"))

plot(p)
```

###Note: 'maroon' was assigned to both Monotremata and Marsupialia as these clades were grouped together to accommodate the small number of monotreme species in our sample; 'black' was assigned to the basal tree branches; the plot thus obtained was saved as an image file, opened in Powerpoint, and flipped upside down; silhouettes of representative mammal species (from phylopic.org) were added to the circular cladogram in Powerpoint

##
Code for reproducing **Fig. 3 | Violin plots.**
##

###Loading `vioplot` package

```
require(vioplot)
```

###Importing dataset with species names as row names, numeric values of index variable in first column, and factor levels of grouping variable (consisting of names of major mammal groups) in second column; we use the Brillouin index of the thoracolumbar region as an example
###File Ruta_SourceData_Fig3.xlsx – SHEET 3 (extract tabulation and save it as '.txt' file, e.g., data_Brillouin_index_thoracolumbar_region_reordered_with_groups.txt)

```
data<-
read.table("data_Brillouin_index_thoracolumbar_region_reordered_
with_groups.txt",header=T,row.names=1)
```

###Extracting index variable and grouping variable and linking them through simple linear formula

```
v<-data[,1]

g<-data[,2]

f<-v~g
```

###Building violin plots colour-coded by groups and with group names indicated by three-letter abbreviations, showing median values of indices as white circles and interquartile ranges as solid black vertical bars

```
vioplot(f,col=c("cyan2","dodgerblue","forestgreen","chartreuse",
"yellow2","maroon","magenta","orange","pink","darkgrey"),border=
"black",lwd=0.4,pchMed=21,colMed="black",colMed2="white",cex=1.2
,lineCol="black",rectCol="black",names=c("Afr","Cet","Chi","Eul"
,"Gli","MoMa","Per","Fer","Eua","Xen"),areaEqual=F,xlab="group",
ylab=expression(paste(H[R],"TL")))
```

###Note: the procedure outlined above can be repeated for each variable of interest, after assigning different names to the various datasets, their respective index and grouping variables, and the linear formulae (e.g., 'data1', 'data2', 'data3', … , etc.; 'v1', 'v2', 'v3', … , etc.; 'g1', 'g2', 'g3', … , etc.; '`f1<-v1~g1`', '`f2<-v2~g2`', '`f3<-v3~g3`', … , etc.); to assemble violin plots for different indices in a single figure, we open a blank window in which three rows and two columns are designed to accommodate all violin plots; we then repeat the command for generating violin plots for each dataset

```
par(mfrow=c(3,2))

vioplot(f1, …, etc.)
```

```
vioplot(f2, …, etc.)

vioplot(f3, …, etc.)
```

###The plot thus obtained was saved as an image file and opened in Powerpoint, where silhouettes of representative mammal species were added by hand

##
Code for reproducing **Fig. 4 | Continuous mapping of complexity indices across the phylogeny.**
##

###Note: the code is also applicable to Extended Data Fig. 1

###Loading `phytools` and `viridis` packages

```
require(phytools); require(viridis)
```

###Importing time-calibrated phylogeny, if not stored already

```
tree<-read.nexus("phy_reordered.nex")
```

###Importing dataset with species names as row names and numeric values of index variable in first column; we use the Brillouin index of the thoracolumbar region as an example
###File Ruta_SourceData_Fig4.xlsx – SHEET 2 (extract tabulation and save it as '.txt' file, e.g., data_Brillouin_index_thoracolumbar_region_reordered.txt)

```
data<-
as.matrix(read.table("data_Brillouin_index_thoracolumbar_region_
reordered.txt",header=T,row.names=1))
```

###Extracting variable of interest

```
trait<-data[,1]
```

###Creating continuous trait mapping object without plotting it

```
ctm<-contMap(tree,trait,plot=FALSE,res=1000)
```

###Setting 'turbo' colour mapping scale, with higher and lower states for trait represented by warmer and cooler colour tones, respectively

```
ctp<-setMap(ctm,turbo(1000,direction=1))
```

###Building circular cladogram with colour-coded distributions of interpolated trait changes across branches and maximum likelihood estimates of trait values at internal nodes

```
plot(ctp,fsize=c(0.0000000001,0.7),res=1000,turbo(1000),type="fa
n",lwd=0.65,outline=F,leg.txt="Brillouin TL")
```

###The lines of code above can be adapted to produce continuous trait maps for other variables, change the colour scale and branch tickness, and alter the size of the text (e.g., taxon names; scale bar legend)

###
Code for reproducing **Fig. 5 | Patterns of thoracolumbar differentiation across major groups.**
###

###Importing dataset with thoracolumbar counts in first column, thoracic:lumbar ratios in second column, and grouping variable in third column, and storing it as a data fame object
###File Ruta_SourceData_Fig5.xlsx – SHEET 1 (extract tabulation and save it as '.txt' file, e.g., data_thoracolumbar_counts_vs_thoracic_lumbar_ratios_reordered_with_groups.txt)

```
dataframe<-
as.data.frame(read.table("data_thoracolumbar_counts_vs_thoracic_
lumbar_ratios_reordered_with_groups.txt",header=T,row.names=1))
```

###Extracting variables of interest

```
g<-dataframe$group

TLR<-dataframe$TL_ratio

TLS<-dataframe$Thoracolumbar_counts
```

###Preparing background colours to be assigned to plotting symbols representing species in each major group (one symbol per group); first, we create a string of identical colours (say, 'grey') for the 1136 species in the data

```
bg<-rep("grey",1136)
```

###Identifying position of species in each major group within 'group' variable and creating vector for said positions; for instance, in the case of Afrotheria, the vector can be extracted from the 'group' variable as follows

```
afr<-as.vector(which(g=="Afrotheria"))
```

###Selecting colour for elements of 'afr' vector, such that corresponding elements in original 'bg' string are coloured accordingly

```
bg[afr]<-'cyan2'
```

###Repeating procedure for all other groups

```
cet<-as.vector(which(g=="Cetartiodactyla"))
bg[cet]<-'dodgerblue'
```

```
chi<-as.vector(which(g=="Chiroptera"))
bg[chi]<-'forestgreen'

eul<-as.vector(which(g=="Eulipotyphla"))
bg[eul]<-'chartreuse'

gli<-as.vector(which(g=="Glires"))
bg[gli]<-'yellow2'

moma<-as.vector(which(g=="MonotremataMarsupialia"))
bg[moma]<-'maroon'

per<-as.vector(which(g=="Perissodactyla"))
bg[per]<-'magenta'

fer<-as.vector(which(g=="PholidotaCarnivora"))

bg[fer]<-'orange'

eua<-as.vector(which(g=="ScandentiaPrimates"))
bg[eua]<-'pink'

xen<-as.vector(which(g=="Xenarthra"))
bg[xen]<-'darkgrey'
```

###Preparing plotting symbols to be assigned to species in each major group; first, we create a string of identical symbols (say, '21', a filled circle) for the 1136 species in the data

```
pch<-rep("21",1136)
```

###Selecting plotting symbol for each major group, using each symbol twice for different groups, but with alternative colours

```
pch[afr]<-'21'     #filled circle
pch[cet]<-'22'     #filled square
pch[chi]<-'23'     #filled diamond
pch[eul]<-'24'     #filled equilateral triangle with one vertex pointing up
pch[gli]<-'25'     #filled equilateral triangle with one vertex pointing down
pch[moma]<-'21'    #filled circle
pch[per]<-'22'     #filled square
pch[fer]<-'23'     #filled diamond
pch[eua]<-'24'     #filled equilateral triangle with one vertex pointing up
pch[xen]<-'25'     #filled equilateral triangle with one vertex pointing down
```

###Converting 'pch' string to numerical values

```
pch<-as.numeric(pch)
```

###Building local polynomial regression fitting model (loess), using thoracolumbar counts as predictor variable and thoracic:lumbar ratios as response variable

```
lwt<-loess(TLR~TLS,data=dataframe)
```

###Building bivariate scatterplot of thoracic:lumbar ratios vs. thoracolumbar counts

```
plot(TLR~TLS,data=dataframe,pch=pch,cex=1.3,lwd=0.3,col="black",
bg=bg,ylab="thoracic:lumbar ratio",xlab="thoracolumbar count")
```

###Reordering values of thoracolumbar counts and storing them as variable 'j'

```
j<-order(TLS)
```

###Superimposing loess regression line from fitting model

```
lines(TLS[j],lwt$fitted[j],col="brown",lwd=0.85)
```

###Adding legend

```
legend("topright",legend=c("Afrotheria","Cetartiodactyla","Chiro
ptera","Eulipotyphla","Glires","Monotremata +
Marsupialia","Perissodactyla","Ferae","Euarchonta","Xenarthra"),
pch=c(21,22,23,24,25,21,22,23,24,25),pt.cex=1.2,pt.bg=c("cyan2",
"dodgerblue","forestgreen","chartreuse","yellow2","maroon","mage
nta","orange","pink","darkgrey"),pt.lwd=0.3,cex=1,box.lty=0)
```

###The lines of code above can be adapted to produce bivariate scatterplots of thoracic:lumbar ratios vs. each of the thoracic and lumbar counts

##
Code for reproducing **Fig. 6 | Bivariate plots of node estimates of complexity indices vs. node ages and descendant-ancestor differences vs. node estimates.**
##

###Note: the code is also applicable to Extended Data Fig. 2 and Supplementary Figs 8–19

###Loading `MASS` package

```
require(MASS)
```

###Importing dataset with ancestral estimates of index variable in first column, node ages in second column, descendant-ancestor differences corrected for regression to the mean in third column, and factor levels of grouping variable in fourth column; we use the Brillouin index of the thoracolumbar region as an example
###File Ruta_SourceData_Fig6.xlsx – SHEET 1 (extract tabulation and save it as '.txt' file, e.g., data_ancestor_value_node_age_descendant_ancestor_difference_Brillouin_index_thoracolumbar_region.txt)

```
table<-
read.table("data_ancestor_value_node_age_descendant_ancestor_dif
ference_Brillouin_index_thoracolumbar_region.txt",header=T)
```

###Extracting variables of interest

```
ancestor<-table$ancestor_Brillouin_TL

age<-table$ancestor_age

D1<-table$ancestor_descendant_difference
```

`D1=-D1` ###Changing sign of difference, such that negative and positive values represent, respectively, decreases and increases

```
g<-table$group
```

###Preparing background colours to be assigned to plotting symbols representing species in each major group; first, we create a string of identical colours (say, 'black') for the 2270 internal nodes of the phylogeny

```
bg<-rep("black",2270)
```

###Identifying internal nodes belonging to each major group within 'group' variable and creating vector for those nodes; for instance, in the case of Afrotheria, the vector can be extracted from the 'group' variable as follows

```
afr<-as.vector(which(g=="Afrotheria"))
```

###Selecting colour for elements of 'afr' vector, such that corresponding elements in original 'bg' string are coloured accordingly

```
bg[afr]<-'cyan2'
```

###Repeating procedure for all other groups

```
cet<-as.vector(which(g=="Cetartiodactyla"))
bg[cet]<-'dodgerblue'

chi<-as.vector(which(g=="Chiroptera"))
bg[chi]<-'forestgreen'

eul<-as.vector(which(g=="Eulipotyphla"))
bg[eul]<-'chartreuse'

gli<-as.vector(which(g=="Glires"))
bg[gli]<-'yellow2'

moma<-as.vector(which(g=="MonotremataMarsupialia"))
bg[moma]<-'maroon'
```

```
per<-as.vector(which(g=="Perissodactyla"))
bg[per]<-'magenta'

fer<-as.vector(which(g=="PholidotaCarnivora"))
bg[fer]<-'orange'

eua<-as.vector(which(g=="ScandentiaPrimates"))
bg[eua]<-'pink'

xen<-as.vector(which(g=="Xenarthra"))
bg[xen]<-'darkgrey'

stem<-as.vector(which(g=="STEM"))
bg[stem]<-'black'
```

The category labelled as 'stem' refers to the basal tree branches, subtending the phylogenetic separation between major mammal cohorts

###Preparing plotting symbols to be assigned to internal nodes in each major group; first, we create a string of identical symbols (say, '21', a filled circle) for the 2270 internal nodes of the phylogeny

```
pch<-rep("21",2270)
```

###Selecting plotting symbol for each major group; each symbol is used twice for different groups, but with alternative colours

```
pch[afr]<-'21'
pch[cet]<-'22'
pch[chi]<-'23'
pch[eul]<-'24'
pch[gli]<-'25'
pch[moma]<-'21'
pch[per]<-'22'
pch[fer]<-'23'
pch[eua]<-'24'
pch[xen]<-'25'
pch[stem]<-'16'
```

###Converting 'pch' string to numerical values

```
pch<-as.numeric(pch)
```

###Building robust linear regression fitting model, using node ages as predictor variable and ancestral estimates of index as response variable

```
rlm<-rlm(ancestor~age)
```

###Building bivariate scatterplot of ancestral estimates vs. node ages

```
plot(age,ancestor,pch=pch,cex=1.3,bg=bg,lwd=0.3,xlab=expression(
paste("node  age")),ylab=expression(paste("ancestral  ", H[R] ,
"TL")))
```

###Superimposing solid regression line from fitting model

```
abline(rlm,lty=1,lwd= 0.85,col="brown")
```

###Building robust linear regression fitting model, using ancestral estimates as predictor variable and descendant-ancestor differences as response variable

```
rlm1<-rlm(D1~ancestor)
```

###Building bivariate scatterplot of descendant-ancestor differences vs. ancestral estimates

```
plot(ancestor,D1,pch=pch,cex=1.3,bg=bg,lwd=0.3,xlab=expression(p
aste("ancestral       ",      H[R]      ,      "TL")),
ylab=expression(paste("corrected ",Delta, H[R] , "TL")))
```

###Superimposing solid regression line from fitting model and dashed horizontal line separating positive (increases) and negative (decreases) values of response variable

```
abline(rlm1,lty=1,lwd= 0.85,col="brown")

abline(h=0,lty=3,lwd= 0.85,col="black")
```

###Outputting results of robust linear regression models

```
summary(rlm)

summary(rlm1)
```

###Note: the main output includes slope and intercept and their associated standard deviations and t statistic values; additional outputs (e.g., residuals) are the same as those obtained with the ‘lm’ function in base R (`stats` package); to test whether slope and intercept differ significantly from zero, the following procedure is applied to the ‘rlm’ and ‘rlm1’ models

###Loading `sfsmisc` package

```
require(sfsmisc)
```

###Applying robust Wald F-test to slopes from ‘rlm’ and ‘rlm1’ models

```
f.robftest(rlm,var=-1)

f.robftest(rlm1,var=-1)
```

###Applying robust Wald F-test to intercepts from the same models

```
f.robftest(rlm,var=1)

f.robftest(rlm1,var=1)
```

###Note: to obtain the robust linear regression models and associated statistics for the individual groups, as shown in Supplementary Table 4, we subset the original 'table' object; it is convenient to have it converted into a data frame first

```
df<-as.data.frame(table)
```

###As an example, to gather all data relevant to Cetartiodactyla, we enter the following

###Extracting ancestral node estimates

```
anccet<-df$ancestor_Brillouin_TL[df$group=="Cetartiodactyla"]
```

###Extracting node ages

```
agecet<-df$ancestor_age[df$group=="Cetartiodactyla"]
```

###Extracting descendant-ancestor differences

```
D1cet<-
df$ancestor_descendant_difference[df$group=="Cetartiodactyla"]
```

`D1cet=-D1cet` ###Changing sign of difference, such that negative and positive values represent, respectively, decreases and increases

###Building robust linear regression models for Cetartiodactyla; each model can be plotted and inspected further with the `summary()` and `f.robftest()` functions

```
rlmcet<-rlm(anccet~agecet)

rlmcet1<-rlm(D1cet~anccet)
```

###Note: once the robust linear regression models for all groups are built, their respective regression lines can be superimposed onto the colour-coded scatterplots by calling the `abline()` command once for each group

##
Code for reproducing **Extended Data Fig. 1 | Continuous mapping of complexity indices across the phylogeny.**
##

###See code for reproducing Fig. 4 above

##
Code for reproducing **Extended Data Fig. 2 | Bivariate plots of node estimates of complexity indices vs. node ages and descendant-ancestor differences vs. node estimates.**
##

###See code for reproducing Fig. 6 above

##
Code for reproducing **Extended Data Fig. 3 | Subclade tests in selected mammal groups.**
##

###Note: the code is also applicable to Supplementary Figs 20–24

code for the Analysis of Skewness written by Professor Steve C. Wang
Department of Mathematics and Statistics, Swarthmore College
500 College Ave, Swarthmore, PA 19081 USA
reference: Wang, S. C. Quantifying passive and driven large-scale evolutionary trends. *Evolution* **55**(5), 849–858 (2001).

```
# -----------------------------------------------------------------#
#
# revised version, Jan 2004. Adds degrees of freedom and "F stat"
#
# -----------------------------------------------------------------#


anskew <- function(data, N, ngroups, hvalue=.1, showplot=1,
                  showtable=1)
{
  # The Analysis of Skewness
  # S-plus or R code by Steve C. Wang, revised Jan 2004
  # ref: Wang (2001), _Evolution_ 55:5.
  #
  # data: Nx2 matrix: values in column 1,
  # group membership in column 2, numbered 1...ngroups
  # N = total number of observations
  # ngroups = number of groups
  # showplot: 1 = plot shown, 0 = no plot shown
  # hvalue: bandwidth for density estimate in plot

  # Needs function 'dens' (included below) to make plots


  # Initialize
  n <- ybari <- ss <- vars <- sz <- skews <- rep(0, ngroups)
```

```
  # Determine number of values in each group
  y <- data[,1]                 # data values
  group <- data[,2]            # group memberships
  for (i in 1:N)              # n = vector giving number in each
group
    n[group[i]] <- n[group[i]] + 1


  # Calculate within-group sum of cubes
  for (i in 1:ngroups)
  {
    yi <- y[group==i]           # vector of values for group i
    ybari[i] <- mean(yi)        # vector of group means
    ss[i] <- (n[i]-1)*var(yi)   # sum of squares of values for
group i
    sz[i] <- sum( (yi - rep(ybari[i],n[i])) )     # used only for
check
    skews[i] <- sum((yi - rep(ybari[i],n[i]))^3)  # sum of cubes
for group i
  }
  scw <- sum(skews)              # sum of cubes summed over all
groups


  # Calculate overall statistics
  ybar <- mean(y)               # overall mean
  sst <- (N-1)*var(y)              # overall sums of squares of
values
  sct <- sum( (y - rep(ybar, N))^3 )      # total sum of cubes


  # Calculate between-group sum of cubes
  scb <- sum( (ybari - rep(ybar,ngroups))^3 * n )


  # Calculate heteroskedasticity sum of cubes
  sch <- 3*sum( (ybari - rep(ybar,ngroups)) * ss )


  # Calculate zero cross term (just as a check)
  scz <- 3*sum( (ybari - rep(ybar,ngroups))^2 * sz )


  # Error check
  # First, check that the extra cross term (SCZ) is zero.
  # Second, check that SCT = SCB + SCW + SCH.
  # If either check fails, print an error message.
  epsilon <- abs(ybar/1000000)           # numerical tolerance
  if (abs(scz) > epsilon)                # check that cross term
is zero
```

```
    cat("\n *** Warning: SCZ =", round(scz,9), "; should be zero
\n\n")
  if (abs(sct-scb-scw-sch) > epsilon)    # check that  SCT = SCB
+ SCW + SCH
    cat("\n *** Warning: SCT =", round(sct,9),
        "; should equal", round(scb+scw+sch,9), " \n\n")


  # Calculate degrees of freedom
  dfb <- ngroups - 2
  dfh <- ngroups
  dfw <- length(y) - 2*ngroups


  # Output
  if(showtable)
  {
    cat(" \n The Analysis of Skewness \n\n")
    cat(" source \t SC \t\t % \t\t df \t MC \t ratio \n");
    cat("     -----------------------------------------------------
\n");
    cat("        SCB\t\t",        signif(scb,4),        "\t");
cat(round(100*scb/sct,1), "%\t");
    cat(dfb, "\t");    cat(signif(scb/dfb,3), "\t");
    cat(round((scb/dfb)/(scw/dfw),2), "\n");
    cat("        SCH\t\t",        signif(sch,4),        "\t");
cat(round(100*sch/sct,1), "%\t");
    cat(dfh, "\t");    cat(signif(sch/dfh,3), "\t");
    cat(round((sch/dfh)/(scw/dfw),2), "\n");
    cat("        SCW\t\t",        signif(scw,4),        "\t");
cat(round(100*scw/sct,1), "%\t");
    cat(dfw, "\t");    cat(round(scw/dfw,2), "\n");
    cat("     -----------------------------------------------------
\n");
    cat(" SCT\t\t", signif(sct,4), "\n\n")
  }


  # Graphics
  if(showplot)
  {
    # Plot graph frame
    #par(mfrow=c(1,1), lty=1)
    yd <- dens(y, h=hvalue)      # density estimate for overall
distribution
    plot( yd, type="n", ylim=c(-.03,max(yd[,2]*1.1)), xlab="",
ylab="" )
    abline(0,0)
    title(main="Title goes here");      mtext("additional text
here")
    # Plot heavy black curve for overall distribution
```

```
    lines(yd, lwd=4)
    # Plot light colored curves for each group
    # Following three parameters control appearance; adjust as
necessary
    scale1 <- 10;    scale2 <- 99;    offset <- .005
    for (i in 1:ngroups)
    {
      ydi <- dens(y[group==i], h=hvalue)
      lines(ydi[,1],   ydi[,2]/scale1+(ngroups-i)/scale2-offset,
lwd=2, col=i+1)
      points(mean(y[group==i]), -.03, pch=16, cex=1.5, col=i+1)
# group mean
      points(ybar, -.03, pch="|", cex=1.7, col=1)              #
overall mean
      text(1.2, -.03, "group means", cex=.9)
    }
  }    # end if showplot

return(c(scb, sch, scw))
}    # end function anskew




# --------------------------------------------------------------- #

dens <- function(x, h=.10, npt=200, plot=0)
#      Kernel      density      estimate;      adapted      from
http://www.stat.sc.edu/rsrch/gasp/
# Input: x values of dataset
# Output: density estimate y-values ordered by x
{
  r <- max(x) - min(x)
  xmax <- max(x) + .1*r
  xmin <- min(x) - .1*r
  n <- length(x)
  xgrid <- seq(from=xmin, to=xmax, length=npt)
   y <- NULL
  for (i in 1:npt)
  {
    hump <- dnorm( (rep(xgrid[i],n)-x)/h )
    y <- c(y, sum(hump)/(n*h))
  }
  if(plot)   plot(xgrid, y, type="l", ylab=" ", xlab=" ")
  cbind(xgrid,y)
}
# --------------------------------------------------------------- #
```

###To run the subclade test, copy and paste the code provided above into a blank R window, then follow the lines of code below

###Importing dataset with species names as row names, numeric values of index variable in first column, and grouping variable (subclades within group) in second column; we use the Brillouin index of the thoracolumbar region in Euarchonta as an example
###File Ruta_SourceData_Fig3.xlsx – SHEET 1 (extract tabulation and save it as '.txt' file, e.g., data_Brillouin_index_thoracolumbar_region_Euarchonta.txt)

```
data<-
read.table("data_Brillouin_index_thoracolumbar_region_Euarchonta
.txt ",header=T,row.names=1)
```

###Checking skewness sign of index variable after loading `e1071` package

```
require(e1071)

skewness(data[,1])
```

###As the index variable is left-skewed, we transform it by taking the negative logarithm of its values

```
v=-log(data[,1])
```

###Transforming grouping variable by replacing group names with numbers

`g<-as.numeric(as.factor(data[,2]))` ###Useful if your grouping variable consists of names; otherwise, simply type

```
g<-data[,2]
```

###Binding transformed index variable with newly obtained grouping variable, using the same name for the dataset for simplicity

```
data<-cbind(v,g)
```

###Reordering data by values of grouping variable

```
data<-data[order(g),]
```

###Assigning names to data columns

```
colnames(data)=c("v","g")
```

###Specifying number of taxa in first column

```
N=126
```

###Specifying number of groups in second column

```
ngroups=3
```

###Running test

```
anskew(data,N,ngroups,hvalue=.1,showplot=1,showtable=1)
```

#The code will output a plot showing the probability density distributions of the entire group and of its constituent subclades, as well as a table of results where the total skewness is partitioned into within-group, between-group, and heteroscedasticity-related percentage components

##
Code for reproducing **Extended Data Fig. 4 | Shifts in rates of complexity change for the thoracolumbar region.**
##

###Note: the code is also applicable to Extended Data Fig. 5

###Loading `geiger` package

```
require(geiger)
```

###Importing time-calibrated phylogeny, if not stored already

```
tree<-read.nexus("phy_reordered.nex")
```

###Importing dataset with species names as row names and numeric values of the index variable in the first column; we use the Brillouin index of the thoracolumbar region as an example
###File Ruta_SourceData_Fig4.xlsx – SHEET 2 (extract tabulation and save it as '.txt' file, e.g., data_Brillouin_index_thoracolumbar_region_reordered.txt)

```
data<-
read.table("data_Brillouin_index_thoracolumbar_region_reordered.
txt",header=T,row.names=1)
```

###Extracting the index variable

```
dat<-data[,1]
```

###Creating directory for storing results from Bayesian sampler of evolutionary rates

```
r<-paste(sample(letters,9,replace=TRUE),collapse="")
```

###Starting reversible-jump Markov chain Monte Carlo sampling of rates under relaxed-clock Brownian motion ('rbm') model of evolutionary change

```
rjmcmc.bm(phy,dat,prop.width=1.5,ngen=50000000,samp=500,filebase
=r,simple.start=TRUE,type="rbm")
```

###Note: many sampling generations are recommended; this part of the calculations will take some considerable time

###Producing directory with results from the run

```
outdir<-paste("relaxedBM",r,sep=".")
```

###Loading results of posterior rate sampling

```
ps<-load.rjmcmc(outdir)
```

###Plotting phylogeny with branches coloured according to posterior rate estimates and with superimposed circles representing location and posterior probability of rate shifts

```
plot(x=ps,par="shifts",burnin=0.25,legend=T,show.tip=T,edge.widt
h=0.75,cex=0.1,label.offset=0.5,type="fan")
```

###The legends that accompany the plots are interpreted as follows. Grey branches exhibit background rates. Maroon and steelblue branches exhibit rates that are, respectively, higher and lower than the background rates. Colour intensity is proportional to the rate values, with darker tones indicating a greater difference between background and non-background rates. The circles mark the locations of rate shifts. Circle sizes are drawn in proportion to the posterior Bayesian probability of shifts. Circle colours represent shift magnitude, with darker maroon (respectively, steelblue) tone indicating a shift of greater magnitude towards a rate increase (respectively, decrease) relative to the rates of adjacent branches

##

Code for reproducing **Extended Data Fig. 5 | Shifts in rates of complexity change for the thoracolumbar region.**

##

###See code for reproducing Extended Data Fig. 4 above

##

Code for reproducing **Supplementary Data 1 | Probability density distributions of six complexity indices colour-coded by major group.**

##

###Loading `ggplot2` package

```
require(ggplot2)
```

###Importing dataset with species names as row names, numeric values of index variable in first column, and factor levels of grouping variable (consisting of names of major mammal groups, as used in the main text) in second column; we use the Brillouin index of the thoracolumbar region as an example

###File Ruta_SourceData_Fig3.xlsx – SHEET 3 (extract tabulation and save it as '.txt' file, e.g., data_Brillouin_index_thoracolumbar_region_reordered_with_groups.txt)

```
data<-
read.table("data_Brillouin_index_thoracolumbar_region_reordered_
with_groups.txt",header=T,row.names=1)
```

###Extracting index and grouping variables

```
v<-data[,1]

g<-data[,2]
```

###Checking range of index values to ensure that the interval for such index in the plotting window (see `xlim` argument in plotting code below) is sufficiently large and that the left and right tails of the density distributions are not truncated (or, at least, not excessively)

```
min(v); max (v)

[1] 0.2706139   ###Retrieved minimum value for index
[1] 0.6298005   ###Retrieved maximum value for index
```

###Creating vector of colours to be assigned to individual groups

```
cols<-
c("cyan2","dodgerblue","forestgreen","chartreuse","yellow2","mar
oon","magenta","orange","pink","darkgrey")
```

###Building plotting object, herewith termed 'p'

```
p<-
ggplot(data,aes(x=v,fill=g))+geom_density(alpha=0.5,lty=1,lwd=0.
25)+scale_fill_manual(values=cols,labels=c("Afrotheria","Cetarti
odactyla","Chiroptera","Eulipotyphla","Glires","Monotremata    +
Marsupialia","Perissodactyla","Ferae","Euarchonta","Xenarthra"))
+xlim(0.1,0.7)
```

###Plotting colour-coded probability density distributions for the index with label for the horizontal axis

```
p+xlab(expression(paste(H[R] , "TL")))
```

###
Code for reproducing **Supplementary Table 1 | Poisson regression analyses.**
###

###Loading `MASS` package

```
require(MASS)
```

###Importing dataset with index variable in first column and factor levels of grouping variable in third column; we use thoracolumbar counts and thoracic:lumbar ratios as an example
###File Ruta_SourceData_Fig5.xlsx – SHEET 1 (extract tabulation and save it as '.txt' file, e.g., data_thoracolumbar_counts_vs_thoracic_lumbar_ratios_reordered_with_groups.txt)

```
data<-
read.table("data_thoracolumbar_counts_vs_thoracic_lumbar_ratios_
reordered_with_groups.txt",header=T,row.names=1)
```

###Extracting variables of interest

```
TLS<-data$Thoracolumbar_counts

g<-data$group
```

###Building Poisson model with thoracolumbar counts as a function of groups

```
model.p = glm(TLS~g,data=data,family="poisson")
```

###Outputting results of Poisson model

```
summary(model.p)
```

###Loading `car` package

```
require(car)
```

###Running Analysis of Deviance on Poisson model) to assess degree and significance of parameter deviance from null model

```
Anova(model.p,type="II",test="LR")
```

###Loading `rcompanion` package

```
require(rcompanion)
```

###Calculating pseudo-R^2 coefficients to measure how well the Poisson regression model explains the data

```
nagelkerke(model.p)
```

###Loading `multcompView` and `emmeans` packages

```
require(multcompView); require(emmeans)
```

###Conducting post-hoc tests of significant pair-wise differences between estimated group-specific count means with significance adjustment for multiple comparisons

```
marginal = emmeans(model.p,~g)

pairs(marginal,adjust="tukey")
```

###
Code for reproducing **Supplementary Table 2 | Phylogenetic analyses of variance.**
###

###Loading `phytools` package

```
require(phytools)
```

###Importing time-calibrated phylogeny, if not stored already

```
tree<-read.nexus("phy_reordered.nex")
```

###Importing dataset with species names as row names, numeric values of index variable in first column, and factor levels of grouping variable (consisting of names of major mammal groups) in second column; we use the Brillouin index of the thoracolumbar region as an example
###File Ruta_SourceData_Fig3.xlsx – SHEET 3 (extract tabulation and save it as '.txt' file, e.g., data_Brillouin_index_thoracolumbar_region_reordered_with_groups.txt)

```
data<-
read.table("data_Brillouin_index_thoracolumbar_region_reordered_
with_groups.txt",header=T,row.names=1)
```

###Transforming grouping variable by replacing group names with numbers

```
g<-as.numeric(as.factor(data[,2]))
```

###Extracting index variable

```
v<-as.vector(data[,1])
```

###Performing phylogenetic analysis of variance through simulations, conducting post-hoc tests of significant pair-wise differences between group-specific index means, and adjusting significance level through false discovery rate procedure for multiple comparisons

```
phylANOVA(tree,g,v,nsim=1000,posthoc=TRUE,p.adj="fdr")
```

###
Code for reproducing **Supplementary Table 3 | Phylogenetically corrected correlations between vertebral counts and complexity indices.**
###

###Loading `caper` package

```
require(caper)
```

###Importing time-calibrated phylogeny, if not stored already

```
tree<-read.nexus("phy_reordered.nex")
```

###Brunch method – Build dataset file (e.g., dataset.txt) with species names in first column, vertebral counts in second column, and complexity index in third column; in this example, we correlate thoracolumbar counts with the Brillouin index of the thoracolumbar region

```
dataframe<-as.data.frame(read.table("dataset.txt",header=T))
```

###Extracting variables of interest

```
Species<-dataframe$Species

TLS<-ordered(as.factor(dataframe$Thoracolumbar_counts))

BTL<-dataframe$Brillouin_TL
```

###Building comparative data object

```
cd<-comparative.data(tree,dataframe,names.col=Species)
```

###Building ‘brunch’ regression object

```
br<-brunch(BTL~TLS,cd)
```

###Outputting results of ‘brunch’ model

```
summary(br)
```

###Producing diagnostic plots for ‘brunch’ model

###Creating empty plotting window for four diagnostic panels

```
par(mfrow=c(2,2))
```

###Plotting diagnostic panels, as in Supplementary Figs 1–5

```
plot(br)
```

###PGLS method – Build dataset file (e.g., dataset.txt) with species names in first column and complexity indices in second and third columns; in this example, we correlate the Brillouin index of the thoracolumbar region with the thoracic:lumbar ratios

```
dataframe<-as.data.frame(read.table("dataset.txt",header=T))
```

###Extracting variables of interest

```
Species<-dataframe$Species

BTL<-dataframe$Brillouin_TL

TLR<-dataframe$TL_ratio

###Building comparative data object

cd<-
comparative.data(tree,dataframe,names.col='Species',vcv=TRUE,vcv
.dim=2)

###Building 'pgls' regression object

pgls<-pgls(BTL~TLR, cd)

###Outputting results of 'pgls' model

summary(pgls)

###Producing diagnostic plots for 'pgls' model

###Creating empty plotting window for four diagnostic panels

par(mfrow=c(2,2))

###Plotting diagnostic panels, as in Supplementary Figs 6, 7

plot(pgls)
```

##

Code for reproducing **Supplementary Figs 8–19 | Robust linear regressions of ancestral values of six complexity indices vs. descendant minus ancestor differences corrected for the regression to the mean. | Robust linear regressions of node ages vs. ancestral values of six complexity indices.**

##

```
###See code for reproducing Fig. 6 above
```

##

Code for reproducing **Supplementary Table 5 | Analyses of skewness applied to five complexity indices.**

##

```
###See code for reproducing Extended Data Fig. 3 above
```

##
Code for organizing data prior to carrying out analyses related to **Fig. 6**, **Supplementary Figs 8–19**, **Extended Data Fig. 2**, and **Supplementary Table 4**.

Code written by Jack Oyston
Milner Centre for Evolution, Department of Biology and Biochemistry, University of Bath,
Claverton Down, Bath, BA2 7AY, UK
##

###Loading `phytools`, `dplyr`, `car`, `PairedData`, `sfsmisc`, `geiger`, `MASS`, and `paleotree` packages

```
require(phytools); require(dplyr); require(car);
require(PairedData); require(sfsmisc); require(MASS)
```

###Importing time-calibrated phylogeny, if not stored already

```
tree<-read.nexus("phy_reordered.nex")
```

###Importing dataset with species names as row names and numeric values of index variable in first column; we use the Brillouin index of the thoracolumbar region as an example
###File Ruta_SourceData_Fig4.xlsx – SHEET 2 (extract tabulation and save it as '.txt' file, e.g., data_Brillouin_index_thoracolumbar_region_reordered.txt)

```
data<-
read.table("data_Brillouin_index_thoracolumbar_region_reordered.
txt",header=T,row.names=1)
```

###Setting names of object to be organized

```
Brillouin_TL<-setNames(data$Brillouin_TL,rownames(data))
```

###Calculating estimated index values at internal nodes of phylogeny using maximum likelihood and storing them as variable 'anc'

```
anc<-fastAnc(tree,Brillouin_TL,CI=F)
```

###Assembling index values of taxa and estimated index values for nodes, storing them as variable 'wt', and organizing all values in column

```
wt<-c(Brillouin_TL,anc)

wt<-cbind(wt)
```

###Numbering rows of values in column

```
rownames(wt)[1:2271]<-c(1:2271)
```

###Creating data frame object preserving numbered rows of values in column and adding numerical identifier for nodes

```
wt<-as.data.frame(wt) %>% mutate(node = as.numeric(rownames(.)))
```

###Creating data frame of numbered tree edges (branches), each delimited by ancestor and descendant nodes

```
edge<-as.data.frame(tree$edge) %>% rename(ancestor_node=V1, descendant_node=V2) %>% mutate(branch=as.numeric(rownames(.)))
```

###Calculating differences between ancestral node and descendant node and combining them with tabulation of node values

```
edge_diff<-inner_join(edge,wt,by=c("ancestor_node"="node")) %>%
rename(ancestor_wt=wt) %>%
inner_join(wt,by=c("descendant_node"="node")) %>%
rename(descendant_wt=wt) %>% mutate(anc_desc_diff=ancestor_wt-descendant_wt)
```

###The tabulation can be re-organized for ease of node identification in relation to major mammal groups. This can be done in different ways. One simple approach involves the following steps. First, the tabulation is re-organized by the increasing value (smallest to largest) of the 'descendant_node' column. Next, a column with names of major groups is added to the tabulation. Names are assigned based upon the number of the 'descendant_node' column. In this respect, it is useful to map node numbers on the phylogeny (e.g., in the ape package).

###Creating vectors of ancestor values, descendant values, and ancestor-descendant differences, with specification of sign difference and absolute value of negative differences

```
anc<-edge_diff$ancestor_wt

desc<-edge_diff$descendant_wt

diff<-edge_diff$anc_desc_diff

pos<-diff[which(diff>0)]

neg<-diff[which(diff<0)]

abs<-abs(neg)
```

###Comparing mean differences and magnitudes of differences

###Unpaired two-sample Wilcoxon two-sided test of difference between mean magnitude of increases and mean absolute magnitude of decreases

```
wilcox.test(pos,abs,alternative=c("two.sided"),paired=FALSE,exac
t=NULL,mu=0,correct=TRUE,conf.int=T,conf.level=0.95)
```

###Unpaired one-sample Wilcoxon two-sided test of mean magnitude of combined increases and decreases

```
wilcox.test(diff,mu=0,alternative="two.sided")
```

###Binomial two-sided test of difference between increases and decreases

```
binom.test(length(pos),length(neg),p=0.5,alternative=c("two.side
d"),conf.level=0.95)
```

###Correlation test of ancestor and descendant values and retrieval of correlation coefficient

```
pearson_anc_desc<-
cor.test(anc,desc,method=c("pearson"),conf.level=0.95)

r<-pearson_anc_desc$estimate
```

###Calculating variances and standard deviations for ancestor and descendant values

```
varanc<-var(anc)

vardesc<-var(desc)

sanc<-sd(anc)

sdesc<-sd(desc)
```

###Applying one of Pitman-Morgan's or Grambsch's tests for equality of variances (note: check assumptions of each test and data distribution)

```
Var.test(anc,desc,alternative=c("two.sided"),ratio=1,paired=TRUE
,conf.level=0.95)

grambsch.Var.test(anc,desc,alternative=c("two.sided"))
```

###Correcting ancestor-differences for regression to the mean artefact

###If null hypothesis of equal variances is not rejected, calculate adjusting term as follows

```
adj<-(2*r*sanc*sdesc)/(varanc+vardesc)
```

###Adjusting differences and building vector of corrected differences

```
D1<-adj*(anc-mean(anc))-(desc-mean(desc))
```

###If null hypothesis of equal variances is rejected, adjust differences as follows (note: 'r' is Pearson correlation coefficient)

```
D1<-r*(anc-mean(anc))-(desc-mean(desc))
```

###From this point, apply code for reproducing Fig. 6 above

###While not used in our paper, the following code can be used to obtain heights for ancestral nodes, descendant nodes, and mid-points of each tree edge; these can be used in correlations with ancestral and/or descendant values and with ancestor-descendant differences

```
node_heights<-as.data.frame(nodeHeights(tree))                %>%
rename(ancestor=V1,descendant=V2)                             %>%
mutate(mid_height=(ancestor+descendant)/2,branch=as.numeric(rown
ames(.)))
```

###For each numbered branch in the tree, the tabulation thus obtained includes the height of the ancestral node of that branch, the height of its descendant node, and the height of the midpoint of the branch

###While not used in our paper, the following code can be used to organize the ages of ancestral nodes, descendant nodes, and mid-points of each tree edge

###Obtaining ages

```
age<-dateNodes(tree)
```

###Joining ages and node numbers

```
node_ages<-as.data.frame(age)                                  %>%
mutate(node=as.numeric(rownames(.)))
```

###For other types of calculations involving node ages, it is handy to bundle together node ages with other variables associated with tree nodes; in the following example, we put together ancestor, descendant, and mid-point node ages with ancestral values, descendant valuesand ancestor-descendant differences for the thoracolumbar Brillouin index

```
node_age_diff<-
inner_join(edge_diff,node_ages,by=c("ancestor_node"="node")) %>%
rename(ancestor_age=age)                                      %>%
inner_join(node_ages,by=c("descendant_node"="node"))          %>%
rename(descendant_age=age) %>% mutate(branch_age=(ancestor_age +
descendant_age)/2)
```

##
